# Glycosylation-Related Genes Predict the Prognosis and Immune Fraction of Ovarian Cancer Patients Based on Weighted Gene Coexpression Network Analysis (WGCNA) and Machine Learning

**DOI:** 10.1155/2022/3665617

**Published:** 2022-03-04

**Authors:** Chen Zhao, Kewei Xiong, Fangrui Zhao, Abdalla Adam, Xiangpan Li

**Affiliations:** ^1^Department of Oncology, Renmin Hospital of Wuhan University, Wuhan, 430060 Hubei Province, China; ^2^School of Mathematics and Statistics, Central China Normal University, Wuhan, 430079 Hubei Province, China; ^3^School of Medicine, Wuhan University, Wuhan, 430072 Hubei Province, China

## Abstract

**Background:**

Ovarian cancer (OC) is a malignancy exhibiting high mortality in female tumors. Glycosylation is a posttranslational modification of proteins but research has failed to demonstrate a systematic link between glycosylation-related signatures and tumor environment of OC.

**Purpose:**

This study is aimed at developing a novel model with glycosylation-related messenger RNAs (GRmRNAs) to predict the prognosis and immune function in OC patients.

**Methods:**

The transcriptional profiles and clinical phenotypes of OC patients were collected from the Gene Expression Omnibus and The Cancer Genome Atlas databases. A weighted gene coexpression network analysis and machine learning were performed to find the optimal survival-related GRmRNAs. Least absolute shrinkage and selection operator regression (LASSO) and Cox regression were carried out to calculate the coefficients of each GRmRNA and compute the risk score of each patient as well as develop a prognostic model. A nomogram model was constructed, and several algorithms were used to investigate the relationship between risk subtypes and immune-infiltrating levels.

**Results:**

A total of four signatures (ALG8, DCTN4, DCTN6, and UBB) were determined to calculate the risk scores, classifying patients into the high-and low-risk groups. High-risk patients exhibited significantly poorer survival outcomes, and the established nomogram model had a promising prediction for OC patients' prognosis. Tumor purity and tumor mutation burden were negatively correlated with risk scores. In addition, risk scores held statistical associations with pathway signatures such as Wnt, Hippo, and reactive oxygen species, and nonsynonymous mutation counts.

**Conclusion:**

The currently established risk scores based on GRmRNAs can accurately predict the prognosis, the immune microenvironment, and the immunotherapeutic efficacy of OC patients.

## 1. Introduction

Ovarian cancer (OC) is one of the most common gynecological neoplasms with the fourth highest incidence and third highest mortality in the world [1]. Consecutive ovulation, low immunity, hormonal fluctuations, and aberrant reactive oxygen production can contribute to the pathogenesis [2]. In addition, the tumorigenesis of OC is reported to be highly associated with BRCA1 dysfunction [3, 4]. Since ovaries are located in deep pelvic cavities, it is difficult to detect OC in the early stages [5]. Consequently, almost 70% of OC patients have already progressed to advanced stages with distant metastases present at the time of diagnosis [6, 7]. Despite advances in OC therapies, the 5-year survival rate for OC is still less than 50%, which is significantly lower than the 85% rate for breast cancer [8]. Approximately 70% of OC individuals will develop a recurrence after surgery, and about 75% of high-grade serous ovarian cancer (HGSOC) patients will experience chemoresistance against cisplatin, oxaliplatin, carboplatin, etc. [9, 10]. Identifying novel risk factors that regulate tumorigenesis, migration, and proliferation will contribute to early diagnosis and personalized interventions for OC treatment. Hence, it is imperative to explore the biological pathologic mechanisms and develop a reliable prognostic prediction model for OC patients.

Glycosylation is a posttranslational modification of proteins; the main types of which are N-linked and O-linked occurring in the endoplasmic reticulum (ER) and Golgi complex, respectively; it demonstrates complicated mechanisms due to its variations based on the expression of glycosylating enzymes [11]. The transfer of N-acetyl glucosamine phosphate to the dolichol phosphate can result in N-linked glycosylation, which is mediated by various glycosyltransferases in the ER [12]. O-linked glycosylation is more complex than the N-linked one for the unknown initiation emerging from the consensus sequence [12]. The O-linked modification pattern takes place in the intracellular nuclear and cytoplasmic compartments and does not elongate to create complex structures like other types of glycosylation [13]. It has been reported that glycosylation plays a regulatory role in cell differentiation, neoplastic progression, and immune control of malignant tumors [14, 15]. Aberrant glycosylation has been considered an important indicator of immune modulation induced by tumor and metastasis since it can generate antigens as targets for tumor-specific T cells [16, 17]. Several lines of evidence from clinical practice of OC suggest that glycosylation changes in proteins such as immunoglobulin G, *α*_1_-acid glycoprotein, and ceruloplasmin. All of this contributes to promoting or obstructing tumorigenesis and invasion [18, 19].

There are also emerging prognostic models for OC with biomarkers associated with post-translational regulatory mechanisms such as alternative splicing and N6-methyladenosine modification [20, 21]. However, as an important hallmark among over 300 protein posttranscriptional modifications [22], only a few studies regarding glycosylation in OC have been reported. A translational study revealed that the expression levels of 210 glycosyltransferase genes could distinguish six cancer types, including breast, ovarian, glioblastoma, kidney, colon, and lung [23]. Pan et al. identified novel subtypes of HGSOC with glycoproteomics-based signatures for clinical prediction using consensus clustering and verified that the variation in glycan types would coordinate with tumor heterogeneity based on proteomics [24]. Recently, a prognostic model based on glycosylation-related genes for proficient mismatch repair in colorectal cancer has been proposed, from which it can be inferred that glycosylation is capable of serving as a hallmark for prognostic prediction [25]. Nonetheless, the mentioned research has failed to demonstrate a systematic link between glycosylation-related signatures and tumor environment of OC.

With these inadequacies and challenges of OC research, this research is aimed at investigating the clinicopathologic features of glycosylation-related messenger RNAs (GRmRNAs) for the prognostic and tumor microenvironment (TME) prediction of patients with OC. Based on the established risk prognostic model, the associations with risk scores, tumor immune-infiltration, and hallmark signatures were analyzed. Furthermore, we also explore the correlations between risk subtypes and mutation characteristics. These findings were based on transcriptomics to provide a novel insight into the role of glycosylation in ovarian cancer and contribute to precision treatment.

## 2. Materials and Methods

### 2.1. Data Acquisition and Processing

All the datasets included in this study were available online to the public. The transcriptional RNA sequencing (RNA-seq) data of patients with ovarian cancer, including 427 samples, clinicopathologic data, and simple nucleotide variation (SNV) information were retrieved from The Cancer Genome Atlas (TCGA, https://portal.gdc.cancer.gov/). The RNA-seq profile in fragments per kilobase million (FPKM) was processed into log2-transformed transcripts per million (TPM). Another cohort of gene expression data of 101 aggregated samples and corresponding clinical characteristics were obtained from GSE63885 [26] in the Gene Expression Omnibus (GEO, https://www.ncbi.nlm.nih.gov/geo/) database. A total of 636 glycosylation-related (GR) genes (Supplementary Table 1) were downloaded from the Molecular Signatures Database (MSigDB, http://www.gsea-msigdb.org/gsea/msigdb/), a web-based assembling of annotated gene sets for biologic function analysis. The levels of tumor immune-infiltration estimated by different methods containing CIBERSORT, CIBERSORT-ASB, QUANTISEQ, MCPCOUNTER, XCELL, and EPIC were extracted from the TIMER (http://timer.cistrome.org) [27] web server for TME investigations.

### 2.2. Identification of Prognostic GRmRNAs

A weighted gene coexpression network analysis (WGCNA) [28] was performed on GSE63885 based on its expression levels and phenotypes with follow-up time, vital status, tumor grade, FIGO stage, tumor size, and clinical status (Supplementary Table 2) to screen for hub genes. We quantified the goodness of fit by using a scale-free topology model and integrating it with mean connectivity to determine the optimal soft threshold. Multiple modules were detected automatically at first, and then, the topological overlap measure was calculated to estimate the adjacencies and similarities among different modules subjected to average hierarchical clustering by the measurement of Euclidean distance. Namely, topologically similar modules were combined into a neocluster. A correlation exploration was performed to assess the correlations between module genes and phenotypes. Modules with relatively strong positive correlations with survival time and vital status were selected. Then, overlapping genes of WGCNA and glycosylation were identified for Kaplan-Meier (KM) analysis. The optimal cutoff of each GRmRNA was determined by the “survminer” package, and patients were divided into the high- and low-expression groups. Only significant signatures by the log-rank test were considered to have prognostic implications and were enrolled in the study. We then used five machine learning methods to estimate the importance of survival associated with GRmRNAs, including two linear models involving least absolute shrinkage and selection operator (LASSO) regression [29] and ridge regression [30], besides a nonlinear model (XGBoost) [31], an ensemble learning method (random forest) [32], and a boosting algorithm (AdaBoost) [33]. GRmRNAs with relatively higher weight were considered to contribute to the prognosis of OC patients.

To further determine the GRmRNAs responsible for the prognosis and establish a prognostic risk model, we randomly classified patients into a training set and a test set at a ratio of 11 : 9 using the “caret” package [34], and the randomness was verified by a chi-square test. The training set was submitted to LASSO Cox regression to screen for optimal GRmRNAs (OGRmRNAs) at the least of partial likelihood deviance. Based on the regression coefficients and the expression levels of OGRmRNAs, the formula for risk scores could be formed as

(1) $$\begin{array}{c} \text{Risk scor}{\text{e}}_{j}=\overset{n}{\underset{i=1}{\sum }}\alpha_{i}k_{ij}, \end{array}$$

where *α*_*i*_ represents the regression coefficient of the *i*th gene, and *k*_*ij*_ represents the expression of the *i*th gene in the *j*th sample. Patients were then stratified into the high- and low-risk groups at the median cutoff of risk scores. The differences between the two risk subtypes were estimated by the log-rank test in both the training and test sets.

### 2.3. Estimation of Model Effectiveness and Establishment of Combined Diagnosis

The differences in age, stage, and grade between risk subtypes were estimated by a chi-square test. Principal component analysis (PCA) and *t*-distribution stochastic neighbor embedding (tSNE) algorithms were utilized to evaluate the ability of discrimination in the developed model. To determine the independent prognostic value of the model, we conducted a univariate and multivariate Cox proportional hazard regression with age, stage, grade, and risk scores. Furthermore, a nomogram was created to predict the probability of 1-, 3-, and 5-year survival of OC patients. A decision curve analysis (DCA) was performed to ascertain the net benefits of the prognostic risk model in clinical practice.

We also estimated the expression differences of OGRmRNAs between normal tissues and tumor tissues in OC with data retrieved from UCSC Xena (http://xena.ucsc.edu/) in TPM formation. To reveal the stemness feature, the mRNA expression-based stemness index (mRNAsi) was computed using one-class logistic regression with a machine learning algorithm [35] and compared between the high- and low-expression groups of OGRmRNAs at the median cutoff. Coexpressed genes with OGRmRNAs were determined with a threshold of 0.7, and they were enrolled in Gene Ontology (GO) containing biological processes, cellular components, and molecular functions, and Kyoto Encyclopedia of Genes and Genomes (KEGG) functional enrichment analysis. Single-sample gene set enrichment analysis (ssGSEA) [36] was employed to quantify the correlations between OGRmRNA expression levels and tumor immune infiltrating. Moreover, GSEA was performed with the gene matrix of “c2.cp.kegg.v7.4.symbols.gmt” to identify the significantly enriched pathways in the high- and low-risk groups, respectively, in the application GSEA (version 4.0.3) downloaded from MSigDB, and we cross-checked the results with the “clusterProfiler” package [37].

### 2.4. Investigations on the Correlation of TME and Risk Scores

TME has important implications for the regulatory mechanisms of immune cells. We mainly investigated the relationships between prognostic risk and the depth of immune-infiltration. The TIMER database was used to explore the correlations between risk scores and immune responses quantified by six methods. The differences in immune function and infiltration of leukocytes between the high- and low-risk groups were estimated by performing the ssGSEA [36] algorithm. In addition, the Pearson correlation coefficients between risk scores and distinct tumor immune infiltrations as well as the proportion of infiltrations in each sample with statistical significance were calculated by the CIBERSORT algorithm (1000 permutations). Moreover, the Wilcoxon rank-sum test was applied to compare the expression differences of typical immune checkpoint molecules between the two risk subtypes. The information on immune subtypes of OC was obtained from Thorsson's study [38], in which the overlapping immune subtypes included C1 (wound healing), C2 (IFN-gamma dominant), and C4 (lymphocyte depleted). The differences in risk scores among the three subtypes were measured. In addition, to quantify the relationship between tumor purity and risk scores, we employed “estimation of stromal and immune cells in malignant tumors using expression data” (ESTIMATE) algorithm [39] including three types of scores: immune score, stromal score, which represented the nontumor proportion, and their addition consisting of the ESTIMATE score.

### 2.5. Exploration of Risk Subtypes and Molecular Characteristics

The difference in tumor mutational burden (TMB) between the high- and low-risk groups was analyzed. Furthermore, we collected several important pathway signatures potentially interacting with OC, including Wnt, Hippo, Hedgehog, Notch, TGF-*β*, PI3K/Akt, EMT, JAK_STAT, interleukin-8, NF-*κ*B, interferon, and ROS (Supplementary Table 3). Gene set variation analysis (GSVA) was adopted to calculate the enrichment score, which was then used to quantify the connection between risk scores and pathways. The landscape of top mutated genes in the two risk groups was shown with their mutation types and frequencies by maftools. Afterward, multiple mutation types were stratified into two novel statuses, including nonsynonymous mutation and synonymous mutation [40]. The changes between the low- and high-risk scores were examined by correlation coefficients and different tests.

### 2.6. Statistical Analysis

All statistical tests and bioinformatics analysis were conducted by R (versions 3.6.3 and 4.1.1), including the two-sample Wilcoxon rank-sum test and Kruskal-Wallis for continuous data, Pearson chi-square test and Fisher's exact test for categorical data, log-rank test for KM analysis, and (LASSO) Cox proportional hazard regression to estimate the hazard ratios (HRs) and 95% confidence interval (CI). For correlation explorations, the Pearson correlation coefficients were used. Machine learning predictive models were developed by Python (version 3.8.0) libraries “XGBoost (version 1.2.1)” and “sklearn (version 0.22.1),” technical details of which have been described previously. A two-tailed *P* < 0.05 for all unadjusted comparisons and an adjusted *P* < 0.05 for functional enrichment analysis were considered statistically significant.

## 3. Results

### 3.1. Identification of Prognostic GRmRNAs

**Figure 1:**
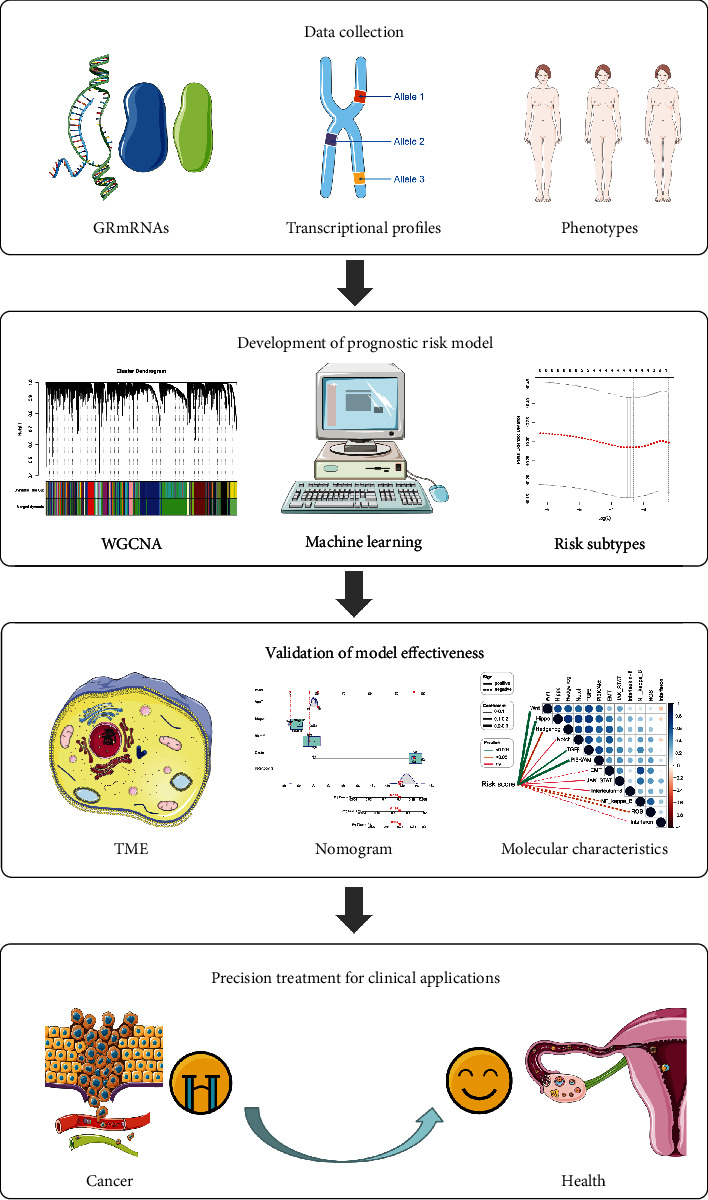
The workflow of this study.

**Figure 2:**
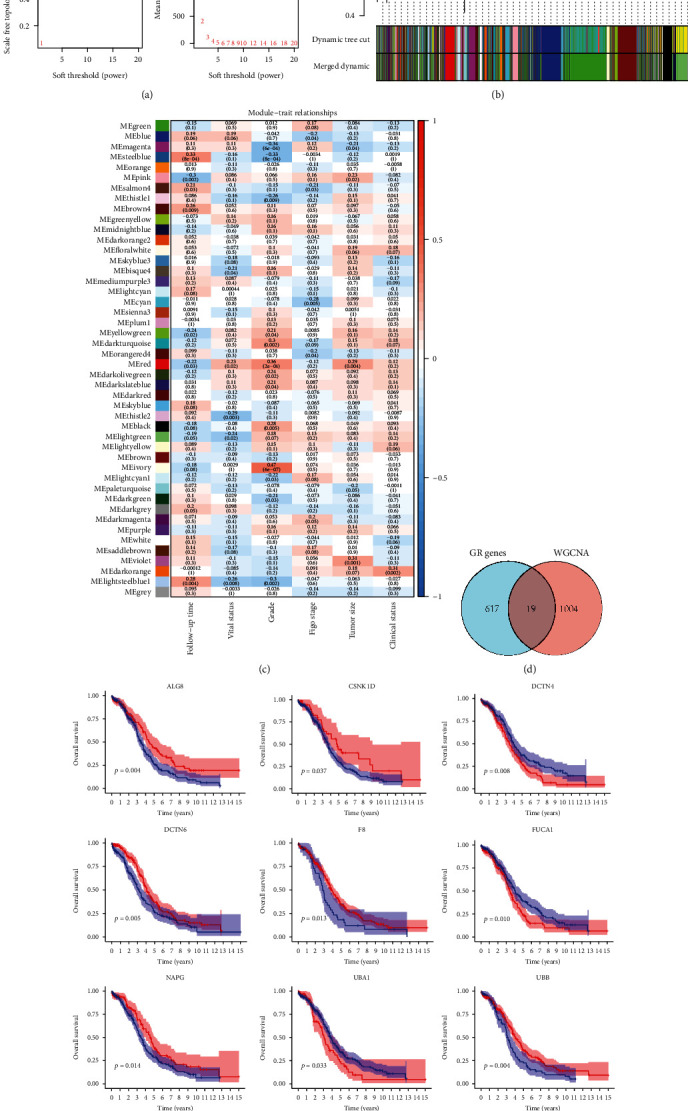
Discovery of prognostic GRmRNAs by WGCNA. (a) The distribution and trends of scale free topology model fit and mean connectivity along with soft threshold. (b) The clustering of genes among different modules by the dynamic tree cut and merged dynamic method. The gray modules represent unclassified genes. (c) The average correlations among multiple modules and clinical features. The colors of the cells indicate the strength of the correlation, and the numbers in parentheses represent the *P* value of the correlation test. (d) 19 overlapped GRmRNAs from the glycosylation-related gene list and WGCNA were obtained. (e) Significant survival differences between the high- and low-expression groups of GRmRNAs by log-rank test (GRmRNAs: glycosylation-related mRNAs; WGCNA: weighted gene coexpression network analysis).

The workflow of this study is shown in Figure 1. In WGCNA, we determined the soft threshold of 6 by calculating the scale-free model fit and mean connectivity (Figure 2(a)). Different module genes in the dynamic tree cut were reclustered through a topological similarity strategy, where genes were assembled into fewer modules as shown in Figure 2(b). The relationships between modules and clinical phenotypes implied that the modules in steel blue (*r* = 0.33, *P* < 0.001), brown4 (*r* = 0.26, *P* = 0.009), and light steel blue1 (*r* = 0.28, *P* = 0.004) had relatively strong correlations with follow-up time. The red module had a slightly positive correlation with survival status (Figure 2(c)), genes which were used to be analyzed in the following steps. As shown in Figure 2(a), a total of 19 overlapped genes from the glycosylation-related gene list and WGCNA were obtained. By performing KM analysis, 9 prognostic GRmRNAs, including ALG8 (*P* = 0.004), CSNK1D (*P* = 0.037), DCTN4 (*P* = 0.008), DCTN6 (*P* = 0.005), F8 (*P* = 0.013), FUCA1 (*P* = 0.010), NAPG (*P* = 0.014), UBA1 (*P* = 0.033), and UBB (*P* = 0.004) exhibited statistical significance in survival differences at the optimal cutoff (Figure 2(e)).

### 3.2. Determination of OGRmRNAs and Validation

**Figure 3:**
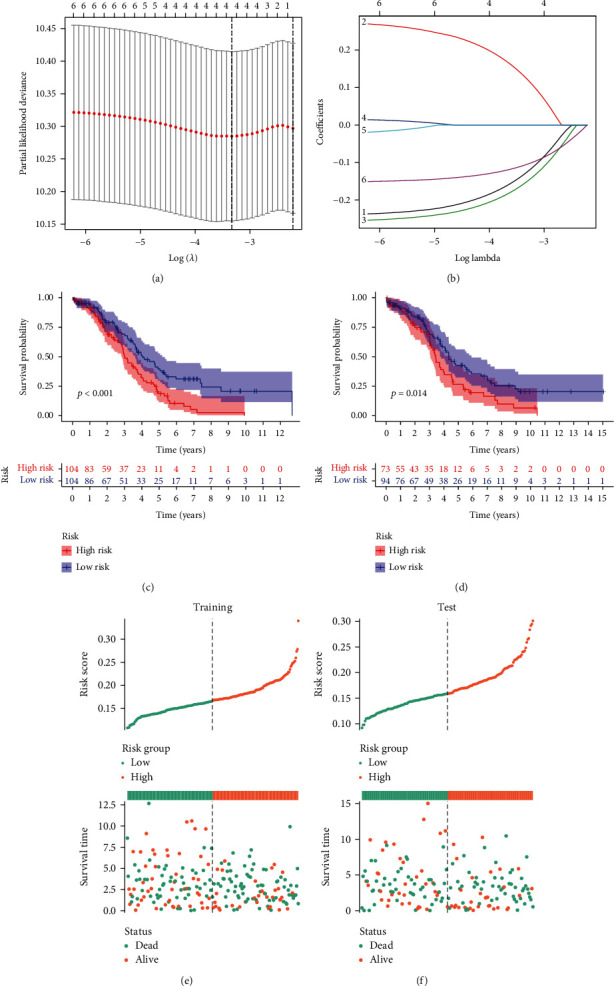
Screening for OGRmRNAs. (a) Profiles of LASSO regression regarding partial likelihood deviance, lambda. The lines indicate the 95% confidence interval of the regression, and the dotted line represents the optimal number of variables. (b) The association with regression coefficients and log-transformed lambda. Each line represents a variable. (c, d) Survival differences between the high- and low-risk groups in the training set and test set. The table below the survival curves represents the number of patients alive in each year. (e, f) The risk curves and the distribution of patients in the training set and test set. Samples were ordered according to the risk scores from low to high. The dots in the lower part represent the distribution of cases. (OGRmRNAs: optimal glycosylation mRNAs; LASSO: least absolute shrinkage and selection operator).

**Figure 4:**
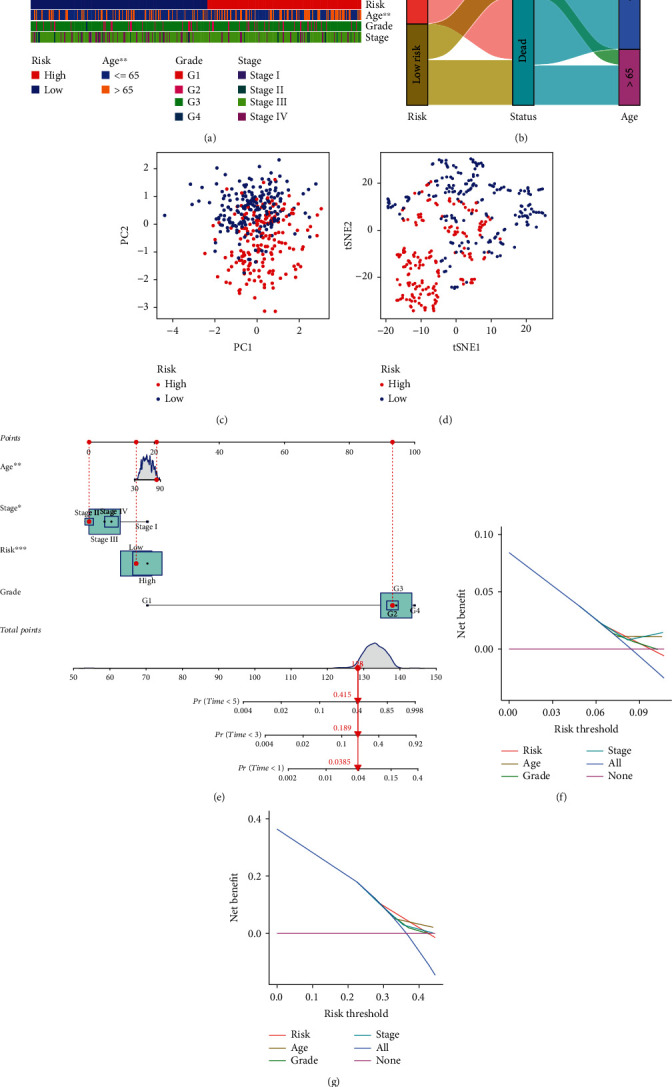
Estimation of model effectiveness and a nomogram prediction. (a) Different stratifications of clinical phenotypes in the high- and low-risk groups. (b) Connections among risk subtypes, vital status, and age stratifications. (c, d) Principal component analysis and *t*-distribution stochastic neighbor embedding for sample discrimination in the first two dimensions. (e) Nomogram for 1-, 3-, and 5-year overall survival prediction. The red line shows an example of how to predict the prognosis. (f, g) DCA of 1-year and 3-year survival probability. The upper lines indicate more net benefit (^∗∗∗^*P* < 0.001, ^∗∗^*P* < 0.01, and ^∗^*P* < 0.05).

The detected randomness of the split for the training set and test set is shown in Table 1. LASSO regression, ridge regression, XGBoost, random forest, and AdBoost were utilized to sort the importance of weights in the prognosis based on 9 survival-related GRmRNAs. As shown in Table 2, ALG8, DCTN4, DCTN6, F8, NAPG, and UBB held greater weight and they were included to develop the prognostic risk model. A LASSO Cox regression was performed and four OGRmRNAs were selected depending on the optimal value of lambda (Figures 3(a) and 3(b)). Hence, the risk scores could be calculated according to the formula:

(2) $$\begin{array}{c} \text{Risk score}=-0.1277\times E\left(\text{ALG}8\right)+0.1256\times E\left(\text{DCTN}4\right)-0.1528\times E\left(\text{DCTN}6\right)-0.1058\times E\left(\text{UBB}\right), \end{array}$$

where *E*(∙) represents the expression of OGRmRNAs. Patients were then separated into the high-risk and low-risk groups at the median value of their risk scores (Supplementary Table 4). KM analysis revealed that OC patients experienced significantly different survival outcomes in both the training set (*P* < 0.001, Figure 3(c)) and the test set (*P* = 0.014, Figure 3(d)). Risk curves also indicated that patients with low risk had better survival outcomes (Figures 3(e) and 3(f)). The chi-square test illustrated that old patients had significantly higher risk scores than those under 65 years of age (*P* < 0.01, Figure 4(a)). The Sankey diagram showed the degree of connection among risk subtypes, survival status, and age that old patients were more likely to have worse outcomes (Figure 4(b)). PCA and tSNE demonstrated that patients were differentiated well in two dimensions based on the risk scores (Figures 4(c) and 4(d)), which indicates that the model has a promising ability to stratify risk subtypes. By performing Cox regression, we found that age (hazard ratio (HR) = 1.021, 95%confidence interval (CI) = 1.006-1.036, *P* = 0.006) and risk scores (HR = 1.540, 95%CI = 1.101-2.154, *P* = 0.012) could serve as independent prognostic factors (Table 3). A nomogram model was established to predict 1-, 3-, and 5-year survival, where age (*P* < 0.01), stage (*P* < 0.05), and risk (*P* < 0.001) demonstrated significance (Figure 4(e)). The DCA curves analysis nomogram was performed, implying that the combined model of 1-year and 3-year survival probability showed the optimal net benefit compared to a single indicator (Figures 4(f) and 4(g)).

### 3.3. Validation of OGRmRNAs

**Figure 5:**
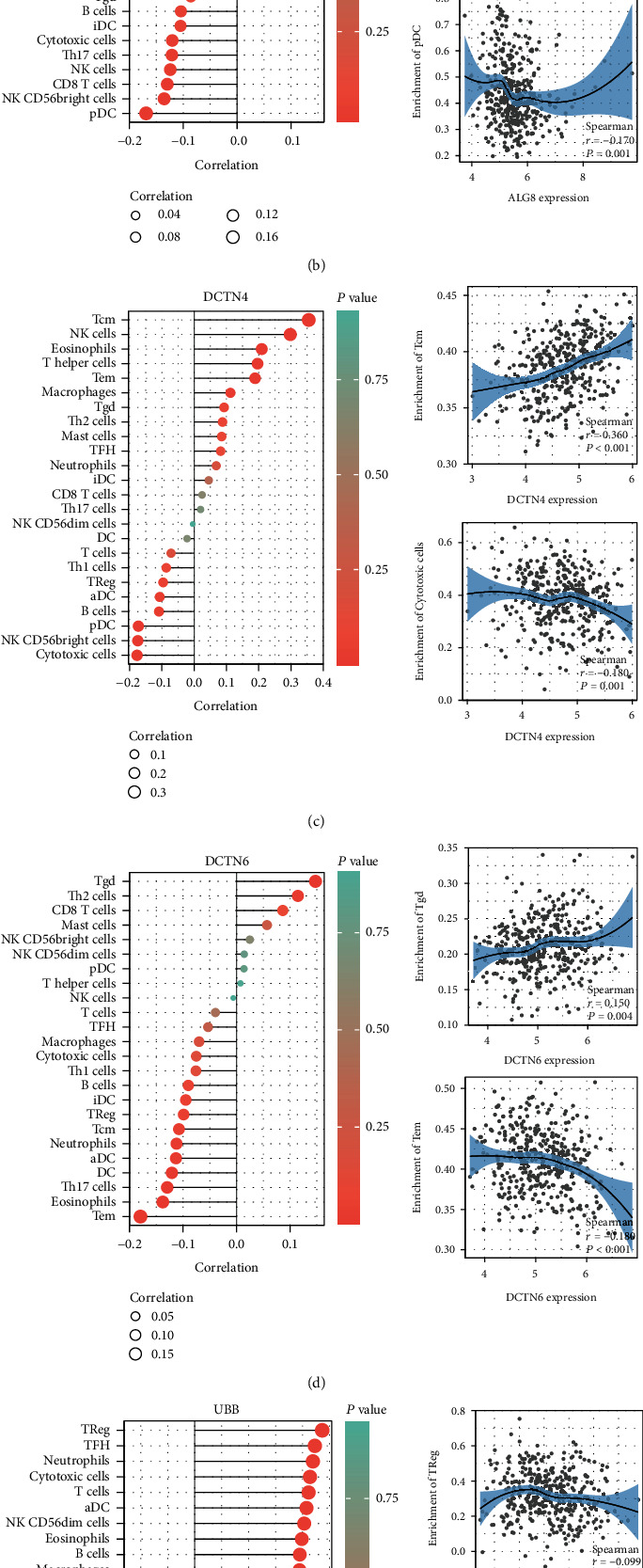
Validation of OGRmRNAs. (a) Expression changes of GRmRNAs between normal and tumor tissues. The lines on the bars indicate the values between the median plus and minus the standard error. (b–e) Associations between OGRmRNAs and immune-infiltrating levels. The color represents the significance. The redder, the more significant. The circle size represents the correlation coefficients (OGRmRNAs: optimal glycosylation mRNAs; ^∗∗∗^*P* < 0.001).

The Wilcoxon rank-sum test showed that ALG8 (*P* < 0.001) had a higher expression level in tumor tissues compared with normal tissues, while DCTN4 (*P* < 0.001), DCTN6 (*P* < 0.001), and UBB (*P* < 0.001) had lower expression levels in tumor tissues (Figure 5(a)). According to immune-infiltrating results, OGRmRNAs presented significantly positive correlations with immune cells such as helper T cells (Th, *r* = 0.15, *P* = 0.005), central memory T cells (Tcm, *r* = 0.36, *P* < 0.001), and gamma delta T cells (Tgd, *r* = 0.15, *P* = 0.004) but showed negative connections with plasmacytoid dendritic cells (pDC, *r* = −0.17, *P* = 0.001), cytotoxic cells (*r* = −0.18, *P* = 0.001), effective memory T cells (Tem, *r* = −0.18, *P* < 0.001), and Tcm (*r* = −0.11, *P* = 0.037) shown in Figures 5(b)–5(e). According to the KW test, overexpressed ALG8 exhibited significantly higher mRNAsi, whereas upregulated DCTN4 presented lower mRNAsi (*P* < 0.001, Supplementary Figure 1A-B). And all the OGRmRNAs exhibited significantly different mRNAsi scores among the low-expression groups, high-expression groups, and normal tissues (*P* < 0.001, Supplementary Figure 1A-D). A total of 225 coexpressed protein coding genes were determined for functional enrichment analysis (Supplementary Table 5). GO terms regarding the metabolic process, catabolic process, nuclear movement (Supplementary Figure 2A), and KEGG pathways of spliceosome and RNA degradation (Supplementary Figure 2B) demonstrated significance.

### 3.4. Biological Characteristics and TME Investigation

**Figure 6:**
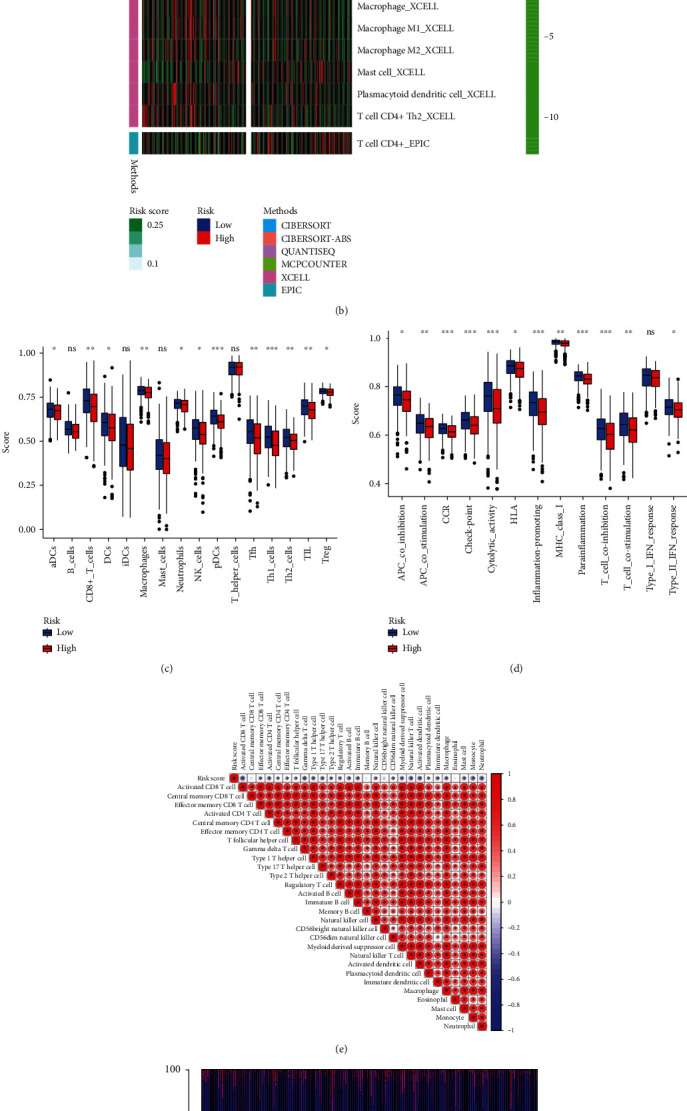
Biological functions and TME landscape. (a) Significant enriched pathways in the high- and low-risk groups. The extremum located in the left part indicates a positive association between risk scores and pathway activity, and vice versa. (b) The relationships of risk and tumor immune-infiltrations according to the evidence from the TIMER database. (c, d) The differences of tumor infiltrating of 16 cell types and score of immune pathways between the risk groups by ssGSEA. The lines in the boxes represent the median values. The black dots represent outliers. Asterisks indicate significance. (e) The associations between risk and immune-infiltrations by CIBERSORT algorithm. (f) Proportions of multiple tumor-infiltrating cells (TME: tumor microenvironment; ssGSEA: single-sample gene set enrichment analysis).

To explore the biological functions, GSEA was performed in the high- and low-risk groups, respectively. It demonstrated that high-risk agents were enriched in the “phosphatidylinositol signaling system” while the low-risk genes were significantly enriched in “oxidative phosphorylation” and “proteasome” (Figure 6(a)). The results obtained from the “clusterProfiler” of R foundation are provided in Supplementary Table 6, which indicates that the high-risk group appeared to inhibit pathways such as “ribosome,” “systemic lupus erythematosus,” and “type I diabetes mellitus” but no significant activated terms. The landscape of antitumor immunity was investigated using expression data of OC patients. As shown in Figure 6(b), tumor immune infiltration levels between the high- and low-risk groups were slightly different. It was notable that macrophages, plasmacytoid dendritic cells, and CD4^+^ Th2, etc. showed lower infiltration abundance in the high-risk subtype, whereas mast cells had higher levels in the high-risk subtype. Two distinctive patterns of immune infiltrations could be observed in the high- and low-risk groups by the Wilcoxon rank-sum test. Decreased levels of tumor infiltration of major immune cells (Figure 6(c)) such as CD8+ T cells (*P* < 0.01), macrophages (*P* < 0.01), Th1 cells (*P* < 0.001), and tumor infiltrating lymphocytes (TILs, *P* < 0.01), also decreased levels of immune pathways (Figure 6(d)) such as cytolytic activity (*P* < 0.001), inflammation-promoting (*P* < 0.001), and coinhibitions of T cells (*P* < 0.001) in the high-risk group were reported. By performing the CIBERSORT algorithm and excluding samples with no statistical significance, we found that risk scores wielded negative correlations with almost all tumor infiltrations (Figure 6(e)). The proportions of immune infiltrations from 22 cell types in the two risk subtypes were shown in a bar plot (Figure 6(f)).

### 3.5. Relationships between Molecular Features and Risk

**Figure 7:**
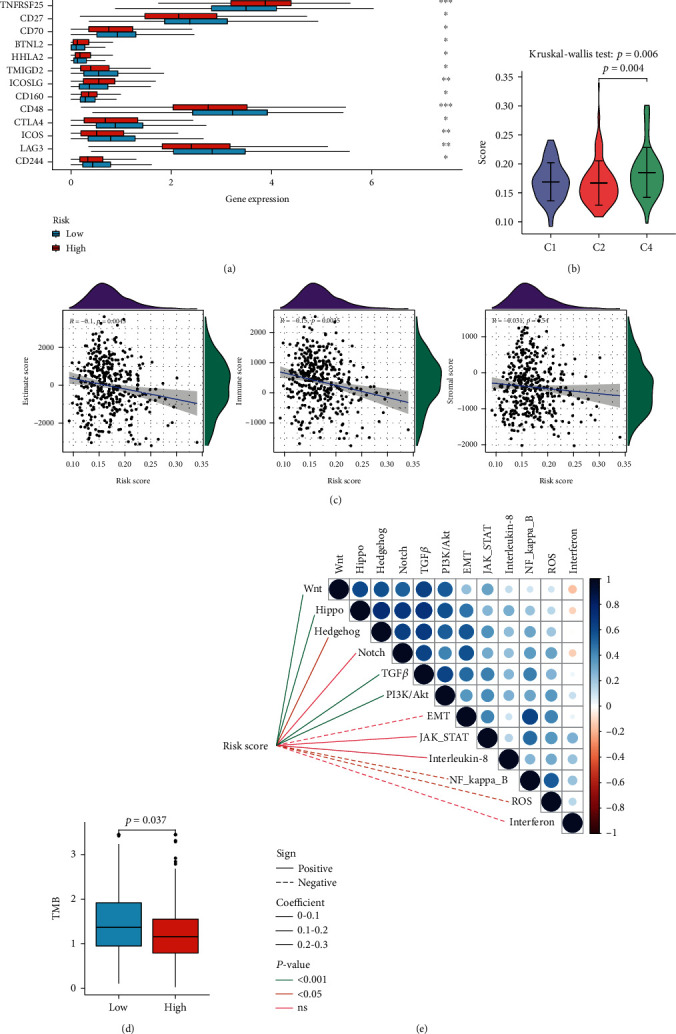
Molecular features in risk stratification. (a) The differences of expression levels of immune checkpoints between the high- and low-risk subtypes. The lines inside the boxes represent the median values, and the lines outside the boxes indicate the 95% confidence interval. (b) Relationships between immune subtypes and risk scores. The short horizontal lines represent the median values, and the vertical lines indicate the 95% confidence interval. (c) The correlation between tumor purity and risk scores. The blue lines represent fitted lines, and the gray area represents the 95% confidence interval. The mountain graphs at the top and stuck to the right represent the density of distribution. (d) TMB difference in the high and low groups. The lines inside the boxes represent the median values, and the lines outside the boxes indicate the 95% confidence interval. The black dots show the outliers. (e) Correlations of risk scores and enriched pathways. The circle in the thermogram shows the correlations among the signaling pathways. Blue represents positive correlations, while red indicates negative ones.

We compared several immune checkpoint expression levels between the high- and low-risk groups by the Wilcoxon rank-sum test (Supplementary Table 7). It revealed that targets such as TIGIT (*P* < 0.05), TNFRSF25 (*P* < 0.001), CD27 (*P* < 0.05), and CD70 (*P* < 0.05) exhibited significant differences (Figure 7(a)). The KW test demonstrated that patients in the C4 had higher risk scores than those in C1 and C2 (*P* = 0.006, Figure 7(b)). Tumor purity was estimated by ESTIMATE scores, which is composed of immune scores and stromal scores. The risk scores were negatively associated with ESTIMATE scores (*r* = −0.1, *P* = 0.043) and immune scores (*r* = −0.15, *P* = 0.004), whereas there was no statistical significance for stromal scores (Figure 7(c)). TMB, a biomarker of immune checkpoint inhibitor therapies, had different levels between the high- and low-risk groups (*P* = 0.037, Figure 7(d)). In addition, the high-risk group presented significantly activated pathway signatures including Wnt (*P* < 0.001), Hippo (*P* < 0.001), Hedgehog (*P* < 0.05), TGF-*β* (*P* < 0.001), and PI3K/Akt (*P* < 0.001) while inhibited signatures include NF-*κ*B (*P* < 0.001) and ROS (*P* < 0.05, Figure 7(e)).

### 3.6. Mutation Landscape Analysis

**Figure 8:**
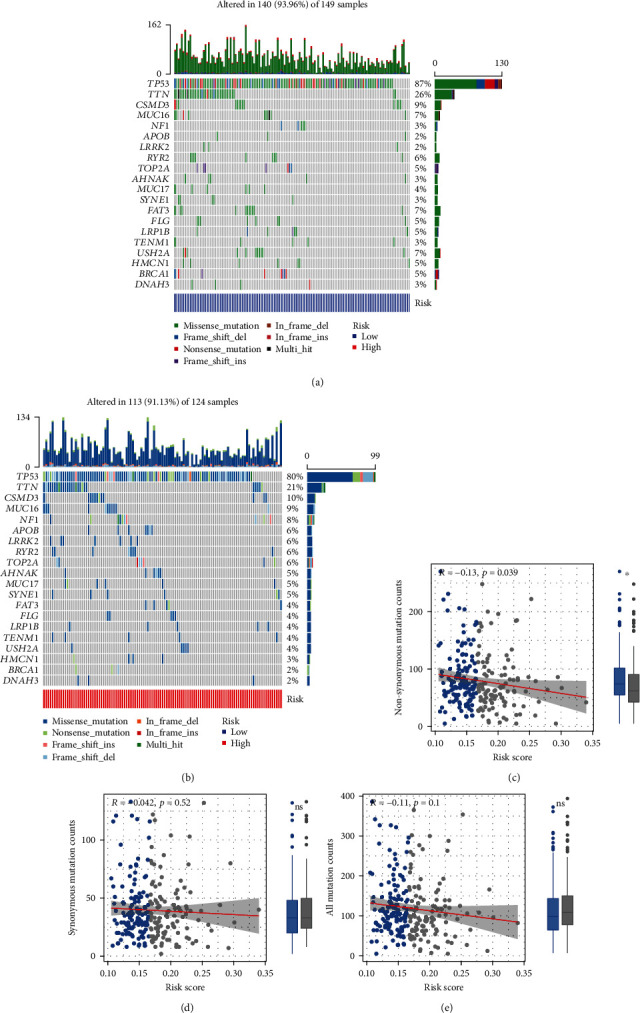
Mutation landscape. (a, b) The profiles of the top-25 mutated genes in the high-and low-risk groups. The upper bar shows the total gene mutation amount and corresponding mutation types. The right bar shows the mutation frequency of the top 25 mutated genes. (c–e) Associations of nonsynonymous mutation counts, synonymous mutation counts, all mutation counts, risk scores, and their variations between the low- and high-risk groups. The red lines represent the fitted lines, and the gray area represents the 95% confidence interval. The dots outside the boxes show the outliers (^∗^*P* < 0.05; ns: no significance).

Furthermore, we examined the mutation profiles of risk. As shown in Figures 8(a) and 8(b), TP53, TTN, and CSMD3 had the highest mutation frequency with the most missense mutation, followed by MUC16. Patients in the high-risk group had a lower frequency of TP53 mutations. After classifying different mutation types into nonsynonymous mutation and synonymous mutation, we estimated their associations with risk scores. It was illustrated that risk scores had a slightly negative link with non-synonymous mutation counts (*r* = −0.13, *P* = 0.039; Figure 8(c)), while risk scores exhibited no significant linear correlations with synonymous mutation counts and all mutation counts (Figures 8(d) and 8(e)).

## 4. Discussion

In this study, a total of four GRmRNAs, including ALG8, DCTN4, DCTN6, and UBB, were selected for the development of the prognostic risk model. The relationship between glycosylation and ALG8 has been studied, particularly in congenital disorders of glycosylation (CDG). The point mutations or small deletions of ALG8 will lead to an unfavorable prognosis [41]. It has been reported that ALG8 could perform as a variate of a prognostic model for gastric cancer [42]. Amplified hotspots on 11q14.1 (NDUFC2, ALG8, and USP35) led to poor prognosis in estrogen receptor-negative breast cancer [43]. Our study found that overexpressed ALG8 was located in OC tissues and was associated with favorable survival outcomes. A similar result for CXCL11 was also illustrated in colorectal cancer [44]. According to previous evidence and this study, we speculate that ALG8 is correlated with a higher proportion of antitumor immune cells, and a lower proportion of protumor immune cells in OC. Nonetheless, further studies should be performed.

A previous study has revealed that DCTN4 was upregulated in colon adenocarcinoma and high expression was associated with prolonged overall survival [45]. DCTN6 has high expression in low-grade glioma but it is associated with unfavorable survival outcomes [46]. Conversely, DCTN4 and DCTN6 were both observed to be downregulated in tumor tissues of OC compared with adjacent tissues in this study. Overexpressed DCTN4 was associated with poor survival, while DCTN6 was correlated with a satisfactory result for overall survival. The relevant results of UBB were similar to DCTN6. However, to determine the role of inhibition or promotion of cancer, analyzing the expression levels is insufficient since more investigations to confirm the biological functions should be undertaken. Subsequently, LASSO Cox regression was used to compute the coefficients of each gene mentioned above and develop a prognostic risk system, which could be considered an independent prognostic factor. High-risk patients had a significantly worse prognosis than those in the low-risk group, and more individuals over 65 years of age were from the high-risk group, which is consistent with the previous findings [47]. The functional enrichment exploration demonstrated that the high-risk agents were enriched in the “phosphatidylinositol signaling system.” Phosphatidylinositol-associated signaling pathways play a vital role in tumor cell apoptosis, proliferation, invasion, and metabolism [48, 49]. The genes in the low-risk group were mainly enriched in “oxidative phosphorylation” and “proteasome.” The oxidative phosphorylation pathway in tumors and the tumor microenvironment is recognized as a target for novel anticancer therapies. The multimeric complexes of the oxidative phosphorylation pathway are targets for small-molecule inhibitors, which can inhibit metabolism, induce oxidative damage, and lead to cancer cell death. It is indicated that strategies to interfere with oxidative phosphorylation should be considered for the treatment of ovarian tumors [50]. Meanwhile, proteasome activity has been linked to tumor metastasis, and therapy based on inhibiting the proteasome and HDAC6 has been proposed as an underlying strategy for OC treatment [51, 52]. Simultaneously, risk scores were positively correlated with Wnt, Hippo, Hedgehog, TGF-*β*, and PI3K/Akt pathways. In contrast, risk scores demonstrated a negative correlation with NF-*κ*B and reactive oxygen species. These results may contribute to studying the interplay between the signaling pathways and different risk subtypes of OC patients.

Recently, it has been verified that tumor cells avoid being killed by immune cells with the aid of glycosylation in the TME [53]. The explicit interaction of antigens with antibodies is the foundation of the immune response. Glycosylated antigen-specific antibodies are beneficial in cancer therapy by augmenting immunity [54, 55]. To unravel the connection between glycosylation and TME of OC, we used multiple methods to quantify the immune-infiltration levels of leukocytes and the fraction of immune pathways. OC patients of the high-risk group demonstrated increased neutrophils and mast cells, but the majority of cases showed decreased cell types such as activated natural killer (NK) cells, CD8^+^ T cells, Th1/2 cells, and macrophages. The results of activated NK cells and macrophages were consistent with previous studies [56, 57] but showed contrary consequences of CD8^+^ T cells and Th1/2 cells in OC TME [58]. A possible explanation leading to different predictions might be that the transcriptional data enrolled in this study was in TPM form, whereas the earlier finding used the FPKM expression matrix for ssGSEA explorations. Moreover, decreased tumor-infiltrating lymphocytes (TILs) were presented in the high-risk group. This is also compatible with an earlier observation, which showed that OC patients engaged with more TILs experienced a better prognosis [59]. Thus, the treatment of OC with autologous TILs is currently being applied in several centers as an immunotherapeutic approach.

To further investigate the efficiency of immunotherapy, we conducted an analysis of immune checkpoints and TMB. Low-risk patients had increased levels of immune checkpoints such as TIGIT, CTLA-4, and LAG-3. TIGIT is usually expressed by T cells and NK cells. Data from several sources has identified that the CD155/TIGIT and DNAM-1/TIGIT/CD96 axes play important roles in OC TIGIT-based immunotherapy and overexpressed TIGIT would be a compelling indicator for promising OC treatment [60, 61]. Results from other studies revealed that high expressions of CTLA-4 and LAG-3 were associated with better survival outcomes after systemic treatment, which broadly supported the work [62]. Furthermore, TMB had a negative correlation with risk scores. Therefore, it could conceivably be assumed that low-risk OC patients might benefit from immune checkpoint blockade therapies.

However, we recognized several limitations in this study. Firstly, the data was not prospective and sufficient because it was obtained from existing public online cohorts. We did not conduct validation experiments to reveal the links between risk scores and immune fraction. The supporting information on transcriptional expression and clinicopathologic characteristics from the real world is still required. Secondly, the intrinsic weakness of merely considering a single hallmark to construct a model was inevitable since various prognostic signatures in OC have been excluded. However, based on the distinct validation to confirm the effectiveness of prognostic prediction for OC, the model was acceptable despite the weakness.

## 5. Conclusion

In summary, this study firstly established a prognostic risk model with four GRmRNAs in OC by integrating machine learning methods and statistical approaches. The prognostic risk system based on GRmRNAs could accurately predict prognosis, the immune microenvironment, and the immunotherapeutic efficacy of OC patients, where high-risk scores showed poor prognosis and low immune-infiltration levels. Glycosylation-related genes may contribute to predicting prognosis and creating personalized immunotherapies, while the regulatory mechanism of the interplay between glycosylation and tumor biology functions is worth studying further. Our model might be a valuable tool for OC risk classification, assisting clinicians to adopt the optimal therapeutic strategies for more personalized treatment in clinical practice.

## Figures and Tables

**Table 1:** Clinicopathologic information of training set and test set.

Covariates	Type	TCGA (*n* = 375)	Training (*n* = 208)	Test (*n* = 167)	*P*
Vital status	Alive	146 (38.93%)	80 (38.46%)	66 (39.52%)	0.834^a^
	Dead	229 (61.07%)	128 (61.54%)	101 (60.48%)	
Age	≤60	206 (54.93%)	105 (50.48%)	101 (60.48%)	0.053^a^
	>60	169 (45.07%)	103 (49.52%)	66 (39.52%)	
Grade	G1	1 (0.27%)	1(0.48%)	0 (0%)	0.577^b^
	G2	42 (11.2%)	20 (9.62%)	22 (13.17%)	
	G3	321 (85.6%)	181 (87.02%)	140 (83.83%)	
	G4	1 (0.27%)	1 (0.48%)	0 (0%)	
	Unknown	10 (2.67%)	5 (2.4%)	5 (2.99%)	
Stage	Stage I	1 (0.27%)	1 (0.48%)	0 (0%)	0.451^b^
	Stage II	22 (5.87%)	13 (6.25%)	9 (5.39%)	
	Stage III	292 (77.87%)	158 (75.96%)	134 (80.24%)	
	Stage IV	57 (15.2%)	33 (15.87%)	24 (14.37%)	
	Unknown	3 (0.8%)	3 (1.44%)	0 (0%)	

^a^Pearson's chi-square test; ^b^Fisher's exact test.

**Table 2:** The quantified importance of prognostic glycosylation-related messenger RNAs by machine learning.

	LASSO	Ridge	XGBoost	Random forest	AdaBoost
ALG8	0.255 (3)	0.276 (3)	95 (8)	0.108 (6)	0.088 (5)
CSNK1D	0.025 (8)	0.065 (8)	112 (5)	0.141 (1)	0.225 (1)
DCTN4	0.587 (1)	0.609 (1)	120 (3)	0.113 (5)	0.169 (2)
DCTN6	0.406 (2)	0.442 (2)	100 (7)	0.139 (2)	0.161 (3)
F8	0.108 (6)	0.124 (6)	114 (4)	0.117 (3)	0.125 (4)
FUCA1	0.113 (5)	0.134 (5)	88 (9)	0.090 (8)	0.027 (9)
NAPG	0.177 (4)	0.217 (4)	135 (2)	0.076 (9)	0.086 (6)
UBA1	0.022 (9)	0.061 (9)	148 (1)	0.101 (7)	0.069 (7)
UBB	0.089 (7)	0.092 (7)	106 (6)	0.115 (4)	0.049 (8)

The number in the parentheses represented the rankings of weight.

**Table 3:** Independent analysis of all patients.

Characteristics	Univariate			Multivariate		
	HR	95% CI	*P*	HR	95% CI	*P*
Age	1.024	1.010-1.039	0.001	1.021	1.006-1.036	0.006
Grade	1.186	0.784-2.056	0.543	1.357	0.767-2.401	0.295
Stage	1.078	0.761-1.527	0.672	1.140	0.788-1.649	0.486
Risk score	1.603	1.163-2.208	0.004	1.540	1.101-2.154	0.012

HR: hazard ratio; CI: confidence interval.

## Data Availability

Data used in this study can be downloaded from TCGA (https://tcga-data.nci.nih.gov/tcga/), GEO (https://www.ncbi.nlm.nih.gov/geo/), MSigDB (http://www.gsea-msigdb.org/gsea/msigdb/), UCSC XENA (https://xenabrowser.net/datapages/), and TIMER (http://timer.cistrome.org).

## Abbreviations

DCA: Decision curve analysis
ER: Endoplasmic reticulum
ESTIMATE: Estimation of stromal and immune cells in malignant tumors using expression data
FDA: Food and Drug Administration
FDR: False discovery rate
FPKM: Fragments per kilobase million
GEO: Gene Expression Omnibus
GO: Gene Ontology
GSEA: Gene set enrichment analysis
GSVA: Gene set variation analysis
ICB: Immune checkpoint blockade
KEGG: Kyoto Encyclopedia of Genes and Genomes
HGSOC: High-grade serous ovarian cancer
KM: Kaplan-Meier
KW: Kruskal-Wallis
LASSO: Least absolute shrinkage and selection operator
mRNAsi: mRNA expression-based stemness index
MSigDB: Molecular Signatures Database
OC: Ovarian cancer
OGRmRNA: Optimal glycosylation-related messenger RNA
PCA: Principal component analysis
RNA-seq: RNA sequencing
ROS: Reactive oxygen species
SNV: Simple nucleotide variation
ssGSEA: Single-sample gene set enrichment analysis
TCGA: The Cancer Genome Atlas
TMB: Tumor mutational burden
TME: Tumor microenvironment
TPM: Transcripts per million
tSNE: *t*-distribution stochastic neighbor embedding
WGCNA: Weighted gene coexpression network analysis.

## Supplementary Materials

Supplementary Materials Supplementary Table 1: the 636 glycosylation-related genes. Supplementary Table 2: the phenotypes of 70 OC patients for WGCNA. Supplementary Table 3: pathway signatures and genes. Supplementary Table 4: the expression of OGRmRNAs and the profiles of risk score in the training and test set. Supplementary Table 5: coexpressed genes with OGRmRNAs in ovarian cancer. Supplementary Table 6: results of GSEA by the “clusterProfiler” package. Supplementary Table 7: the expression of immune checkpoints of OC patients and their corresponding risk subtypes. Supplementary Figure 1: mRNAsi differences among low expression and high expression of OGRmRNAs and normal tissues. (A) ALG8. (B) DCTN4. (C) DCTN6. (D) UBB. Supplementary Figure 2: functional enrichment analysis with co-expressed genes of OGRmRNAs. (A) Gene Ontology terms. (B) Pathways of KEGG (Kyoto Encyclopedia of Genes and Genomes).
