# Supplementary material for: Glycosylation-Related Genes Predict the Prognosis and Immune Fraction of Ovarian Cancer Patients Based on Weighted Gene Coexpression Network Analysis (WGCNA) and Machine Learning
Source: Oxid Med Cell Longev. 2022 Mar 4;2022:3665617. doi: 10.1155/2022/3665617 (PMC8916863; doi:10.1155/2022/3665617)
Supplement: Supplementary Materials — Supplementary Table 1: the 636 glycosylation-related genes. Supplementary Table 2: the phenotypes of 70 OC patients for WGCNA. Supplementary Table 3: pathway signatures and genes. Supplementary Table 4: the expression of OGRmRNAs and the profiles of risk score in the training and test set. Supplementary Table 5: coexpressed genes with OGRmRNAs in ovarian cancer. Supplementary Table 6: results of GSEA by the “clusterProfiler” package. Supplementary Table 7: the expression of immune checkpoints of OC patients and their corresponding risk subtypes. Supplementary Figure 1: mRNAsi differences among low expression and high expression of OGRmRNAs and normal tissues. (A) ALG8. (B) DCTN4. (C) DCTN6. (D) UBB. Supplementary Figure 2: functional enrichment analysis with co-expressed genes of OGRmRNAs. (A) Gene Ontology terms. (B) Pathways of KEGG (Kyoto Encyclopedia of Genes and Genomes). [file 3665617.f1.zip › Supplementary Table 1.doc]

A4GNT

ABCA2

ACAN

ACER2

ACTR10

ACTR1A

ADAMTS1

ADAMTS10

ADAMTS12

ADAMTS13

ADAMTS14

ADAMTS15

ADAMTS16

ADAMTS17

ADAMTS18

ADAMTS19

ADAMTS2

ADAMTS20

ADAMTS3

ADAMTS4

ADAMTS5

ADAMTS6

ADAMTS7

ADAMTS8

ADAMTS9

ADAMTSL1

ADAMTSL2

ADAMTSL3

ADAMTSL4

ADAMTSL5

AGER

AGRN

ALG1

ALG10

ALG10B

ALG11

ALG12

ALG13

ALG14

ALG2

ALG3

ALG5

ALG6

ALG8

ALG9

AMDHD2

AMFR

ANK1

ANK2

ANK3

ANKRD28

APOB

APP

ARCN1

AREG

ARF1

ARF3

ARF4

ARF5

ARFGAP1

ARFGAP2

ARFGAP3

ARFGEF1

ARL6IP1

ARSB

ASGR1

ASGR2

ATP6AP2

ATP6V0A2

ATP6V1A

ATP6V1E1

B3GALNT2

B3GALT1

B3GALT2

B3GALT6

B3GAT3

B3GLCT

B3GNT2

B3GNT3

B3GNT4

B3GNT5

B3GNT6

B3GNT7

B3GNT8

B3GNT9

B3GNTL1

B4GALNT2

B4GALT1

B4GALT2

B4GALT3

B4GALT4

B4GALT5

B4GALT6

B4GALT7

B4GAT1

BCAN

BET1

BET1L

BGLAP

BGN

C1GALT1

C1GALT1C1

C1GALT1C1L

CA4

CAD

CALR

CANX

CAPZA1

CAPZA2

CAPZA3

CAPZB

CASQ1

CCDC115

CCDC47

CD55

CD59

CDKAL1

CFP

CGA

CHP1

CHST10

CHST14

CHST3

CHST4

CHST6

CHST8

CHSY1

CKAP4

CMAS

CNIH1

CNIH2

CNIH3

COG1

COG2

COG3

COG4

COG5

COG6

COG7

COG8

COL7A1

COPA

COPB1

COPB2

COPE

COPG1

COPG2

COPZ1

COPZ2

CRPPA

CSNK1D

CSPG4

CSPG5

CTSA

CTSC

CTSZ

CYBB

DAD1

DAG1

DCN

DCTN1

DCTN2

DCTN3

DCTN4

DCTN5

DCTN6

DDOST

DERL1

DERL2

DERL3

DHDDS

DNAJC3

DOLK

DOLPP1

DPAGT1

DPM1

DPM2

DPM3

DPY19L1

DPY19L2

DPY19L3

DPY19L4

DYNC1H1

DYNC1I1

DYNC1I2

DYNC1LI1

DYNC1LI2

DYNLL1

DYNLL2

EDEM1

EDEM2

EDEM3

EDN1

ENGASE

ENTPD5

EOGT

EPHA5

ERP29

EXT1

EXT2

F12

F5

F8

FCSK

FKRP

FKTN

FMOD

FOLR1

FPGT

FTCD

FUCA1

FUOM

FUT3

FUT4

FUT5

FUT6

FUT7

FUT8

FUT9

GABARAP

GAL3ST1

GALE

GALK1

GALNT1

GALNT10

GALNT11

GALNT12

GALNT13

GALNT14

GALNT15

GALNT16

GALNT17

GALNT18

GALNT2

GALNT3

GALNT4

GALNT5

GALNT6

GALNT7

GALNT8

GALNT9

GALNTL5

GALNTL6

GALT

GANAB

GBA2

GBF1

GCC1

GCNT1

GCNT3

GCNT4

GCNT7

GFPT1

GFPT2

GFUS

GLB1

GMDS

GMPPA

GMPPB

GNE

GNPNAT1

GNRH1

GOLGA2

GOLGB1

GORASP1

GOSR1

GOSR2

GPC1

GPC2

GPC3

GPC4

GPC5

GPC6

GRIA1

GXYLT1

GXYLT2

HCRT

HEXA

HEXB

HGFAC

HID1

HM13

HMGB1

HSD3B1

HSD3B2

HSPA5

HSPG2

HTR5A

HYOU1

IL15

INS

KCNJ2

KDELR1

KDELR2

KDELR3

KERA

KRTCAP2

LARGE1

LARGE2

LFNG

LGALS3

LHB

LIN28A

LMAN1

LMAN1L

LMAN2

LMAN2L

LRAT

LRPAP1

LUM

MACO1

MAGT1

MAN1A1

MAN1A2

MAN1B1

MAN1C1

MAN2A1

MAN2A2

MAN2B1

MAN2C1

MANEA

MAPK1

MAPK3

MARCHF6

MCFD2

MGAT1

MGAT2

MGAT3

MGAT4A

MGAT4B

MGAT4C

MGAT4D

MGAT5

MGAT5B

MIA2

MIA3

MLEC

MOGS

MPDU1

MPI

MT3

MTTP

MUC1

MUC12

MUC13

MUC15

MUC16

MUC17

MUC19

MUC2

MUC20

MUC21

MUC3A

MUC4

MUC5AC

MUC5B

MUC6

MUC7

MUCL1

MVD

MYO1D

MYOC

NAGK

NANP

NANS

NAPA

NAPB

NAPG

NAT8L

NCAN

NCF1

NEU1

NEU2

NEU3

NEU4

NGLY1

NOTCH1

NOTCH2

NOTCH3

NOTCH4

NPL

NSF

NUCB1

NUDT14

NUS1

OGA

OGN

OGT

OMD

OS9

OST4

OSTC

P2RX3

PAPSS2

PCSK9

PDIA3

PGM1

PGM3

PGRMC1

PI4KB

PKM

PLCB4

PLOD1

PLOD2

PLOD3

PMM1

PMM2

POFUT1

POFUT2

POGLUT1

POGLUT2

POGLUT3

POMGNT1

POMGNT2

POMK

POMT1

POMT2

PPP6C

PPP6R1

PPP6R3

PRDX4

PREB

PRELP

PRKCSH

PSEN1

PSMC1

PTGDS

RAB14

RAB1A

RAB1B

RAD23B

RAMP1

RANGRF

RENBP

RFT1

RNF103

RNF139

RNF185

RNF5

RP9

RPL10

RPL18

RPL27

RPL4

RPL6

RPN1

RPN2

RPS21

RPS23

RPS26

RPS27A

RPS28

RPS29

RXYLT1

RYR1

RYR2

RYR3

S100A12

S100B

SAA1

SAR1B

SBSPON

SCFD1

SCGB1A1

SDC1

SDC2

SDC3

SDC4

SDF2L1

SEC13

SEC16A

SEC16B

SEC22A

SEC22B

SEC22C

SEC23A

SEC23IP

SEC24A

SEC24B

SEC24C

SEC24D

SEC31A

SEC61A1

SEC61A2

SEC61B

SEC61G

SEC62

SEC63

SEL1L

SEMA5A

SEMA5B

SERPINA1

SFTPA1

SFTPA2

SFTPD

SGMS1

SLC17A5

SLC26A2

SLC35A1

SLC35A2

SLC35C1

SLC35C2

SLC39A8

SLC51B

SLC7A11

SLMAP

SPON1

SPON2

SPPL3

SPTA1

SPTAN1

SPTB

SPTBN1

SPTBN2

SPTBN4

SPTBN5

SRD5A3

SRI

SRP9

SRPRA

SRPRB

SSPOP

SSR4

ST3GAL1

ST3GAL2

ST3GAL3

ST3GAL4

ST3GAL5

ST3GAL6

ST6GAL1

ST6GAL2

ST6GALNAC1

ST6GALNAC2

ST6GALNAC3

ST6GALNAC4

ST6GALNAC5

ST6GALNAC6

ST8SIA1

ST8SIA2

ST8SIA3

ST8SIA4

ST8SIA5

ST8SIA6

STAU1

STT3A

STT3B

STX17

STX5

SUCO

SVIP

SYNE3

SYVN1

TBC1D20

TET1

TET2

TET3

TFG

TGFA

TH

THBS1

THBS2

THSD1

THSD4

THSD7A

THSD7B

TMCC1

TMED10

TMED2

TMED3

TMED7

TMED9

TMEM115

TMEM165

TMEM199

TMEM258

TMEM59

TMEM97

TMTC1

TMTC2

TMTC3

TMTC4

TP63

TRAK1

TRAK2

TRAPPC1

TRAPPC10

TRAPPC2

TRAPPC2L

TRAPPC3

TRAPPC4

TRAPPC5

TRAPPC6A

TRAPPC6B

TRAPPC9

TRIM13

TTYH1

TUBA1A

TUBA1B

TUBA1C

TUBA3C

TUBA3D

TUBA3E

TUBA4A

TUBA4B

TUBA8

TUBAL3

TUBB1

TUBB2A

TUBB2B

TUBB3

TUBB4A

TUBB4B

TUBB6

TUBB8

TUBB8B

TUSC3

UAP1

UBA1

UBA52

UBB

UBC

UBE2J1

UBXN1

UGGT1

UGGT2

UMOD

USO1

VCAN

VCP

VEGFB

XXYLT1

YIPF1

YIPF2

YIPF6

YKT6

ZC3H12A
