# Supplementary material for: Glycosylation-Related Genes Predict the Prognosis and Immune Fraction of Ovarian Cancer Patients Based on Weighted Gene Coexpression Network Analysis (WGCNA) and Machine Learning
Source: Oxid Med Cell Longev. 2022 Mar 4;2022:3665617. doi: 10.1155/2022/3665617 (PMC8916863; doi:10.1155/2022/3665617)
Supplement: Supplementary Materials — Supplementary Table 1: the 636 glycosylation-related genes. Supplementary Table 2: the phenotypes of 70 OC patients for WGCNA. Supplementary Table 3: pathway signatures and genes. Supplementary Table 4: the expression of OGRmRNAs and the profiles of risk score in the training and test set. Supplementary Table 5: coexpressed genes with OGRmRNAs in ovarian cancer. Supplementary Table 6: results of GSEA by the “clusterProfiler” package. Supplementary Table 7: the expression of immune checkpoints of OC patients and their corresponding risk subtypes. Supplementary Figure 1: mRNAsi differences among low expression and high expression of OGRmRNAs and normal tissues. (A) ALG8. (B) DCTN4. (C) DCTN6. (D) UBB. Supplementary Figure 2: functional enrichment analysis with co-expressed genes of OGRmRNAs. (A) Gene Ontology terms. (B) Pathways of KEGG (Kyoto Encyclopedia of Genes and Genomes). [file 3665617.f1.zip › Supplementary Table 2.doc]

| id | Follow-up time | Vital status | Grade | Figo stage | Tumor size | Clinical status |
| --- | --- | --- | --- | --- | --- | --- |
| GSM1559299 | 104 | 1 | G4 | IIIC | R2 | P |
| GSM1559300 | 286 | 1 | G4 | IIIC | R2 | P |
| GSM1559301 | 357 | 1 | G3 | IV | R1 | P |
| GSM1559302 | 385 | 1 | G3 | IIIC | R1 | P |
| GSM1559303 | 414 | 1 | G2 | IIIC | R1 | P |
| GSM1559304 | 416 | 1 | G3 | IIIC | R1 | PR |
| GSM1559305 | 434 | 1 | G3 | IIIC | R1 | PR |
| GSM1559306 | 479 | 1 | G3 | IV | R1 | SD |
| GSM1559307 | 498 | 1 | G3 | IIIA | R0 | PR |
| GSM1559308 | 505 | 1 | G3 | IIIC | R1 | PR |
| GSM1559309 | 508 | 1 | G3 | IIIC | R2 | P |
| GSM1559310 | 510 | 1 | G3 | IIIC | R1 | PR |
| GSM1559311 | 632 | 1 | G4 | IIIC | R1 | PR |
| GSM1559312 | 735 | 1 | G3 | IIIC | R1 | PR |
| GSM1559313 | 761 | 1 | G4 | IIIC | R1 | PR |
| GSM1559314 | 786 | 1 | G3 | IIIC | R2 | PR |
| GSM1559315 | 790 | 1 | G4 | IIIC | R2 | P |
| GSM1559316 | 930 | 0 | G3 | IIIC | R1 | PR |
| GSM1559317 | 1105 | 1 | G3 | IV | R1 | PR |
| GSM1559318 | 1111 | 1 | G4 | IIIC | R2 | PR |
| GSM1559319 | 1124 | 1 | G4 | IV | R0 | PR |
| GSM1559320 | 1138 | 1 | G3 | IV | R2 | SD |
| GSM1559321 | 1548 | 1 | G4 | IIIB | R0 | PR |
| GSM1559322 | 1751 | 1 | G3 | IIIC | R1 | PR |
| GSM1559323 | 1874 | 1 | G2 | IIIC | R1 | SD |
| GSM1559324 | 877 | 1 | G3 | IIIC | R1 | CR |
| GSM1559325 | 443 | 1 | G3 | IIIC | R2 | CR |
| GSM1559326 | 701 | 1 | G3 | IIIC | R1 | CR |
| GSM1559327 | 1318 | 1 | G3 | IIIC | R2 | CR |
| GSM1559328 | 1573 | 1 | G4 | IIIC | R0 | CR |
| GSM1559329 | 785 | 1 | G4 | IIIC | R2 | CR |
| GSM1559330 | 1203 | 1 | G3 | IIIB | R0 | CR |
| GSM1559331 | 687 | 1 | G3 | IV | R2 | CR |
| GSM1559332 | 778 | 1 | G3 | IIIC | R1 | CR |
| GSM1559333 | 905 | 1 | G3 | IIIC | R1 | CR |
| GSM1559334 | 1014 | 1 | G4 | IV | R1 | CR |
| GSM1559335 | 373 | 1 | G3 | IIIC | R2 | CR |
| GSM1559336 | 652 | 1 | G3 | IIIC | R2 | CR |
| GSM1559337 | 467 | 1 | G2 | IV | R1 | CR |
| GSM1559338 | 1168 | 1 | G3 | IV | R1 | CR |
| GSM1559339 | 1196 | 1 | G3 | IIIC | R1 | CR |
| GSM1559340 | 679 | 1 | G4 | IIIC | R2 | CR |
| GSM1559341 | 1357 | 1 | G3 | IV | R1 | CR |
| GSM1559342 | 860 | 1 | G4 | IIIC | R0 | CR |
| GSM1559343 | 1005 | 1 | G3 | IIIC | R1 | CR |
| GSM1559344 | 1256 | 1 | G3 | IIIC | R2 | CR |
| GSM1559345 | 897 | 1 | G4 | IIIC | R2 | CR |
| GSM1559346 | 1193 | 1 | G4 | IIIA | R1 | CR |
| GSM1559347 | 1066 | 1 | G3 | IIIC | R1 | CR |
| GSM1559348 | 677 | 1 | G3 | IIIB | R1 | CR |
| GSM1559349 | 1373 | 1 | G3 | IIIC | R2 | CR |
| GSM1559350 | 1299 | 0 | G4 | IIIC | R2 | CR |
| GSM1559351 | 1780 | 1 | G2 | IIC | R1 | CR |
| GSM1559354 | 1955 | 1 | G2 | IIIB | R0 | CR |
| GSM1559355 | 1092 | 1 | G3 | IIIA | R1 | CR |
| GSM1559356 | 2351 | 1 | G3 | IIIB | R0 | CR |
| GSM1559357 | 1600 | 1 | G3 | IIIB | R0 | CR |
| GSM1559358 | 4080 | 0 | G2 | IIIC | R0 | CR |
| GSM1559359 | 1583 | 1 | G3 | IIIC | R1 | CR |
| GSM1559360 | 2079 | 1 | G4 | IIIC | R1 | CR |
| GSM1559361 | 1255 | 1 | G3 | IIIC | R1 | CR |
| GSM1559362 | 2592 | 0 | G3 | IIIA | R0 | CR |
| GSM1559363 | 1253 | 1 | G4 | IIIA | R1 | CR |
| GSM1559364 | 2320 | 1 | G2 | IIIC | R1 | CR |
| GSM1559365 | 1997 | 1 | G2 | IIIC | R0 | CR |
| GSM1559366 | 1849 | 1 | G3 | IIIC | R2 | CR |
| GSM1559367 | 1847 | 1 | G3 | IIIC | R1 | CR |
| GSM1559368 | 2346 | 1 | G3 | IIIC | R0 | CR |
| GSM1559369 | 3750 | 1 | G3 | IIIB | R0 | CR |
| GSM1559370 | 2797 | 1 | G3 | IIIC | R2 | CR |
