# Supplementary material for: Glycosylation-Related Genes Predict the Prognosis and Immune Fraction of Ovarian Cancer Patients Based on Weighted Gene Coexpression Network Analysis (WGCNA) and Machine Learning
Source: Oxid Med Cell Longev. 2022 Mar 4;2022:3665617. doi: 10.1155/2022/3665617 (PMC8916863; doi:10.1155/2022/3665617)
Supplement: Supplementary Materials — Supplementary Table 1: the 636 glycosylation-related genes. Supplementary Table 2: the phenotypes of 70 OC patients for WGCNA. Supplementary Table 3: pathway signatures and genes. Supplementary Table 4: the expression of OGRmRNAs and the profiles of risk score in the training and test set. Supplementary Table 5: coexpressed genes with OGRmRNAs in ovarian cancer. Supplementary Table 6: results of GSEA by the “clusterProfiler” package. Supplementary Table 7: the expression of immune checkpoints of OC patients and their corresponding risk subtypes. Supplementary Figure 1: mRNAsi differences among low expression and high expression of OGRmRNAs and normal tissues. (A) ALG8. (B) DCTN4. (C) DCTN6. (D) UBB. Supplementary Figure 2: functional enrichment analysis with co-expressed genes of OGRmRNAs. (A) Gene Ontology terms. (B) Pathways of KEGG (Kyoto Encyclopedia of Genes and Genomes). [file 3665617.f1.zip › Supplementary Table 3.doc]

| Signature | Genes |
| --- | --- |
| PI3K/Akt | AKT1, AKT2, AKT3, BAD, BCL2L1, CASP9, CDKN1A, CDKN1B, CHUK, FOXO1, FOXO3, FOXO4, GSK3B, HSP90AA1, KPNA1, MAP3K5, MAPKAP1, MLST8, MTOR, PDPK1, PRKACA, PRKDC, RAF1, RICTOR, SFN, SLC2A4, TBC1D4, YWHAB, YWHAE, YWHAG, YEHAH, YWHAQ, YWHAZ |
| EMT | CDH10, EDN2, PAPPA, PCDH9, WNT2, ANLN, APLP2, CD63, CDH2, CLIC4, CTSB, CX3CR1, DSG2, EDNRB, EMP1, ENC1, FGG, FZD1, HMMR, ITGAV, L1CAM, LUM, MFAP1, MMP2, PFN1, PRKCA, RAB1A, RAN, RRAGA, SDCBP, SELENOP, SERPINA3, SMARCA1, SPA17, SPARC, TUBA1A, TUBA3C, TUBA4A, TUBB3, VCAN |
| Notch | APH1A, ARRB1, CCND1, CUL1, DLL1, DTX1, DTX2, DTX4, FBXW11, FZD1, FZD5, FZD7, HES1, HEYL, JAG1, KAT2A, LFNG, MAML2, NOTCH1, NOTCH2, NOTCH3, PPARD, PRKCA, PSEN2, PSENEN, RBX1, SAP30, SKP1, ST3GAL6, WNT2, WNT5A |
| Hedgehog | ACHE, ADGRG1, AMOT, CDK5R1, CDK6, CELSR1, CNTFG, CRMP1, DPYSL2, ETS2, GLI1, HEY1, HEY2, L1CAM, LDB1, MYH9, NF1, NKX6-1, NRCAM, NRP1, NRP2, OPHN1, PLG, PML, PTCH1, RASA1, RTN1, SCG2, SHH, SLIT1, THY1, TLE1, TLE3, UNC5C, VEGFA, VLDLR |
| JAK_STAT | CSH1, GH1, GH2, GHR, JAK2, JAK3, LYN, PRL, PRLR, PTPN1, STAT3, STAT5A, STAT5B, STAT6 |
| NF_kappa_B | EGF, MMP2, MMP9, MT-CO2, PLAU, PTGS2, SPP1, TGFA, VEGFA, VEGFB, VEGFC |
| TGFβ | APC, CDH1, CREBBP, EP300, MAP2K1, MAP3K7, MAPK3, SKIL, SMAD2, SMAD3, SMAD4, SMAD7, TAB1, TGFB1, TGFB2, TGFB3, TGFBR1, TGFBR2, ZFYVE9 |
| Interleukin-8 | ANXA1, ANXA4, ARRB1, BCL3, BPI, CACTIN, ELANE, IL10, KLF4, MAP2K5, MAPKBP1, MIR100, MIR106A, MIR106B, MIR129-1, MIR132, MIR146A, MIR181A2, MIR203A, MIR204, MIR302C,MIR203D, MIR488, MIR506, MIR520C, MIR766, MIR920, MIR93, MIR98, MIRLET7C, OTUD7B, PTPN22, SC5D, TLR6, TLR9, TMSB4X |
| Wnt | APC, AXIN1, BTRC, CCND1, CSNK2A1, CTBP1, CTNNB1, DVL1, FRAT1, FZD1, GSK3B, HDAC1, HNF1A, MAP3K7, MYC, NLK, PPARD, PPP2CA, SMAD4, TAB1, TLE1, WIF1, WNT1 |
| Interferon | ADAR, APOBEC3G, BRD3, BST2, CMTR1, EIF2AK2, ERG, IFI30, IFI35, IFI44, IFI44L, IFI6, IFIT1, IFIT3, IRF9, ISG15, LY6E, MORC3, MX1, OAS1, OAS2, RXRA, SP100, SP110, STAT1, TGIF1, UBE2L6 |
| Hippo | AJUBA, AMOT, AMOTL1, AMOTL2, CASP3, CIT, DCHS1, DLG5, DVL2, FAT4, FRMD1, IQCJ-SCHIP1, LATS1, LATS2, LIMD1, MAP2K3, MAPK14, MARK3, MOB1A, MOB1B, MOB3B, NEK8, NPHP4, PJA2, SAV1, SCHIP1, SHANK2, SOX11, STK3, STK4, TEAD1, TEAD2, TEAD3, TEAD4, TJP1, TJP2, VGLL4, WTIP, WWC1, WWC2, WWC3, WWTR1, YAP1, YWHAB, YWHAE |
| ROS | AKR7A2, CREB1, CRYZ, FOS, FXYD2, GSTA2, HMOX1, HMOX2, JUN, KEAP1, MAFF, MAFG, MAFK, MAPK1, MAPK14, NEF2L2, POR, PRKCA, PRKCB |
