# Supplementary material for: Glycosylation-Related Genes Predict the Prognosis and Immune Fraction of Ovarian Cancer Patients Based on Weighted Gene Coexpression Network Analysis (WGCNA) and Machine Learning
Source: Oxid Med Cell Longev. 2022 Mar 4;2022:3665617. doi: 10.1155/2022/3665617 (PMC8916863; doi:10.1155/2022/3665617)
Supplement: Supplementary Materials — Supplementary Table 1: the 636 glycosylation-related genes. Supplementary Table 2: the phenotypes of 70 OC patients for WGCNA. Supplementary Table 3: pathway signatures and genes. Supplementary Table 4: the expression of OGRmRNAs and the profiles of risk score in the training and test set. Supplementary Table 5: coexpressed genes with OGRmRNAs in ovarian cancer. Supplementary Table 6: results of GSEA by the “clusterProfiler” package. Supplementary Table 7: the expression of immune checkpoints of OC patients and their corresponding risk subtypes. Supplementary Figure 1: mRNAsi differences among low expression and high expression of OGRmRNAs and normal tissues. (A) ALG8. (B) DCTN4. (C) DCTN6. (D) UBB. Supplementary Figure 2: functional enrichment analysis with co-expressed genes of OGRmRNAs. (A) Gene Ontology terms. (B) Pathways of KEGG (Kyoto Encyclopedia of Genes and Genomes). [file 3665617.f1.zip › Supplementary Table 4.doc]

| id | time | event | ALG8 | DCTN4 | DCTN6 | UBB | RiskScore | Risk |
| --- | --- | --- | --- | --- | --- | --- | --- | --- |
| TCGA-25-1313 | 2.246575 | 1 | 6.149259 | 4.699795 | 4.750578 | 9.557665 | 0.144863 | low |
| TCGA-61-2000 | 1.208219 | 0 | 5.461487 | 5.645763 | 4.825942 | 9.638942 | 0.174563 | high |
| TCGA-23-1027 | 2.673973 | 1 | 6.381167 | 4.185365 | 4.790408 | 9.689544 | 0.129222 | low |
| TCGA-24-1546 | 5.356164 | 1 | 5.122029 | 4.708141 | 4.752434 | 9.160585 | 0.172383 | high |
| TCGA-13-0795 | 1.69589 | 1 | 3.970536 | 4.268063 | 4.514654 | 7.590743 | 0.231345 | high |
| TCGA-09-0366 | 4.813699 | 1 | 8.770041 | 4.133864 | 5.430926 | 8.466115 | 0.097679 | low |
| TCGA-61-2002 | 1.49863 | 0 | 6.502691 | 4.950201 | 5.619112 | 9.696973 | 0.123308 | low |
| TCGA-30-1853 | 3.021918 | 1 | 5.144329 | 4.592508 | 5.485332 | 3.887099 | 0.264655 | high |
| TCGA-13-0886 | 12.78082 | 0 | 5.264297 | 4.0958 | 5.372424 | 8.762515 | 0.148719 | low |
| TCGA-09-0369 | 2.964384 | 1 | 5.605693 | 4.364923 | 6.674198 | 6.843876 | 0.147885 | low |
| TCGA-23-1022 | 4.139726 | 1 | 6.491506 | 3.510272 | 4.716963 | 9.585515 | 0.119688 | low |
| TCGA-04-1542 | 7.016438 | 1 | 5.973754 | 3.594273 | 5.398414 | 9.752471 | 0.114408 | low |
| TCGA-24-2035 | 2.347945 | 1 | 5.702085 | 3.680323 | 4.686262 | 9.046014 | 0.143856 | low |
| TCGA-24-2027 | 9.142466 | 1 | 7.206613 | 4.80144 | 4.507734 | 9.963861 | 0.127443 | low |
| TCGA-04-1648 | 2.386301 | 1 | 5.065727 | 3.954112 | 5.461176 | 5.540573 | 0.207889 | high |
| TCGA-20-1683 | 2.115068 | 0 | 5.866318 | 5.609129 | 5.061059 | 6.111642 | 0.231213 | high |
| TCGA-24-1557 | 3.323288 | 1 | 6.1466 | 4.269153 | 5.573804 | 5.957196 | 0.1772 | high |
| TCGA-25-2399 | 1.665753 | 1 | 5.510961 | 5.091058 | 4.953831 | 7.727392 | 0.194231 | high |
| TCGA-23-2078 | 7.290411 | 0 | 5.908784 | 5.778927 | 5.013262 | 9.498194 | 0.165369 | low |
| TCGA-29-1691 | 4.027397 | 1 | 6.089733 | 4.690153 | 5.468827 | 6.34629 | 0.183512 | high |
| TCGA-29-1688 | 6.575342 | 1 | 4.983291 | 4.241692 | 5.470369 | 8.390498 | 0.160881 | low |
| TCGA-13-1477 | 4.553425 | 1 | 4.876394 | 3.541365 | 5.322017 | 5.838238 | 0.200157 | high |
| TCGA-13-0905 | 10.26301 | 0 | 6.110735 | 4.449309 | 6.179117 | 5.033746 | 0.183045 | high |
| TCGA-24-1924 | 2.517808 | 1 | 6.007563 | 4.725127 | 4.38301 | 10.38346 | 0.143427 | low |
| TCGA-31-1953 | 0.558904 | 0 | 4.793635 | 5.100447 | 5.523971 | 8.514829 | 0.179717 | high |
| TCGA-29-1693 | 8.482192 | 0 | 5.965694 | 5.138085 | 6.239469 | 9.582564 | 0.124484 | low |
| TCGA-61-2101 | 4.624658 | 1 | 5.423616 | 4.572837 | 4.201655 | 9.118365 | 0.178186 | high |
| TCGA-23-1029 | 0.734247 | 0 | 6.484501 | 5.384347 | 4.670747 | 8.830261 | 0.16536 | low |
| TCGA-24-1843 | 0.290411 | 0 | 5.249485 | 4.642975 | 5.409599 | 9.547573 | 0.146045 | low |
| TCGA-13-1495 | 7.531507 | 1 | 4.96906 | 4.733138 | 4.676074 | 5.636666 | 0.259033 | high |
| TCGA-31-1951 | 1.873973 | 0 | 5.244592 | 5.202541 | 4.518695 | 9.181469 | 0.186729 | high |
| TCGA-23-1123 | 2.789041 | 1 | 5.524982 | 3.970435 | 5.70525 | 9.713872 | 0.121695 | low |
| TCGA-13-1501 | 3.6 | 1 | 6.007824 | 3.59303 | 4.702254 | 9.851965 | 0.125347 | low |
| TCGA-13-1488 | 5.90137 | 1 | 5.144655 | 4.197475 | 5.849929 | 9.176042 | 0.136101 | low |
| TCGA-29-1711 | 2.884932 | 0 | 5.09783 | 4.619633 | 4.877518 | 9.103503 | 0.168785 | high |
| TCGA-13-1489 | 6.994521 | 1 | 6.668951 | 4.385571 | 5.022963 | 10.17503 | 0.1171 | low |
| TCGA-29-1763 | 5.567123 | 0 | 4.991881 | 5.566555 | 5.865717 | 10.16637 | 0.148063 | low |
| TCGA-13-1404 | 6.764384 | 0 | 6.426103 | 4.536285 | 4.573201 | 9.809722 | 0.137048 | low |
| TCGA-29-1762 | 7.216438 | 1 | 5.638282 | 4.634043 | 5.075718 | 9.960103 | 0.139841 | low |
| TCGA-20-0991 | 2.183562 | 0 | 4.885909 | 2.969746 | 4.446711 | 9.907995 | 0.138262 | low |
| TCGA-04-1536 | 2.424658 | 1 | 4.800608 | 3.958097 | 4.210394 | 5.31146 | 0.266848 | high |
| TCGA-61-1910 | 3.087671 | 0 | 4.56048 | 5.380082 | 4.668788 | 5.686243 | 0.294782 | high |
| TCGA-24-0979 | 3.463014 | 1 | 5.48306 | 5.158755 | 4.109972 | 6.741698 | 0.248224 | high |
| TCGA-20-0987 | 1.920548 | 1 | 4.50154 | 3.527643 | 4.503239 | 10.24422 | 0.149019 | low |
| TCGA-29-1766 | 3.284932 | 1 | 5.510707 | 4.159645 | 5.688563 | 8.108533 | 0.148343 | low |
| TCGA-61-1900 | 0.482192 | 0 | 5.184982 | 5.264598 | 5.236076 | 9.490365 | 0.164479 | low |
| TCGA-24-1427 | 0.40274 | 0 | 5.255072 | 4.733206 | 4.99726 | 7.494517 | 0.195348 | high |
| TCGA-61-2102 | 0.539726 | 1 | 4.6527 | 5.86899 | 4.321697 | 6.459577 | 0.300983 | high |
| TCGA-24-2280 | 5.871233 | 0 | 4.519943 | 5.446611 | 4.476782 | 8.441164 | 0.229883 | high |
| TCGA-61-1998 | 0.460274 | 0 | 5.698316 | 4.644966 | 4.996004 | 9.566954 | 0.146642 | low |
| TCGA-29-1784 | 0.446575 | 0 | 5.246371 | 4.59787 | 3.888971 | 10.40435 | 0.167383 | high |
| TCGA-24-2038 | 3.709589 | 1 | 4.956434 | 4.523481 | 5.02547 | 6.741332 | 0.213143 | high |
| TCGA-23-1107 | 0.024658 | 1 | 5.424204 | 4.173343 | 6.883931 | 10.40349 | 0.098182 | low |
| TCGA-30-1718 | 4.326027 | 1 | 5.363203 | 4.720402 | 5.202896 | 9.970766 | 0.14344 | low |
| TCGA-36-1570 | 1.794521 | 0 | 6.313456 | 5.919981 | 4.787337 | 8.443937 | 0.18499 | high |
| TCGA-61-1738 | 2.983562 | 1 | 5.628171 | 4.750461 | 5.469489 | 10.05496 | 0.132453 | low |
| TCGA-13-1496 | 0.353425 | 1 | 4.883044 | 3.322709 | 4.720258 | 8.592383 | 0.159378 | low |
| TCGA-24-2262 | 0.030137 | 1 | 7.126232 | 4.884975 | 5.819586 | 9.555005 | 0.111195 | low |
| TCGA-24-1103 | 4.509589 | 1 | 6.089762 | 4.44198 | 5.980922 | 8.887356 | 0.12571 | low |
| TCGA-61-1914 | 4.717808 | 0 | 5.552248 | 5.006077 | 5.222995 | 9.900996 | 0.145762 | low |
| TCGA-09-1659 | 0.832877 | 1 | 4.816893 | 3.978116 | 5.932019 | 10.70279 | 0.116 | low |
| TCGA-25-1623 | 1.547945 | 1 | 5.141735 | 4.668849 | 5.563488 | 8.923647 | 0.155002 | low |
| TCGA-24-1930 | 6.758904 | 1 | 5.026185 | 5.052945 | 3.969772 | 9.803539 | 0.191864 | high |
| TCGA-61-1741 | 2.805479 | 1 | 5.388912 | 5.27264 | 4.811646 | 6.282135 | 0.240349 | high |
| TCGA-10-0936 | 3.076712 | 1 | 4.985473 | 3.901812 | 4.561635 | 5.488043 | 0.240727 | high |
| TCGA-13-1408 | 4.60274 | 1 | 4.812761 | 4.491835 | 4.190554 | 9.138829 | 0.1906 | high |
| TCGA-13-0906 | 9.915068 | 0 | 6.128085 | 3.804321 | 5.718578 | 9.501713 | 0.112626 | low |
| TCGA-29-1761 | 1.446575 | 1 | 4.45435 | 4.699637 | 4.218719 | 9.860859 | 0.18892 | high |
| TCGA-24-1847 | 0.939726 | 0 | 5.89353 | 4.591301 | 5.14108 | 9.72129 | 0.136707 | low |
| TCGA-09-2053 | 3.312329 | 0 | 4.919352 | 4.601793 | 5.013636 | 7.199977 | 0.206398 | high |
| TCGA-29-1694 | 3.252055 | 1 | 5.496456 | 5.376236 | 4.783606 | 6.924613 | 0.225353 | high |
| TCGA-24-1417 | 0.652055 | 0 | 5.536026 | 5.973322 | 5.335173 | 10.02743 | 0.159973 | low |
| TCGA-61-2110 | 3.709589 | 1 | 5.268955 | 4.604821 | 4.171578 | 9.36376 | 0.178613 | high |
| TCGA-25-1329 | 1.252055 | 1 | 4.768301 | 5.045205 | 5.413954 | 6.477803 | 0.225889 | high |
| TCGA-09-2051 | 5.257534 | 0 | 6.322115 | 5.418099 | 5.297522 | 11.14527 | 0.120587 | low |
| TCGA-13-0726 | 2.6 | 1 | 5.150114 | 4.707102 | 6.44873 | 9.356566 | 0.129817 | low |
| TCGA-30-1857 | 0.021918 | 1 | 5.334133 | 3.863671 | 4.915477 | 8.892689 | 0.151415 | low |
| TCGA-13-1507 | 5.460274 | 1 | 4.904514 | 4.011296 | 5.049236 | 10.52318 | 0.134349 | low |
| TCGA-57-1582 | 2.00274 | 1 | 5.262413 | 5.054984 | 5.334806 | 6.262493 | 0.219876 | high |
| TCGA-24-1845 | 0.317808 | 0 | 5.136425 | 3.807693 | 4.349113 | 8.590747 | 0.173592 | high |
| TCGA-59-2348 | 15.01644 | 0 | 5.764281 | 4.773263 | 4.653211 | 9.899672 | 0.150332 | low |
| TCGA-13-0884 | 8.931507 | 1 | 4.779684 | 3.522913 | 5.383233 | 8.125117 | 0.157241 | low |
| TCGA-57-1583 | 0.947945 | 1 | 6.049945 | 4.200662 | 3.862496 | 5.497308 | 0.242528 | high |
| TCGA-24-1846 | 0.364384 | 0 | 5.711669 | 4.934083 | 4.899884 | 6.620557 | 0.210413 | high |
| TCGA-29-1690 | 3.967123 | 1 | 5.060783 | 5.026394 | 6.145436 | 7.33391 | 0.177328 | high |
| TCGA-24-1423 | 0.520548 | 0 | 5.91754 | 4.077695 | 4.878034 | 8.828813 | 0.146188 | low |
| TCGA-13-0900 | 11.16986 | 0 | 5.392588 | 4.569503 | 6.159743 | 7.465777 | 0.157925 | low |
| TCGA-29-2425 | 5.416438 | 0 | 5.674397 | 4.856939 | 5.508843 | 6.716867 | 0.188845 | high |
| TCGA-24-1552 | 3.449315 | 1 | 5.621468 | 4.311947 | 4.971707 | 6.850704 | 0.19002 | high |
| TCGA-09-2048 | 0.378082 | 1 | 7.380606 | 5.246004 | 6.139114 | 11.00897 | 0.091971 | low |
| TCGA-31-1946 | 2.515068 | 0 | 6.253181 | 5.539059 | 4.6887 | 9.818926 | 0.15598 | low |
| TCGA-24-1416 | 0.531507 | 0 | 5.35372 | 4.202975 | 4.394802 | 9.554073 | 0.159125 | low |
| TCGA-10-0931 | 2.739726 | 1 | 6.404751 | 3.931335 | 4.678037 | 10.66324 | 0.11452 | low |
| TCGA-36-1577 | 2.145205 | 0 | 5.862876 | 4.87724 | 4.679369 | 9.538155 | 0.155645 | low |
| TCGA-25-1627 | 1.079452 | 1 | 7.234543 | 5.265371 | 5.518957 | 6.330163 | 0.169424 | high |
| TCGA-24-2281 | 3.717808 | 0 | 7.036293 | 3.948778 | 4.451421 | 8.402423 | 0.139235 | low |
| TCGA-13-0724 | 0.227397 | 1 | 4.618775 | 3.926162 | 4.421206 | 9.449173 | 0.170004 | high |
| TCGA-36-1571 | 1.90411 | 1 | 5.400501 | 5.418796 | 4.540311 | 5.270942 | 0.28356 | high |
| TCGA-24-1426 | 0.446575 | 0 | 4.463092 | 4.429158 | 5.316738 | 8.944698 | 0.16994 | high |
| TCGA-24-1603 | 7.512329 | 1 | 5.597185 | 5.296613 | 5.726498 | 9.893923 | 0.139289 | low |
| TCGA-25-1635 | 4.336986 | 1 | 6.621416 | 4.555412 | 5.226281 | 7.735338 | 0.151039 | low |
| TCGA-09-1661 | 3.20274 | 1 | 5.295873 | 3.915694 | 4.168465 | 9.568895 | 0.159813 | low |
| TCGA-13-1506 | 2.846575 | 1 | 5.522617 | 4.62728 | 6.216188 | 10.22234 | 0.115868 | low |
| TCGA-24-1565 | 0.854795 | 1 | 5.203655 | 4.402223 | 5.12109 | 9.339928 | 0.152259 | low |
| TCGA-24-1550 | 3.421918 | 1 | 5.643233 | 5.3296 | 4.616862 | 9.634437 | 0.16932 | high |
| TCGA-24-2026 | 2.90137 | 1 | 5.407603 | 4.407553 | 5.379325 | 6.230253 | 0.198309 | high |
| TCGA-13-0797 | 5.810959 | 0 | 4.835202 | 3.851935 | 4.445748 | 7.651924 | 0.197415 | high |
| TCGA-31-1959 | 0.183562 | 0 | 5.339882 | 4.989901 | 4.94706 | 10.25675 | 0.150139 | low |
| TCGA-30-1866 | 3.052055 | 1 | 5.449556 | 4.369068 | 5.607594 | 7.382003 | 0.167819 | high |
| TCGA-24-1842 | 0.693151 | 0 | 6.165928 | 4.502079 | 3.922949 | 8.022083 | 0.188247 | high |
| TCGA-61-1737 | 3.736986 | 0 | 5.704088 | 4.992281 | 5.062207 | 9.715589 | 0.149163 | low |
| TCGA-09-2054 | 1.745205 | 1 | 5.233224 | 5.513906 | 5.195825 | 10.26549 | 0.156344 | low |
| TCGA-25-1315 | 4.336986 | 1 | 5.676183 | 4.077342 | 4.734626 | 10.27185 | 0.132274 | low |
| TCGA-13-0762 | 9.315068 | 0 | 6.768752 | 3.345423 | 3.74527 | 9.595765 | 0.131124 | low |
| TCGA-09-1668 | 4.613699 | 0 | 5.310906 | 4.916115 | 5.113017 | 8.537686 | 0.17461 | high |
| TCGA-20-1686 | 0.243836 | 0 | 5.205993 | 2.835061 | 4.405967 | 6.393288 | 0.190476 | high |
| TCGA-VG-A8LO | 0.065753 | 1 | 6.212359 | 5.127742 | 4.107089 | 9.406874 | 0.169989 | high |
| TCGA-23-1030 | 2.427397 | 0 | 5.343591 | 4.333745 | 5.616313 | 9.554085 | 0.134397 | low |
| TCGA-24-2288 | 0.068493 | 1 | 5.31392 | 5.037565 | 4.537328 | 6.983315 | 0.228115 | high |
| TCGA-57-1586 | 1.860274 | 0 | 4.543471 | 4.275964 | 4.327226 | 9.282639 | 0.185184 | high |
| TCGA-13-1511 | 4.520548 | 1 | 4.484974 | 4.743757 | 5.411418 | 6.10186 | 0.234752 | high |
| TCGA-13-0766 | 4.726027 | 1 | 4.81358 | 4.549941 | 5.20292 | 10.53445 | 0.141887 | low |
| TCGA-13-0800 | 7.290411 | 0 | 4.935495 | 4.818922 | 6.272267 | 9.639401 | 0.134909 | low |
| TCGA-24-1431 | 1.59726 | 1 | 5.803021 | 4.478858 | 3.660055 | 7.164649 | 0.224095 | high |
| TCGA-23-1119 | 10.83014 | 0 | 5.696971 | 3.843835 | 4.972303 | 8.056284 | 0.156184 | low |
| TCGA-10-0928 | 1.542466 | 1 | 5.393593 | 5.524392 | 4.56854 | 5.094953 | 0.291744 | high |
| TCGA-24-1422 | 0.063014 | 1 | 5.584132 | 4.721174 | 5.446247 | 9.232497 | 0.145293 | low |
| TCGA-29-1695 | 3.367123 | 1 | 5.631047 | 4.742569 | 4.484555 | 7.741343 | 0.196402 | high |
| TCGA-30-1714 | 3.172603 | 1 | 5.242152 | 4.475797 | 5.711589 | 5.771379 | 0.203837 | high |
| TCGA-30-1860 | 3.742466 | 1 | 4.838875 | 4.8251 | 4.998377 | 10.92735 | 0.144897 | low |
| TCGA-24-2036 | 5.334247 | 1 | 5.18453 | 5.413739 | 5.104082 | 7.441933 | 0.212407 | high |
| TCGA-13-1485 | 1.723288 | 1 | 4.897783 | 4.476945 | 5.983327 | 8.134328 | 0.159154 | low |
| TCGA-23-1114 | 5.723288 | 1 | 5.294909 | 4.653708 | 5.067801 | 6.415241 | 0.213395 | high |
| TCGA-13-0887 | 5.556164 | 1 | 6.142066 | 4.201178 | 5.976896 | 10.2002 | 0.105504 | low |
| TCGA-13-1499 | 9.589041 | 0 | 5.330552 | 4.204206 | 5.611323 | 10.03731 | 0.125942 | low |
| TCGA-61-1995 | 0.167123 | 0 | 5.469409 | 5.445941 | 5.311226 | 6.991405 | 0.208973 | high |
| TCGA-57-1585 | 0.145205 | 1 | 5.486169 | 4.968042 | 4.337334 | 9.664966 | 0.171727 | high |
| TCGA-13-1510 | 3.723288 | 1 | 4.874406 | 4.744238 | 5.806769 | 9.034653 | 0.154181 | low |
| TCGA-04-1356 | 4.106849 | 1 | 5.693149 | 3.716673 | 5.856595 | 9.874029 | 0.110842 | low |
| TCGA-24-0966 | 0.635616 | 0 | 4.828295 | 4.203451 | 5.398155 | 10.72645 | 0.128959 | low |
| TCGA-24-1467 | 8.832877 | 1 | 5.021079 | 4.36424 | 4.257786 | 8.936168 | 0.184699 | high |
| TCGA-61-1911 | 3.542466 | 0 | 7.071399 | 4.570219 | 5.477745 | 9.766645 | 0.110894 | low |
| TCGA-24-1551 | 4.326027 | 1 | 5.428566 | 4.723425 | 5.310976 | 9.220579 | 0.151536 | low |
| TCGA-13-0883 | 5.745205 | 1 | 5.703242 | 5.410936 | 5.541907 | 8.93968 | 0.158626 | low |
| TCGA-23-1028 | 4.117808 | 0 | 5.465863 | 3.360277 | 6.248711 | 9.244606 | 0.109845 | low |
| TCGA-04-1361 | 2.709589 | 0 | 6.790294 | 4.084052 | 4.656431 | 9.732234 | 0.123041 | low |
| TCGA-29-1774 | 1.443836 | 0 | 5.589879 | 5.162337 | 5.371383 | 10.98081 | 0.129006 | low |
| TCGA-23-1809 | 0.043836 | 0 | 5.188471 | 5.412018 | 4.759526 | 9.673064 | 0.176677 | high |
| TCGA-29-1768 | 2.608219 | 1 | 5.363261 | 4.788666 | 4.977935 | 9.574154 | 0.15615 | low |
| TCGA-61-1733 | 2.649315 | 0 | 5.162333 | 5.230249 | 5.378227 | 9.848784 | 0.154736 | low |
| TCGA-29-1777 | 1.024658 | 0 | 4.490436 | 4.413253 | 5.806621 | 9.823262 | 0.142901 | low |
| TCGA-30-1892 | 4.065753 | 1 | 4.707092 | 4.514871 | 5.874825 | 8.155512 | 0.16622 | low |
| TCGA-23-1026 | 2.235616 | 0 | 5.135855 | 4.081368 | 5.562201 | 9.943831 | 0.129366 | low |
| TCGA-24-1419 | 0.654795 | 0 | 6.200622 | 4.342787 | 5.379341 | 5.701868 | 0.187984 | high |
| TCGA-13-0888 | 7.70137 | 1 | 4.749074 | 3.997785 | 4.66532 | 9.046339 | 0.169613 | high |
| TCGA-61-1918 | 1.312329 | 1 | 5.427613 | 4.976315 | 5.390606 | 9.112425 | 0.156333 | low |
| TCGA-13-1492 | 10.46301 | 1 | 5.562319 | 4.15847 | 5.634708 | 4.893567 | 0.208769 | high |
| TCGA-13-0885 | 9.282192 | 0 | 5.745526 | 3.876424 | 4.268803 | 8.621529 | 0.163463 | low |
| TCGA-04-1655 | 3.780822 | 1 | 4.722285 | 4.346413 | 5.800081 | 5.747473 | 0.211972 | high |
| TCGA-13-0720 | 3.712329 | 1 | 5.484511 | 4.08236 | 5.478977 | 9.667895 | 0.129046 | low |
| TCGA-24-0970 | 0.969863 | 1 | 4.151138 | 4.995923 | 5.347493 | 9.349126 | 0.181084 | high |
| TCGA-36-1568 | 2.39726 | 0 | 5.702313 | 5.262828 | 4.63896 | 8.805075 | 0.181318 | high |
| TCGA-04-1338 | 3.884932 | 0 | 4.183931 | 3.452133 | 5.180712 | 9.346325 | 0.152424 | low |
| TCGA-29-1785 | 3.024658 | 1 | 5.177527 | 5.215114 | 5.709176 | 7.79514 | 0.182115 | high |
| TCGA-24-1567 | 1.435616 | 1 | 6.0501 | 4.702042 | 4.875085 | 10.34052 | 0.132537 | low |
| TCGA-13-0765 | 3.805479 | 1 | 4.83646 | 4.223721 | 5.138686 | 9.210721 | 0.157752 | low |
| TCGA-09-1667 | 5.156164 | 0 | 7.600227 | 5.171853 | 5.394934 | 9.092506 | 0.121585 | low |
