# Supplementary material for: Glycosylation-Related Genes Predict the Prognosis and Immune Fraction of Ovarian Cancer Patients Based on Weighted Gene Coexpression Network Analysis (WGCNA) and Machine Learning
Source: Oxid Med Cell Longev. 2022 Mar 4;2022:3665617. doi: 10.1155/2022/3665617 (PMC8916863; doi:10.1155/2022/3665617)
Supplement: Supplementary Materials — Supplementary Table 1: the 636 glycosylation-related genes. Supplementary Table 2: the phenotypes of 70 OC patients for WGCNA. Supplementary Table 3: pathway signatures and genes. Supplementary Table 4: the expression of OGRmRNAs and the profiles of risk score in the training and test set. Supplementary Table 5: coexpressed genes with OGRmRNAs in ovarian cancer. Supplementary Table 6: results of GSEA by the “clusterProfiler” package. Supplementary Table 7: the expression of immune checkpoints of OC patients and their corresponding risk subtypes. Supplementary Figure 1: mRNAsi differences among low expression and high expression of OGRmRNAs and normal tissues. (A) ALG8. (B) DCTN4. (C) DCTN6. (D) UBB. Supplementary Figure 2: functional enrichment analysis with co-expressed genes of OGRmRNAs. (A) Gene Ontology terms. (B) Pathways of KEGG (Kyoto Encyclopedia of Genes and Genomes). [file 3665617.f1.zip › Supplementary Table 5.doc]

| ALG8 | DCTN4 | DCTN6 | UBB |
| --- | --- | --- | --- |
| CLNS1A | RBM22 | LEPROTL1 | None |
| INTS4 | FBXO38 | GTF2E2 |  |
| NDUFC2 | MFAP3 | PPP2CB |  |
| NARS2 | CSNK1A1 | SARAF |  |
| KCTD21 | GEMIN5 | PPP2R2A |  |
| RSF1 | G3BP1 | GSR |  |
| PAK1 | GRPEL2 | FBXO16 |  |
| AQP11 | FBXW11 |  |  |
| EMSY | LARS1 |  |  |
| THAP12 | CDC23 |  |  |
| USP35 | FAF2 |  |  |
| ACER3 | PWWP2A |  |  |
|  | SMAD5 |  |  |
|  | BRD8 |  |  |
|  | CNOT8 |  |  |
|  | HMGXB3 |  |  |
|  | FAM114A2 | |  |
|  | LARP1 |  |  |
|  | DELE1 |  |  |
|  | YIPF5 |  |  |
|  | KDM3B |  |  |
|  | LSM11 |  |  |
|  | MAML1 |  |  |
|  | HDAC3 |  |  |
|  | RBM12 |  |  |
|  | SLU7 |  |  |
|  | ZNF354C |  |  |
|  | ZNF346 |  |  |
|  | IK |  |  |
|  | AFF4 |  |  |
|  | ETF1 |  |  |
|  | FAM53C |  |  |
|  | PANK3 |  |  |
|  | HARS1 |  |  |
|  | NBAS |  |  |
|  | HARS2 |  |  |
|  | DHX9 |  |  |
|  | FAM13B |  |  |
|  | TAF7 |  |  |
|  | RARS1 |  |  |
|  | SAP30L |  |  |
|  | SMG8 |  |  |
|  | NBR1 |  |  |
|  | CNOT6 |  |  |
|  | NSD1 |  |  |
|  | CANX |  |  |
|  | RASA1 |  |  |
|  | GPATCH8 | |  |
|  | DNAJC18 |  |  |
|  | C5orf24 |  |  |
|  | KLHL11 |  |  |
|  | UBTD2 |  |  |
|  | CTTNBP2NL | |  |
|  | TSTD2 |  |  |
|  | NDST1 |  |  |
|  | TP53BP1 |  |  |
|  | KIAA1191 |  |  |
|  | FAN1 |  |  |
|  | RBM27 |  |  |
|  | KBTBD4 |  |  |
|  | MTREX |  |  |
|  | CCNT1 |  |  |
|  | UBLCP1 |  |  |
|  | ZRANB1 |  |  |
|  | RNF169 |  |  |
|  | CTNNA1 |  |  |
|  | AGGF1 |  |  |
|  | MAT2A |  |  |
|  | UIMC1 |  |  |
|  | ZFP62 |  |  |
|  | PRRC1 |  |  |
|  | ZNF207 |  |  |
|  | MTMR4 |  |  |
|  | UBR1 |  |  |
|  | KANSL1 |  |  |
|  | MSH3 |  |  |
|  | RNF145 |  |  |
|  | MED7 |  |  |
|  | ZFR |  |  |
|  | SF1 |  |  |
|  | VEZF1 |  |  |
|  | UBE4A |  |  |
|  | POMK |  |  |
|  | HEATR5B |  |  |
|  | RNF14 |  |  |
|  | CAPRIN1 |  |  |
|  | HNRNPK |  |  |
|  | MATR3 |  |  |
|  | TBC1D14 |  |  |
|  | HIPK1 |  |  |
|  | STAG1 |  |  |
|  | TRIP12 |  |  |
|  | HNRNPA0 | |  |
|  | IBTK |  |  |
|  | TNPO1 |  |  |
|  | PHAX |  |  |
|  | USP34 |  |  |
|  | USP37 |  |  |
|  | DHX57 |  |  |
|  | IPO11 |  |  |
|  | MED13 |  |  |
|  | SRSF1 |  |  |
|  | RTL6 |  |  |
|  | RBM14 |  |  |
|  | KAT7 |  |  |
|  | PRDM10 |  |  |
|  | HNRNPH1 | |  |
|  | DNAJC14 |  |  |
|  | LANCL1 |  |  |
|  | WDR36 |  |  |
|  | ASXL2 |  |  |
|  | SEC24C |  |  |
|  | MGA |  |  |
|  | FBXO11 |  |  |
|  | TCF12 |  |  |
|  | HMG20A |  |  |
|  | ZZZ3 |  |  |
|  | CCDC117 |  |  |
|  | PCDHGC3 | |  |
|  | TCERG1 |  |  |
|  | WDCP |  |  |
|  | WDR47 |  |  |
|  | PJA2 |  |  |
|  | SP2 |  |  |
|  | HSPA9 |  |  |
|  | APC |  |  |
|  | AP3B1 |  |  |
|  | GFM2 |  |  |
|  | KIAA0100 |  |  |
|  | HELZ |  |  |
|  | GNPDA1 |  |  |
|  | AP2B1 |  |  |
|  | KHDRBS1 |  |  |
|  | GIT2 |  |  |
|  | SEC24A |  |  |
|  | CEP120 |  |  |
|  | ANAPC1 |  |  |
|  | BECN1 |  |  |
|  | HSPA8 |  |  |
|  | AMMECR1L | |  |
|  | DHX8 |  |  |
|  | SLC36A1 |  |  |
|  | UBFD1 |  |  |
|  | XPC |  |  |
|  | MED1 |  |  |
|  | IKZF5 |  |  |
|  | ZNF619 |  |  |
|  | ZNF770 |  |  |
|  | ZBTB40 |  |  |
|  | REV1 |  |  |
|  | MIER3 |  |  |
|  | OXSR1 |  |  |
|  | ZNF106 |  |  |
|  | PRKAR1A |  |  |
|  | DNAJC27 |  |  |
|  | DDX6 |  |  |
|  | TGOLN2 |  |  |
|  | ADNP |  |  |
|  | POM121 |  |  |
|  | CNOT1 |  |  |
|  | RECQL |  |  |
|  | RFX7 |  |  |
|  | SECISBP2 |  |  |
|  | PLEKHM3 | |  |
|  | SF3B1 |  |  |
|  | KIF2A |  |  |
|  | ICE1 |  |  |
|  | GOSR2 |  |  |
|  | DDX1 |  |  |
|  | ISG20L2 |  |  |
|  | KLHL18 |  |  |
|  | MARF1 |  |  |
|  | SPG11 |  |  |
|  | SMG6 |  |  |
|  | ZNF143 |  |  |
|  | EIF3C |  |  |
|  | ZBTB5 |  |  |
|  | USP32 |  |  |
|  | KIAA0586 |  |  |
|  | WDR11 |  |  |
|  | ATF6 |  |  |
|  | LUZP1 |  |  |
|  | TEX2 |  |  |
|  | ASH1L |  |  |
|  | ZNF510 |  |  |
|  | CDC27 |  |  |
|  | ZNF629 |  |  |
|  | ZKSCAN5 |  |  |
|  | MTX3 |  |  |
|  | RETREG3 |  |  |
|  | MAPK14 |  |  |
|  | DOP1A |  |  |
|  | NR2C2 |  |  |
|  | FUBP1 |  |  |
|  | EXOC7 |  |  |
|  | OCRL |  |  |
|  | COPA |  |  |
|  | RAD17 |  |  |
|  | QRICH1 |  |  |
|  | VPS39 |  |  |
|  | CLTC |  |  |
|  | ZNF2 |  |  |
|  | NT5C2 |  |  |
|  | PTCD2 |  |  |
|  | RNF44 |  |  |
|  | ZSCAN29 |  |  |
|  | RSBN1 |  |  |
|  | ALMS1 |  |  |
