# Supplementary material for: Glycosylation-Related Genes Predict the Prognosis and Immune Fraction of Ovarian Cancer Patients Based on Weighted Gene Coexpression Network Analysis (WGCNA) and Machine Learning
Source: Oxid Med Cell Longev. 2022 Mar 4;2022:3665617. doi: 10.1155/2022/3665617 (PMC8916863; doi:10.1155/2022/3665617)
Supplement: Supplementary Materials — Supplementary Table 1: the 636 glycosylation-related genes. Supplementary Table 2: the phenotypes of 70 OC patients for WGCNA. Supplementary Table 3: pathway signatures and genes. Supplementary Table 4: the expression of OGRmRNAs and the profiles of risk score in the training and test set. Supplementary Table 5: coexpressed genes with OGRmRNAs in ovarian cancer. Supplementary Table 6: results of GSEA by the “clusterProfiler” package. Supplementary Table 7: the expression of immune checkpoints of OC patients and their corresponding risk subtypes. Supplementary Figure 1: mRNAsi differences among low expression and high expression of OGRmRNAs and normal tissues. (A) ALG8. (B) DCTN4. (C) DCTN6. (D) UBB. Supplementary Figure 2: functional enrichment analysis with co-expressed genes of OGRmRNAs. (A) Gene Ontology terms. (B) Pathways of KEGG (Kyoto Encyclopedia of Genes and Genomes). [file 3665617.f1.zip › Supplementary Table 6.doc]

| Description | setSize | enrichmentScore | NES | pvalue | p.adjust | qvalues |
| --- | --- | --- | --- | --- | --- | --- |
| KEGG_RIBOSOME | 85 | -0.60725 | -2.60444 | 1.00E-10 | 8.70E-09 | 7.63E-09 |
| KEGG_SYSTEMIC_LUPUS_ERYTHEMATOSUS | 113 | -0.70015 | -3.20241 | 1.00E-10 | 8.70E-09 | 7.63E-09 |
| KEGG_TYPE_I_DIABETES_MELLITUS | 37 | -0.75531 | -2.85972 | 1.21E-09 | 7.03E-08 | 6.16E-08 |
| KEGG_GRAFT_VERSUS_HOST_DISEASE | 30 | -0.79822 | -2.77566 | 2.20E-09 | 9.55E-08 | 8.38E-08 |
| KEGG_ALLOGRAFT_REJECTION | 31 | -0.7803 | -2.75909 | 3.23E-09 | 1.12E-07 | 9.86E-08 |
| KEGG_ANTIGEN_PROCESSING_AND_PRESENTATION | 57 | -0.61739 | -2.4365 | 2.44E-08 | 7.07E-07 | 6.20E-07 |
| KEGG_PROTEASOME | 42 | -0.6824 | -2.7035 | 5.56E-08 | 1.38E-06 | 1.21E-06 |
| KEGG_AUTOIMMUNE_THYROID_DISEASE | 30 | -0.73299 | -2.54883 | 4.80E-07 | 1.04E-05 | 9.16E-06 |
| KEGG_PPAR_SIGNALING_PATHWAY | 56 | -0.50529 | -1.97005 | 1.03E-05 | 0.000199 | 0.000175 |
| KEGG_NATURAL_KILLER_CELL_MEDIATED_CYTOTOXICITY | 96 | -0.43564 | -1.92659 | 1.89E-05 | 0.00033 | 0.000289 |
| KEGG_PRIMARY_IMMUNODEFICIENCY | 31 | -0.64499 | -2.28063 | 2.18E-05 | 0.000345 | 0.000303 |
| KEGG_LEISHMANIA_INFECTION | 67 | -0.47177 | -1.89942 | 4.89E-05 | 0.000709 | 0.000622 |
| KEGG_HEMATOPOIETIC_CELL_LINEAGE | 67 | -0.46827 | -1.88532 | 5.89E-05 | 0.000788 | 0.000691 |
| KEGG_ASTHMA | 19 | -0.71799 | -2.2324 | 8.41E-05 | 0.001045 | 0.000917 |
| KEGG_INTESTINAL_IMMUNE_NETWORK_FOR_IGA_PRODUCTION | 39 | -0.54997 | -2.07218 | 0.000198 | 0.002295 | 0.002013 |
| KEGG_NOD_LIKE_RECEPTOR_SIGNALING_PATHWAY | 57 | -0.46864 | -1.84949 | 0.00036 | 0.003919 | 0.003437 |
| KEGG_TOLL_LIKE_RECEPTOR_SIGNALING_PATHWAY | 85 | -0.35794 | -1.53517 | 0.003199 | 0.032747 | 0.028725 |
| KEGG_CYTOSOLIC_DNA_SENSING_PATHWAY | 40 | -0.4708 | -1.82173 | 0.005468 | 0.052857 | 0.046365 |
| KEGG_TRYPTOPHAN_METABOLISM | 34 | -0.47483 | -1.758 | 0.006251 | 0.057245 | 0.050215 |
| KEGG_METABOLISM_OF_XENOBIOTICS_BY_CYTOCHROME_P450 | 38 | -0.43817 | -1.65797 | 0.011716 | 0.101927 | 0.08941 |
| KEGG_RENIN_ANGIOTENSIN_SYSTEM | 13 | -0.59768 | -1.68388 | 0.015256 | 0.126407 | 0.110883 |
| KEGG_DRUG_METABOLISM_CYTOCHROME_P450 | 39 | -0.4073 | -1.53464 | 0.018068 | 0.141602 | 0.124212 |
| KEGG_FATTY_ACID_METABOLISM | 35 | -0.40847 | -1.52535 | 0.018718 | 0.141602 | 0.124212 |
| KEGG_TASTE_TRANSDUCTION | 29 | 0.58576 | 1.482013 | 0.020103 | 0.14512 | 0.127298 |
| KEGG_PORPHYRIN_AND_CHLOROPHYLL_METABOLISM | 23 | -0.46081 | -1.48047 | 0.020851 | 0.14512 | 0.127298 |
| KEGG_PRION_DISEASES | 30 | -0.44835 | -1.55904 | 0.026277 | 0.175856 | 0.15426 |
| KEGG_T_CELL_RECEPTOR_SIGNALING_PATHWAY | 99 | -0.30787 | -1.41314 | 0.032375 | 0.208638 | 0.183015 |
| KEGG_PROTEIN_EXPORT | 24 | -0.44252 | -1.43178 | 0.045734 | 0.284205 | 0.249303 |
| KEGG_RETINOL_METABOLISM | 28 | -0.45202 | -1.58341 | 0.048257 | 0.289544 | 0.253986 |
| KEGG_STEROID_BIOSYNTHESIS | 16 | -0.47432 | -1.3969 | 0.054535 | 0.30699 | 0.269289 |
| KEGG_FOLATE_BIOSYNTHESIS | 10 | -0.59333 | -1.50454 | 0.055829 | 0.30699 | 0.269289 |
| KEGG_DRUG_METABOLISM_OTHER_ENZYMES | 24 | -0.43374 | -1.40336 | 0.056458 | 0.30699 | 0.269289 |
| KEGG_NICOTINATE_AND_NICOTINAMIDE_METABOLISM | 22 | -0.42019 | -1.35429 | 0.074254 | 0.39152 | 0.343439 |
| KEGG_TYROSINE_METABOLISM | 32 | -0.35737 | -1.27616 | 0.077767 | 0.397984 | 0.349108 |
| KEGG_BIOSYNTHESIS_OF_UNSATURATED_FATTY_ACIDS | 21 | -0.43396 | -1.38879 | 0.094024 | 0.464852 | 0.407765 |
| KEGG_STEROID_HORMONE_BIOSYNTHESIS | 27 | -0.37774 | -1.28533 | 0.096176 | 0.464852 | 0.407765 |
| KEGG_PENTOSE_AND_GLUCURONATE_INTERCONVERSIONS | 11 | -0.52944 | -1.39911 | 0.101484 | 0.477249 | 0.418639 |
| KEGG_VALINE_LEUCINE_AND_ISOLEUCINE_DEGRADATION | 43 | -0.34309 | -1.37125 | 0.120388 | 0.551252 | 0.483554 |
| KEGG_SULFUR_METABOLISM | 11 | -0.52084 | -1.37638 | 0.13 | 0.56665 | 0.497061 |
| KEGG_VIRAL_MYOCARDITIS | 60 | -0.31061 | -1.23884 | 0.130264 | 0.56665 | 0.497061 |
| KEGG_GLUTATHIONE_METABOLISM | 45 | -0.33688 | -1.32235 | 0.137368 | 0.582977 | 0.511383 |
| KEGG_AXON_GUIDANCE | 122 | 0.399272 | 1.168036 | 0.15005 | 0.608381 | 0.533667 |
| KEGG_OLFACTORY_TRANSDUCTION | 27 | -0.35132 | -1.19543 | 0.150347 | 0.608381 | 0.533667 |
| KEGG_ECM_RECEPTOR_INTERACTION | 77 | 0.417466 | 1.177938 | 0.168539 | 0.666496 | 0.584646 |
| KEGG_PRIMARY_BILE_ACID_BIOSYNTHESIS | 10 | -0.49167 | -1.24675 | 0.185366 | 0.716748 | 0.628726 |
| KEGG_PENTOSE_PHOSPHATE_PATHWAY | 22 | -0.38349 | -1.23601 | 0.205882 | 0.778772 | 0.683134 |
| KEGG_LINOLEIC_ACID_METABOLISM | 17 | -0.40947 | -1.21872 | 0.225166 | 0.823452 | 0.722326 |
| KEGG_ARACHIDONIC_ACID_METABOLISM | 44 | -0.31461 | -1.24314 | 0.233138 | 0.823452 | 0.722326 |
| KEGG_GLYOXYLATE_AND_DICARBOXYLATE_METABOLISM | 14 | -0.42649 | -1.2369 | 0.235294 | 0.823452 | 0.722326 |
| KEGG_COMPLEMENT_AND_COAGULATION_CASCADES | 47 | -0.30961 | -1.16246 | 0.236624 | 0.823452 | 0.722326 |
| KEGG_GLYCOLYSIS_GLUCONEOGENESIS | 48 | -0.29941 | -1.14913 | 0.245989 | 0.839256 | 0.73619 |
| KEGG_BUTANOATE_METABOLISM | 28 | -0.34171 | -1.19702 | 0.251001 | 0.839889 | 0.736744 |
| KEGG_PROXIMAL_TUBULE_BICARBONATE_RECLAMATION | 20 | 0.484141 | 1.154037 | 0.279532 | 0.906614 | 0.795276 |
| KEGG_AMINO_SUGAR_AND_NUCLEOTIDE_SUGAR_METABOLISM | 42 | -0.27592 | -1.09311 | 0.281363 | 0.906614 | 0.795276 |
| KEGG_RNA_DEGRADATION | 56 | -0.26303 | -1.02552 | 0.288826 | 0.913741 | 0.801527 |
| KEGG_ALDOSTERONE_REGULATED_SODIUM_REABSORPTION | 36 | 0.431823 | 1.122418 | 0.294956 | 0.916471 | 0.803922 |
| KEGG_DILATED_CARDIOMYOPATHY | 76 | 0.388419 | 1.094608 | 0.310521 | 0.947906 | 0.831497 |
| KEGG_ETHER_LIPID_METABOLISM | 27 | -0.31659 | -1.07726 | 0.319328 | 0.955294 | 0.837977 |
| KEGG_PANTOTHENATE_AND_COA_BIOSYNTHESIS | 14 | -0.38241 | -1.10907 | 0.329412 | 0.955294 | 0.837977 |
| KEGG_TERPENOID_BACKBONE_BIOSYNTHESIS | 14 | -0.38343 | -1.11202 | 0.329412 | 0.955294 | 0.837977 |
| KEGG_RIG_I_LIKE_RECEPTOR_SIGNALING_PATHWAY | 52 | -0.27981 | -1.09305 | 0.334916 | 0.955334 | 0.838012 |
| KEGG_CIRCADIAN_RHYTHM_MAMMAL | 13 | 0.495474 | 1.090721 | 0.35109 | 0.985316 | 0.864312 |
| KEGG_NITROGEN_METABOLISM | 19 | 0.460736 | 1.086589 | 0.375878 | 1 | 0.877193 |
| KEGG_ALPHA_LINOLENIC_ACID_METABOLISM | 13 | -0.36976 | -1.04175 | 0.392045 | 1 | 0.877193 |
| KEGG_INOSITOL_PHOSPHATE_METABOLISM | 51 | 0.37948 | 1.032915 | 0.445026 | 1 | 0.877193 |
| KEGG_ENDOMETRIAL_CANCER | 50 | 0.368426 | 1.001103 | 0.49477 | 1 | 0.877193 |
| KEGG_MISMATCH_REPAIR | 22 | -0.31388 | -1.01163 | 0.5 | 1 | 0.877193 |
| KEGG_PEROXISOME | 70 | -0.23032 | -0.97416 | 0.532485 | 1 | 0.877193 |
| KEGG_NON_HOMOLOGOUS_END_JOINING | 12 | 0.454226 | 0.978471 | 0.539506 | 1 | 0.877193 |
| KEGG_HEDGEHOG_SIGNALING_PATHWAY | 46 | 0.358319 | 0.966356 | 0.562698 | 1 | 0.877193 |
| KEGG_THYROID_CANCER | 28 | -0.27197 | -0.9527 | 0.576271 | 1 | 0.877193 |
| KEGG_NOTCH_SIGNALING_PATHWAY | 45 | 0.35648 | 0.958601 | 0.586061 | 1 | 0.877193 |
| KEGG_ABC_TRANSPORTERS | 31 | 0.375107 | 0.956631 | 0.587248 | 1 | 0.877193 |
| KEGG_PHENYLALANINE_METABOLISM | 16 | -0.29126 | -0.85777 | 0.6 | 1 | 0.877193 |
| KEGG_ADHERENS_JUNCTION | 70 | 0.338251 | 0.9499 | 0.607362 | 1 | 0.877193 |
| KEGG_LIMONENE_AND_PINENE_DEGRADATION | 10 | -0.3445 | -0.87357 | 0.62439 | 1 | 0.877193 |
| KEGG_REGULATION_OF_AUTOPHAGY | 20 | -0.27969 | -0.88863 | 0.639456 | 1 | 0.877193 |
| KEGG_GLYCOSPHINGOLIPID_BIOSYNTHESIS_GLOBO_SERIES | 13 | 0.409043 | 0.900456 | 0.641646 | 1 | 0.877193 |
| KEGG_HYPERTROPHIC_CARDIOMYOPATHY_HCM | 70 | 0.324647 | 0.911696 | 0.665644 | 1 | 0.877193 |
| KEGG_CARDIAC_MUSCLE_CONTRACTION | 65 | 0.326625 | 0.90824 | 0.675926 | 1 | 0.877193 |
| KEGG_SPLICEOSOME | 126 | -0.16093 | -0.74517 | 0.68005 | 1 | 0.877193 |
| KEGG_MATURITY_ONSET_DIABETES_OF_THE_YOUNG | 13 | -0.29119 | -0.82038 | 0.6875 | 1 | 0.877193 |
| KEGG_STARCH_AND_SUCROSE_METABOLISM | 27 | -0.25565 | -0.86988 | 0.689076 | 1 | 0.877193 |
| KEGG_PHOSPHATIDYLINOSITOL_SIGNALING_SYSTEM | 70 | 0.319246 | 0.896529 | 0.692229 | 1 | 0.877193 |
| KEGG_DNA_REPLICATION | 35 | -0.24198 | -0.90362 | 0.705263 | 1 | 0.877193 |
| KEGG_CITRATE_CYCLE_TCA_CYCLE | 29 | -0.24364 | -0.86223 | 0.705357 | 1 | 0.877193 |
| KEGG_ERBB_SIGNALING_PATHWAY | 80 | 0.317431 | 0.901533 | 0.706002 | 1 | 0.877193 |
| KEGG_NON_SMALL_CELL_LUNG_CANCER | 53 | 0.322933 | 0.882436 | 0.709812 | 1 | 0.877193 |
| KEGG_MTOR_SIGNALING_PATHWAY | 49 | 0.322451 | 0.873335 | 0.712487 | 1 | 0.877193 |
| KEGG_AMYOTROPHIC_LATERAL_SCLEROSIS_ALS | 46 | 0.318664 | 0.859407 | 0.726027 | 1 | 0.877193 |
| KEGG_CHRONIC_MYELOID_LEUKEMIA | 71 | 0.311245 | 0.873284 | 0.732856 | 1 | 0.877193 |
| KEGG_LYSINE_DEGRADATION | 44 | 0.322224 | 0.861154 | 0.738854 | 1 | 0.877193 |
| KEGG_COLORECTAL_CANCER | 61 | 0.307843 | 0.855302 | 0.743827 | 1 | 0.877193 |
| KEGG_GAP_JUNCTION | 75 | 0.309262 | 0.871438 | 0.749744 | 1 | 0.877193 |
| KEGG_ONE_CARBON_POOL_BY_FOLATE | 17 | 0.345014 | 0.803016 | 0.753231 | 1 | 0.877193 |
| KEGG_BASAL_CELL_CARCINOMA | 49 | 0.311237 | 0.842964 | 0.75446 | 1 | 0.877193 |
| KEGG_ARRHYTHMOGENIC_RIGHT_VENTRICULAR_CARDIOMYOPATHY_ARVC | 63 | 0.304323 | 0.84646 | 0.764403 | 1 | 0.877193 |
| KEGG_GLYCOSYLPHOSPHATIDYLINOSITOL_GPI_ANCHOR_BIOSYNTHESIS | 24 | 0.327915 | 0.803718 | 0.764706 | 1 | 0.877193 |
| KEGG_VALINE_LEUCINE_AND_ISOLEUCINE_BIOSYNTHESIS | 10 | -0.30096 | -0.76316 | 0.780488 | 1 | 0.877193 |
| KEGG_OTHER_GLYCAN_DEGRADATION | 15 | 0.345905 | 0.786864 | 0.790036 | 1 | 0.877193 |
| KEGG_GLYCINE_SERINE_AND_THREONINE_METABOLISM | 26 | 0.321332 | 0.799504 | 0.793651 | 1 | 0.877193 |
| KEGG_SMALL_CELL_LUNG_CANCER | 82 | 0.297708 | 0.846028 | 0.793878 | 1 | 0.877193 |
| KEGG_NEUROACTIVE_LIGAND_RECEPTOR_INTERACTION | 151 | 0.300234 | 0.886486 | 0.794383 | 1 | 0.877193 |
| KEGG_NUCLEOTIDE_EXCISION_REPAIR | 43 | -0.21506 | -0.85955 | 0.796875 | 1 | 0.877193 |
| KEGG_GALACTOSE_METABOLISM | 21 | -0.23927 | -0.76574 | 0.798611 | 1 | 0.877193 |
| KEGG_LONG_TERM_DEPRESSION | 58 | 0.28947 | 0.801004 | 0.819588 | 1 | 0.877193 |
| KEGG_PYRUVATE_METABOLISM | 35 | -0.22291 | -0.83242 | 0.821053 | 1 | 0.877193 |
| KEGG_RENAL_CELL_CARCINOMA | 67 | 0.286835 | 0.80144 | 0.829243 | 1 | 0.877193 |
| KEGG_HISTIDINE_METABOLISM | 24 | -0.24039 | -0.77779 | 0.830508 | 1 | 0.877193 |
| KEGG_BASE_EXCISION_REPAIR | 34 | -0.21498 | -0.79595 | 0.848485 | 1 | 0.877193 |
| KEGG_ALANINE_ASPARTATE_AND_GLUTAMATE_METABOLISM | 28 | 0.295267 | 0.740464 | 0.859729 | 1 | 0.877193 |
| KEGG_JAK_STAT_SIGNALING_PATHWAY | 109 | -0.18353 | -0.85297 | 0.860142 | 1 | 0.877193 |
| KEGG_PYRIMIDINE_METABOLISM | 90 | -0.17514 | -0.73234 | 0.865357 | 1 | 0.877193 |
| KEGG_PROPANOATE_METABOLISM | 30 | 0.279034 | 0.711271 | 0.86845 | 1 | 0.877193 |
| KEGG_APOPTOSIS | 83 | -0.15674 | -0.66095 | 0.87255 | 1 | 0.877193 |
| KEGG_FOCAL_ADHESION | 185 | 0.28017 | 0.837798 | 0.876754 | 1 | 0.877193 |
| KEGG_TYPE_II_DIABETES_MELLITUS | 41 | 0.277345 | 0.731476 | 0.878919 | 1 | 0.877193 |
| KEGG_PANCREATIC_CANCER | 68 | 0.271331 | 0.759396 | 0.881148 | 1 | 0.877193 |
| KEGG_HOMOLOGOUS_RECOMBINATION | 26 | -0.22446 | -0.7506 | 0.883333 | 1 | 0.877193 |
| KEGG_FRUCTOSE_AND_MANNOSE_METABOLISM | 30 | -0.22723 | -0.79014 | 0.885714 | 1 | 0.877193 |
| KEGG_PROSTATE_CANCER | 84 | 0.266767 | 0.761375 | 0.886063 | 1 | 0.877193 |
| KEGG_GLYCOSAMINOGLYCAN_BIOSYNTHESIS_HEPARAN_SULFATE | 22 | 0.282917 | 0.685205 | 0.891455 | 1 | 0.877193 |
| KEGG_GLYCOSPHINGOLIPID_BIOSYNTHESIS_GANGLIO_SERIES | 15 | -0.23005 | -0.67396 | 0.899371 | 1 | 0.877193 |
| KEGG_GLYCOSPHINGOLIPID_BIOSYNTHESIS_LACTO_AND_NEOLACTO_SERIES | 22 | 0.275791 | 0.667945 | 0.908776 | 1 | 0.877193 |
| KEGG_PROGESTERONE_MEDIATED_OOCYTE_MATURATION | 81 | 0.258413 | 0.734266 | 0.911315 | 1 | 0.877193 |
| KEGG_LONG_TERM_POTENTIATION | 59 | 0.254792 | 0.706675 | 0.916495 | 1 | 0.877193 |
| KEGG_RIBOFLAVIN_METABOLISM | 14 | -0.22501 | -0.65259 | 0.929412 | 1 | 0.877193 |
| KEGG_SELENOAMINO_ACID_METABOLISM | 26 | 0.255624 | 0.636016 | 0.931973 | 1 | 0.877193 |
| KEGG_TIGHT_JUNCTION | 113 | 0.252904 | 0.736247 | 0.933333 | 1 | 0.877193 |
| KEGG_GLYCOSAMINOGLYCAN_BIOSYNTHESIS_KERATAN_SULFATE | 15 | 0.273069 | 0.621176 | 0.933571 | 1 | 0.877193 |
| KEGG_GLIOMA | 59 | 0.241489 | 0.669781 | 0.94433 | 1 | 0.877193 |
| KEGG_P53_SIGNALING_PATHWAY | 68 | 0.236901 | 0.663034 | 0.944672 | 1 | 0.877193 |
| KEGG_BLADDER_CANCER | 40 | -0.17789 | -0.68833 | 0.948718 | 1 | 0.877193 |
| KEGG_RNA_POLYMERASE | 26 | -0.20157 | -0.67406 | 0.95 | 1 | 0.877193 |
| KEGG_GLYCOSAMINOGLYCAN_BIOSYNTHESIS_CHONDROITIN_SULFATE | 21 | 0.25156 | 0.605783 | 0.951049 | 1 | 0.877193 |
| KEGG_ACUTE_MYELOID_LEUKEMIA | 56 | 0.231576 | 0.637206 | 0.952381 | 1 | 0.877193 |
| KEGG_BETA_ALANINE_METABOLISM | 18 | 0.245184 | 0.5762 | 0.955451 | 1 | 0.877193 |
| KEGG_CYSTEINE_AND_METHIONINE_METABOLISM | 29 | 0.233725 | 0.59134 | 0.960674 | 1 | 0.877193 |
| KEGG_GLYCOSAMINOGLYCAN_DEGRADATION | 19 | 0.247362 | 0.583372 | 0.961358 | 1 | 0.877193 |
| KEGG_CALCIUM_SIGNALING_PATHWAY | 134 | 0.235334 | 0.690827 | 0.973817 | 1 | 0.877193 |
| KEGG_GNRH_SIGNALING_PATHWAY | 86 | 0.228368 | 0.651416 | 0.974464 | 1 | 0.877193 |
| KEGG_O_GLYCAN_BIOSYNTHESIS | 26 | -0.18031 | -0.60295 | 0.975 | 1 | 0.877193 |
| KEGG_MELANOMA | 58 | 0.20382 | 0.563999 | 0.985567 | 1 | 0.877193 |
| KEGG_DORSO_VENTRAL_AXIS_FORMATION | 21 | 0.217971 | 0.524897 | 0.986014 | 1 | 0.877193 |
| KEGG_VIBRIO_CHOLERAE_INFECTION | 49 | 0.206065 | 0.558113 | 0.986359 | 1 | 0.877193 |
| KEGG_VEGF_SIGNALING_PATHWAY | 69 | 0.199903 | 0.560468 | 0.98773 | 1 | 0.877193 |
| KEGG_NEUROTROPHIN_SIGNALING_PATHWAY | 117 | 0.205521 | 0.599889 | 0.987915 | 1 | 0.877193 |
| KEGG_ARGININE_AND_PROLINE_METABOLISM | 49 | 0.198533 | 0.537713 | 0.988458 | 1 | 0.877193 |
| KEGG_AMINOACYL_TRNA_BIOSYNTHESIS | 41 | 0.195334 | 0.515179 | 0.991351 | 1 | 0.877193 |
| KEGG_ADIPOCYTOKINE_SIGNALING_PATHWAY | 61 | 0.198736 | 0.552164 | 0.99177 | 1 | 0.877193 |
| KEGG_PATHOGENIC_ESCHERICHIA_COLI_INFECTION | 50 | 0.187458 | 0.509369 | 0.993724 | 1 | 0.877193 |
| KEGG_INSULIN_SIGNALING_PATHWAY | 122 | 0.189831 | 0.555334 | 0.993958 | 1 | 0.877193 |
| KEGG_N_GLYCAN_BIOSYNTHESIS | 46 | 0.189471 | 0.510986 | 0.994731 | 1 | 0.877193 |
| KEGG_GLYCEROLIPID_METABOLISM | 41 | 0.174038 | 0.459011 | 0.996757 | 1 | 0.877193 |
| KEGG_GLYCEROPHOSPHOLIPID_METABOLISM | 68 | 0.185099 | 0.518052 | 0.996926 | 1 | 0.877193 |
| KEGG_MELANOGENESIS | 88 | 0.184455 | 0.527356 | 0.996933 | 1 | 0.877193 |
| KEGG_FC_GAMMA_R_MEDIATED_PHAGOCYTOSIS | 92 | 0.177457 | 0.508293 | 0.997955 | 1 | 0.877193 |
| KEGG_TGF_BETA_SIGNALING_PATHWAY | 80 | 0.183878 | 0.52223 | 0.997965 | 1 | 0.877193 |
| KEGG_VASOPRESSIN_REGULATED_WATER_REABSORPTION | 39 | 0.161284 | 0.424408 | 0.998919 | 1 | 0.877193 |
| KEGG_BASAL_TRANSCRIPTION_FACTORS | 31 | -0.1892 | -0.669 | 1 | 1 | 0.877193 |
| KEGG_B_CELL_RECEPTOR_SIGNALING_PATHWAY | 74 | -0.16627 | -0.71476 | 1 | 1 | 0.877193 |
| KEGG_ENDOCYTOSIS | 175 | 0.14785 | 0.440346 | 1 | 1 | 0.877193 |
| KEGG_EPITHELIAL_CELL_SIGNALING_IN_HELICOBACTER_PYLORI_INFECTION | 62 | -0.17856 | -0.69345 | 1 | 1 | 0.877193 |
| KEGG_FC_EPSILON_RI_SIGNALING_PATHWAY | 66 | 0.137358 | 0.382349 | 1 | 1 | 0.877193 |
| KEGG_LEUKOCYTE_TRANSENDOTHELIAL_MIGRATION | 104 | -0.14559 | -0.6809 | 1 | 1 | 0.877193 |
| KEGG_MAPK_SIGNALING_PATHWAY | 235 | 0.151298 | 0.455519 | 1 | 1 | 0.877193 |
| KEGG_OOCYTE_MEIOSIS | 102 | 0.16253 | 0.470305 | 1 | 1 | 0.877193 |
| KEGG_PATHWAYS_IN_CANCER | 298 | 0.179249 | 0.543237 | 1 | 1 | 0.877193 |
| KEGG_PURINE_METABOLISM | 140 | 0.097923 | 0.288024 | 1 | 1 | 0.877193 |
| KEGG_REGULATION_OF_ACTIN_CYTOSKELETON | 184 | 0.156085 | 0.466588 | 1 | 1 | 0.877193 |
| KEGG_SNARE_INTERACTIONS_IN_VESICULAR_TRANSPORT | 38 | 0.130528 | 0.342415 | 1 | 1 | 0.877193 |
| KEGG_SPHINGOLIPID_METABOLISM | 35 | 0.148807 | 0.385284 | 1 | 1 | 0.877193 |
| KEGG_VASCULAR_SMOOTH_MUSCLE_CONTRACTION | 100 | -0.20616 | -0.95667 | 1 | 1 | 0.877193 |
| KEGG_WNT_SIGNALING_PATHWAY | 139 | 0.179506 | 0.527658 | 1 | 1 | 0.877193 |
