# Supplementary material for: Glycosylation-Related Genes Predict the Prognosis and Immune Fraction of Ovarian Cancer Patients Based on Weighted Gene Coexpression Network Analysis (WGCNA) and Machine Learning
Source: Oxid Med Cell Longev. 2022 Mar 4;2022:3665617. doi: 10.1155/2022/3665617 (PMC8916863; doi:10.1155/2022/3665617)
Supplement: Supplementary Materials — Supplementary Table 1: the 636 glycosylation-related genes. Supplementary Table 2: the phenotypes of 70 OC patients for WGCNA. Supplementary Table 3: pathway signatures and genes. Supplementary Table 4: the expression of OGRmRNAs and the profiles of risk score in the training and test set. Supplementary Table 5: coexpressed genes with OGRmRNAs in ovarian cancer. Supplementary Table 6: results of GSEA by the “clusterProfiler” package. Supplementary Table 7: the expression of immune checkpoints of OC patients and their corresponding risk subtypes. Supplementary Figure 1: mRNAsi differences among low expression and high expression of OGRmRNAs and normal tissues. (A) ALG8. (B) DCTN4. (C) DCTN6. (D) UBB. Supplementary Figure 2: functional enrichment analysis with co-expressed genes of OGRmRNAs. (A) Gene Ontology terms. (B) Pathways of KEGG (Kyoto Encyclopedia of Genes and Genomes). [file 3665617.f1.zip › Supplementary Table 7.doc]

| id | CD244 | LAG3 | ICOS | CTLA4 | CD48 | CD160 | ICOSLG | TMIGD2 | IDO1 | HHLA2 | BTNL2 | CD70 | CD27 | TNFRSF25 | TIGIT | risk |
| --- | --- | --- | --- | --- | --- | --- | --- | --- | --- | --- | --- | --- | --- | --- | --- | --- |
| TCGA-25-1313 | 0.489442 | 4.243594 | 0.554433 | 0.853111 | 3.639395 | 0.331482 | 0.367196 | 0.266871 | 5.19258 | 0.486086 | 0.081649 | 0.962372 | 2.021988 | 3.090147 | 0.258782 | low |
| TCGA-61-2000 | 0.293048 | 3.405382 | 0.588741 | 1.416506 | 3.07251 | 0.12776 | 1.149572 | 0.662408 | 2.690323 | 0 | 0.129518 | 0.879738 | 1.714493 | 3.384532 | 0.640064 | high |
| TCGA-23-1027 | 0.383232 | 2.615068 | 0.585386 | 0.878691 | 3.604473 | 0.076869 | 1.200631 | 0.426965 | 3.531026 | 0.015632 | 0.038318 | 0.888394 | 1.652489 | 4.101904 | 0.351287 | low |
| TCGA-61-2012 | 0.966045 | 3.442455 | 1.577012 | 1.883179 | 3.807811 | 0.105975 | 0.169161 | 0.163395 | 5.301085 | 0.949997 | 0.048744 | 1.491111 | 4.79687 | 5.034597 | 1.558897 | low |
| TCGA-24-1546 | 0.269719 | 1.078567 | 0.307907 | 0.654313 | 3.335945 | 0.225314 | 0.7104 | 0.221829 | 4.418896 | 0.016591 | 0 | 0.928774 | 2.457513 | 3.605 | 0.484884 | high |
| TCGA-13-1487 | 0.034303 | 1.38938 | 0.209072 | 0.369432 | 1.530954 | 0 | 0.956731 | 0.076523 | 2.445758 | 0.053678 | 0.303514 | 0.238166 | 1.536786 | 2.254718 | 0.280294 | high |
| TCGA-29-1710 | 2.379159 | 3.216062 | 2.132868 | 2.061047 | 4.963878 | 0.960096 | 0.236854 | 3.472788 | 3.489241 | 0.332023 | 0.074889 | 1.397114 | 4.228897 | 3.733058 | 2.112986 | high |
| TCGA-24-1544 | 0.125424 | 1.98986 | 0.218489 | 0.113171 | 2.518326 | 0.309885 | 0.534021 | 0 | 2.203964 | 0.389602 | 0.91368 | 0 | 1.474594 | 4.381802 | 0.173524 | high |
| TCGA-24-2267 | 0.941622 | 4.93138 | 2.079476 | 2.213985 | 4.498449 | 0.807951 | 0.33282 | 1.390189 | 7.593621 | 0.521188 | 0.056332 | 1.595605 | 4.575458 | 4.287593 | 2.095069 | high |
| TCGA-13-0795 | 0.151738 | 2.612382 | 0.730209 | 0.885149 | 4.454231 | 0.262415 | 0.768305 | 0.626676 | 2.004384 | 0.158961 | 0 | 1.02442 | 2.496795 | 4.034996 | 0.47525 | high |
| TCGA-25-2396 | 0.604171 | 4.653318 | 1.397422 | 1.662873 | 3.473619 | 0.281487 | 0.28278 | 1.257934 | 3.259254 | 0.184677 | 0.043587 | 1.061778 | 2.903583 | 2.826018 | 1.096575 | high |
| TCGA-09-0366 | 1.287284 | 4.887691 | 0.364957 | 0.752391 | 4.791375 | 0.897449 | 0.450706 | 0.958013 | 3.39122 | 0.135398 | 0.216698 | 1.155021 | 2.095424 | 5.320527 | 0.596171 | low |
| TCGA-61-2002 | 1.411341 | 3.437638 | 2.362134 | 2.003153 | 4.537688 | 0.21386 | 0.856955 | 0.965431 | 7.035654 | 0.546338 | 0 | 2.101515 | 3.927032 | 3.565847 | 2.162235 | low |
| TCGA-23-1113 | 0.034707 | 0.607441 | 0 | 0.04658 | 1.477571 | 0.296 | 1.074856 | 0.07741 | 0.768895 | 0.106641 | 0 | 0.064066 | 0.530099 | 4.316648 | 0.048603 | low |
| TCGA-24-1428 | 0.82706 | 3.683608 | 1.311436 | 1.589763 | 3.595467 | 0.630274 | 0.609211 | 2.180549 | 6.256192 | 0.076599 | 0.017562 | 1.015099 | 3.460741 | 5.216445 | 1.118948 | low |
| TCGA-30-1853 | 0.112564 | 1.265128 | 0.031273 | 0 | 0.546247 | 0.020905 | 0.645788 | 0 | 0.299899 | 0 | 0.396005 | 0.295133 | 1.132306 | 2.888596 | 0.013663 | high |
| TCGA-25-1626 | 1.554547 | 3.632967 | 2.052582 | 2.407223 | 4.471607 | 0.538118 | 0.317248 | 1.494488 | 3.797666 | 0.039135 | 0.226318 | 1.429 | 4.028008 | 4.326673 | 2.039501 | high |
| TCGA-13-0714 | 0.186863 | 1.332883 | 0.291668 | 0.407222 | 2.657552 | 0.348916 | 0.13413 | 0.95545 | 2.831529 | 0.251719 | 0 | 0.976054 | 2.029351 | 2.817681 | 0.707011 | high |
| TCGA-25-1321 | 0.062878 | 1.648178 | 0.179286 | 0.163585 | 2.052673 | 0.253749 | 0.489644 | 0 | 1.010591 | 0.593406 | 0.176381 | 0.41392 | 2.267124 | 4.270265 | 0.884595 | low |
| TCGA-04-1530 | 0.474101 | 3.199069 | 1.209912 | 1.469638 | 4.538367 | 0.558119 | 0.443364 | 0.287107 | 3.463867 | 0.275829 | 0.191524 | 1.030661 | 2.907581 | 5.496663 | 1.420397 | high |
| TCGA-57-1993 | 0.123953 | 5.212842 | 0.164959 | 0.203054 | 1.887914 | 0.866305 | 0.088428 | 0.139539 | 1.329459 | 1.721057 | 0 | 4.502508 | 2.032238 | 5.567812 | 0.429938 | high |
| TCGA-13-0886 | 0.968696 | 3.197964 | 1.11171 | 1.757513 | 3.928239 | 0.386494 | 0.121917 | 1.20164 | 5.562137 | 0.393036 | 0.118981 | 1.229885 | 2.884525 | 3.015483 | 1.209277 | low |
| TCGA-09-0369 | 0.051618 | 1.734253 | 0.073768 | 0.591933 | 1.601401 | 0.210697 | 0.64187 | 0.350366 | 1.980652 | 0.067394 | 0.033696 | 0.561224 | 1.234606 | 4.973329 | 0.087665 | low |
| TCGA-24-1434 | 0.84673 | 2.388958 | 1.510972 | 1.923754 | 4.052912 | 0.611749 | 0.39787 | 0.740306 | 2.650796 | 0.16572 | 0.137831 | 0.656608 | 2.5936 | 3.163996 | 1.181721 | low |
| TCGA-23-1022 | 0.041893 | 0.580929 | 0 | 0 | 1.001967 | 0 | 0.044238 | 0.222501 | 1.749292 | 0.202708 | 0.040782 | 0.185696 | 0.376849 | 2.03723 | 0 | low |
| TCGA-25-1317 | 0.258189 | 1.941045 | 0.446667 | 0.681379 | 1.56885 | 0.252604 | 1.433662 | 0.212198 | 3.069575 | 0.107267 | 0.883265 | 0.334628 | 2.66276 | 3.879606 | 0.366143 | high |
| TCGA-13-0727 | 0.212744 | 2.121179 | 0.106051 | 0.192512 | 2.846629 | 0.686781 | 1.255148 | 0.163373 | 0.85144 | 0.152186 | 0.141533 | 0.546887 | 0.894166 | 4.132794 | 0.069957 | high |
| TCGA-59-2351 | 0.508314 | 2.565801 | 1.167288 | 0.784487 | 3.628721 | 0.204074 | 0.277095 | 0.88432 | 2.699094 | 0.679377 | 0 | 0.710368 | 4.08729 | 2.353512 | 1.361619 | low |
| TCGA-04-1542 | 0 | 0.490575 | 0.033366 | 0 | 0.482269 | 0.06592 | 0.350162 | 0 | 0.423944 | 0.184595 | 0.419265 | 0 | 1.006813 | 2.083373 | 0.043317 | low |
| TCGA-24-2035 | 1.153249 | 2.768594 | 1.469948 | 1.774562 | 3.843263 | 0.582253 | 0.19669 | 1.752325 | 4.171638 | 0.06579 | 0.206273 | 1.506416 | 3.251173 | 2.975922 | 1.103245 | low |
| TCGA-13-0908 | 0.309633 | 3.715071 | 1.874779 | 1.71908 | 3.14034 | 0.377562 | 0.219374 | 0.262634 | 2.471948 | 0.136516 | 0.102407 | 0.938379 | 2.7591 | 3.302471 | 1.288069 | low |
| TCGA-24-2027 | 0.1306 | 2.030512 | 0.118075 | 0.145881 | 1.377677 | 0.949823 | 0.208848 | 0.192824 | 1.581231 | 0.291488 | 0.127236 | 2.891405 | 1.413518 | 4.769029 | 0.180563 | low |
| TCGA-04-1648 | 0.66839 | 1.100459 | 0.19773 | 0.291597 | 2.700091 | 0.771154 | 0.159767 | 1.560632 | 5.768772 | 0.349087 | 0.719806 | 1.030417 | 1.472895 | 3.967481 | 0.383787 | high |
| TCGA-20-1683 | 0.226922 | 3.23877 | 0.710949 | 0.857787 | 2.487445 | 1.43631 | 0.090341 | 0.27219 | 1.59473 | 0.029477 | 1.324532 | 1.053209 | 5.353215 | 3.399782 | 0.540814 | high |
| TCGA-24-1928 | 0.502568 | 2.826864 | 0.926205 | 1.434528 | 2.62604 | 0.521283 | 0.040422 | 0.349251 | 2.986451 | 0.131329 | 0 | 3.579594 | 2.903584 | 4.307533 | 0.868701 | high |
| TCGA-24-1557 | 0.135281 | 2.39879 | 0.614411 | 0.339098 | 2.803965 | 0.339402 | 1.211639 | 0.289898 | 3.524516 | 0.054805 | 0.78028 | 0.450657 | 1.229316 | 3.691276 | 0.440805 | high |
| TCGA-29-1770 | 0.189678 | 1.133682 | 0.238012 | 0.048834 | 2.363607 | 0.382142 | 0.31382 | 0.576862 | 1.391057 | 0.028744 | 0.103772 | 0.538641 | 0.969121 | 2.553723 | 0.245783 | high |
| TCGA-13-0913 | 0.850026 | 2.569125 | 2.000238 | 1.246534 | 4.467099 | 0.262875 | 0.346921 | 0.822655 | 5.31394 | 0 | 0 | 1.066049 | 2.585795 | 2.021862 | 0.632424 | low |
| TCGA-04-1651 | 0.105028 | 2.025389 | 0.279907 | 0.556479 | 1.569822 | 0.331214 | 0.673769 | 0.751745 | 5.991043 | 0.073201 | 0 | 0.255365 | 1.392734 | 3.573256 | 0.374788 | low |
| TCGA-24-2293 | 1.297527 | 2.748602 | 1.050658 | 1.163613 | 4.20784 | 0.49849 | 0.498358 | 1.346085 | 4.73341 | 0.190567 | 0.217625 | 1.238522 | 3.187848 | 4.294535 | 1.148262 | high |
| TCGA-61-1728 | 0.226348 | 1.914267 | 0.324571 | 0.599393 | 2.707301 | 0.185857 | 0.390041 | 0.392271 | 4.341477 | 0.126963 | 0.246057 | 3.544953 | 1.857785 | 3.569187 | 0.343593 | high |
| TCGA-04-1331 | 0.256866 | 2.765971 | 0.490373 | 0.510199 | 2.171794 | 0.581233 | 0.983299 | 0.267384 | 3.427375 | 0.033675 | 0.121048 | 0.640734 | 1.527695 | 4.411998 | 0.29279 | high |
| TCGA-25-2393 | 0.476807 | 2.81301 | 0.492398 | 1.201159 | 2.599534 | 0.176393 | 0.308946 | 0.423337 | 5.232546 | 0.0669 | 0.159976 | 1.196763 | 2.211709 | 3.259864 | 0.586566 | low |
| TCGA-29-1697 | 0.698547 | 2.739313 | 0.332011 | 0.133372 | 1.823745 | 0.463821 | 1.879518 | 0.346159 | 5.199832 | 0.294486 | 0.70436 | 0.680889 | 1.338604 | 4.407977 | 0.21777 | low |
| TCGA-25-2399 | 0.911456 | 4.076965 | 0.93833 | 1.369007 | 3.445457 | 0.555313 | 0.402541 | 0.943587 | 4.593344 | 0.124266 | 0.498501 | 0.572183 | 2.704935 | 4.840273 | 0.794597 | high |
| TCGA-09-2045 | 0.423874 | 2.710166 | 0.342885 | 0.370598 | 2.770842 | 0.554927 | 0.898453 | 0.507646 | 4.562639 | 0.166435 | 0 | 0.231065 | 1.937707 | 4.542282 | 0.493407 | high |
| TCGA-23-2078 | 1.065641 | 3.531542 | 1.806826 | 1.961558 | 4.560318 | 1.010827 | 0.895077 | 1.143093 | 4.199422 | 0.176523 | 0.260502 | 1.411017 | 4.003655 | 4.435683 | 1.528047 | low |
| TCGA-61-1725 | 0.719658 | 1.804996 | 0.544464 | 0.725726 | 3.834274 | 0.061605 | 0.32102 | 0.588168 | 4.484879 | 0.045223 | 0.210822 | 2.34224 | 2.13726 | 2.200458 | 0.717808 | low |
| TCGA-29-1691 | 0.48523 | 2.334789 | 0.786511 | 1.182709 | 2.893261 | 0.353183 | 0.781835 | 0.331824 | 2.962156 | 0.124302 | 0.078034 | 0.45712 | 2.294513 | 4.5806 | 1.246711 | high |
| TCGA-29-2414 | 0.569108 | 2.950485 | 1.13774 | 1.315971 | 2.919445 | 0.270213 | 0.116142 | 0.35623 | 2.724101 | 0.041174 | 1.146939 | 0.902061 | 2.95648 | 3.576176 | 1.171758 | high |
| TCGA-29-1688 | 0.836213 | 3.391597 | 0.945176 | 1.245547 | 4.667158 | 0.726771 | 0.233963 | 1.295537 | 6.322245 | 0.059603 | 0.049265 | 0.764697 | 2.12548 | 3.149646 | 0.830749 | low |
| TCGA-25-1870 | 0.324357 | 1.431336 | 0.262669 | 0.294546 | 2.637202 | 0.557378 | 0.559409 | 0.340668 | 0.91341 | 0.994887 | 0.177393 | 0.248607 | 2.412784 | 3.876724 | 0.181443 | high |
| TCGA-57-1994 | 1.394225 | 3.892956 | 2.206897 | 2.672545 | 4.298756 | 1.268189 | 0.278238 | 0.621523 | 3.240029 | 1.235616 | 0.745533 | 1.241781 | 4.515654 | 5.921583 | 2.27648 | low |
| TCGA-24-1470 | 0.988018 | 3.627662 | 2.681062 | 3.04335 | 4.705292 | 0.897355 | 0.555667 | 1.516233 | 5.823432 | 0.827553 | 0.17772 | 1.437449 | 4.232509 | 4.909242 | 2.166117 | high |
| TCGA-25-2042 | 0.627907 | 1.940337 | 0.943219 | 1.231011 | 2.409939 | 0.463897 | 0.190145 | 0.693559 | 1.50962 | 0.040734 | 0.098626 | 1.551997 | 2.910584 | 3.348469 | 1.010503 | high |
| TCGA-13-0923 | 0.152726 | 4.264323 | 0.625534 | 0.909081 | 2.277711 | 0.111487 | 0.082713 | 0.630159 | 5.108859 | 0.020997 | 0.283716 | 1.234875 | 2.798743 | 2.183366 | 0.891815 | low |
| TCGA-13-1477 | 0.075394 | 1.815702 | 0.139352 | 0.346104 | 1.021443 | 0.207136 | 0.590302 | 0.241451 | 1.18955 | 0.601866 | 0.037175 | 0.263152 | 1.09856 | 2.246654 | 0.194962 | high |
| TCGA-24-2254 | 0.434494 | 2.752111 | 0.12115 | 0.486197 | 3.123346 | 0.715315 | 0.965206 | 0.385158 | 2.55557 | 0.117931 | 0.559258 | 1.518704 | 2.32155 | 4.645585 | 0.172234 | low |
| TCGA-25-1631 | 0.140456 | 0.819474 | 0.151144 | 0.235327 | 1.633793 | 0.249961 | 0.987603 | 0.136466 | 4.857441 | 0.157128 | 0.261838 | 0.549036 | 1.344335 | 3.164726 | 0.333844 | low |
| TCGA-30-1861 | 0.116377 | 0.946174 | 0.240602 | 0.240183 | 1.684341 | 0.312394 | 0.570808 | 0.155893 | 2.389413 | 0.179318 | 0 | 0.42725 | 1.039406 | 3.33848 | 0.201305 | high |
| TCGA-13-0730 | 0.185556 | 3.141985 | 0.374637 | 1.58385 | 2.620649 | 0.186819 | 0.219585 | 0.086982 | 3.527821 | 0.030863 | 0.146459 | 0.206117 | 1.98112 | 5.484713 | 0.270249 | high |
| TCGA-13-0905 | 0.181321 | 2.236133 | 0.434356 | 0.537244 | 2.037475 | 0.077323 | 1.681344 | 0.06117 | 6.009374 | 0.221285 | 0.551318 | 1.012093 | 1.929979 | 3.001854 | 0.503126 | high |
| TCGA-24-1924 | 0.988438 | 4.069754 | 2.447853 | 2.694774 | 4.921005 | 0.195633 | 0.252302 | 0.703396 | 6.300349 | 0.249522 | 0 | 1.515328 | 3.495212 | 3.643778 | 1.469537 | low |
| TCGA-31-1953 | 1.743602 | 4.456529 | 2.015906 | 2.430747 | 4.716244 | 0.508381 | 1.447423 | 2.393339 | 4.713303 | 0.142828 | 0.080163 | 1.244136 | 2.954547 | 4.266835 | 1.919036 | high |
| TCGA-04-1343 | 0.294146 | 2.752211 | 0.573928 | 1.457103 | 3.00432 | 0.297811 | 0.615746 | 0.504777 | 3.843628 | 0.05726 | 0.111067 | 0.997752 | 3.173034 | 4.386617 | 0.591002 | low |
| TCGA-25-1316 | 0.787948 | 2.912002 | 0.797999 | 0.299133 | 2.279218 | 0.682376 | 1.160078 | 0.438076 | 3.141825 | 0.13882 | 0.039459 | 1.006944 | 2.900447 | 2.414754 | 2.611721 | low |
| TCGA-25-2401 | 0.898063 | 3.813375 | 1.795708 | 1.69611 | 4.704602 | 0.407281 | 1.510007 | 1.086864 | 5.954172 | 0.024328 | 0.324347 | 1.08903 | 3.272237 | 3.458987 | 1.502268 | low |
| TCGA-24-2297 | 0.265002 | 2.31603 | 0.340504 | 0.429983 | 1.509714 | 0.820204 | 0.12534 | 0.139391 | 4.305586 | 0.878351 | 0.310125 | 0.222959 | 2.109002 | 4.950915 | 0.383777 | high |
| TCGA-29-1693 | 0.294997 | 2.773581 | 0.861472 | 0.84863 | 3.244225 | 0.265556 | 0.10584 | 0.62114 | 5.595894 | 0.095023 | 0.03002 | 0.549329 | 1.494151 | 3.229959 | 0.260191 | low |
| TCGA-29-1778 | 0.593237 | 3.788093 | 1.126519 | 1.552533 | 3.125563 | 0.270742 | 0.69211 | 0.915134 | 5.253893 | 0.897577 | 0 | 0.830485 | 3.802049 | 6.030001 | 1.38341 | low |
| TCGA-61-2101 | 0.134339 | 0.919968 | 0.261965 | 0.4646 | 1.522819 | 0.179668 | 1.293698 | 0.198226 | 2.677162 | 0.184802 | 0 | 0.165209 | 1.979707 | 3.13818 | 0.33854 | high |
| TCGA-13-0911 | 0.368441 | 2.192237 | 0.754872 | 0.7951 | 4.750259 | 0.109026 | 0.822721 | 0.89881 | 2.88532 | 0.04075 | 0 | 1.297625 | 2.305272 | 2.875473 | 0.765051 | low |
| TCGA-13-1483 | 0.211051 | 2.195305 | 0.814889 | 0.886886 | 2.690723 | 0.154892 | 0.910261 | 0.253422 | 4.850355 | 0.067262 | 0.033629 | 0.515375 | 1.757745 | 3.424594 | 0.574162 | low |
| TCGA-24-1471 | 0.235483 | 1.024317 | 0.505384 | 0.660177 | 2.647957 | 0.409272 | 0.115406 | 0.446952 | 2.39117 | 0.092891 | 0.11391 | 0.230443 | 1.602063 | 3.647453 | 0.37078 | high |
| TCGA-13-0920 | 0.036396 | 2.745994 | 0.471292 | 0.42686 | 2.184231 | 0.12685 | 0.124332 | 0.230838 | 3.094783 | 0.240159 | 0.070008 | 0.362747 | 2.51371 | 2.640401 | 0.730403 | low |
| TCGA-24-0968 | 0.825324 | 3.42712 | 0.8874 | 1.014557 | 3.96221 | 0.227601 | 0.183923 | 1.005906 | 3.57747 | 0.356865 | 0.19466 | 1.219605 | 2.509029 | 2.485572 | 0.9352 | low |
| TCGA-29-1781 | 1.507709 | 4.407542 | 2.144754 | 1.681869 | 5.211802 | 0.339456 | 0.929361 | 1.014512 | 5.251771 | 0.060778 | 0.074718 | 2.198767 | 5.05709 | 3.766267 | 1.498535 | low |
| TCGA-23-1029 | 0.221825 | 3.395181 | 0.606223 | 0.53404 | 1.983136 | 0.296653 | 0.233466 | 0.140936 | 5.036093 | 1.635367 | 0.234427 | 0.32608 | 1.811687 | 3.851101 | 0.344053 | low |
| TCGA-24-1843 | 0.356555 | 4.144152 | 0.777222 | 1.111353 | 4.352185 | 0.230076 | 0.84007 | 0.422257 | 2.022052 | 1.370357 | 0 | 0.937331 | 4.328531 | 3.774278 | 0.789085 | low |
| TCGA-04-1332 | 0.173434 | 3.863376 | 0.15702 | 0.395724 | 2.276705 | 0.451664 | 1.107691 | 0.658435 | 2.908748 | 0.635442 | 0 | 0.110238 | 1.376359 | 3.896706 | 0.124162 | high |
| TCGA-13-1495 | 0.31597 | 2.356222 | 0.062231 | 0.077236 | 2.080207 | 0.082321 | 0.42248 | 0.317718 | 5.052118 | 0.132819 | 0.277136 | 0.730794 | 1.799339 | 3.252535 | 0.335249 | high |
| TCGA-OY-A56Q | 0.041865 | 0.846613 | 0 | 0 | 0.45687 | 0.145247 | 0.208628 | 0.222364 | 0.403217 | 0.71177 | 0 | 0.150325 | 1.540594 | 4.715979 | 0.105624 | high |
| TCGA-29-1703 | 0.28463 | 2.451547 | 0.592062 | 0.963164 | 2.169588 | 0.051318 | 0.340227 | 0.503638 | 3.43934 | 0.307862 | 1.09948 | 0.644466 | 1.908234 | 3.70328 | 0.654848 | high |
| TCGA-13-A5FT | 0.020372 | 2.464725 | 0.63504 | 0.867164 | 2.16976 | 0.536232 | 2.361213 | 0.293203 | 5.382959 | 0.138573 | 0.288548 | 0.80121 | 1.59313 | 4.024556 | 0.771506 | high |
| TCGA-24-0982 | 0.13614 | 2.920042 | 0.469691 | 0.512746 | 1.918644 | 0.527504 | 0.125177 | 0.578093 | 2.277839 | 0.108293 | 0.344413 | 1.411942 | 2.1664 | 3.991742 | 0.445789 | high |
| TCGA-31-1951 | 0.827991 | 4.090914 | 2.083294 | 2.379079 | 3.642129 | 1.020568 | 2.35633 | 1.064873 | 5.364816 | 0.130915 | 0.223588 | 1.232231 | 3.157632 | 5.228034 | 1.720197 | high |
| TCGA-23-1123 | 0.753945 | 5.551939 | 2.234829 | 1.579562 | 4.688602 | 0.219851 | 0.467653 | 0.720247 | 5.343396 | 0.205633 | 0.180894 | 1.229006 | 3.873135 | 3.34239 | 1.809784 | low |
| TCGA-13-1501 | 0.118361 | 1.123674 | 0.313163 | 0.412803 | 2.098578 | 0.137453 | 0.079259 | 0.068176 | 2.06151 | 0.059484 | 0 | 0.285984 | 1.987983 | 2.474857 | 0.263661 | low |
| TCGA-13-1488 | 0.232256 | 3.086916 | 1.11525 | 1.473084 | 2.691426 | 0.09565 | 0.244399 | 0.751049 | 6.560569 | 0.322114 | 0.207291 | 1.04688 | 2.89433 | 3.647627 | 1.447827 | low |
| TCGA-36-1574 | 0.650638 | 2.906245 | 1.695937 | 1.831837 | 3.60585 | 0.17028 | 0.739133 | 0.895351 | 5.625247 | 0.306741 | 0.281728 | 1.219344 | 2.050777 | 3.334029 | 0.763784 | low |
| TCGA-29-1711 | 1.435724 | 4.370389 | 1.861904 | 1.376186 | 4.142191 | 0.705269 | 1.202588 | 1.124021 | 6.237002 | 0.223913 | 0.127215 | 1.885235 | 3.068685 | 4.662942 | 1.797933 | high |
| TCGA-59-2354 | 1.475669 | 3.599498 | 1.481945 | 1.556306 | 4.055633 | 0.795478 | 0.076182 | 2.295071 | 4.632939 | 0.313238 | 0.227963 | 1.684547 | 3.574838 | 3.87395 | 1.557879 | high |
| TCGA-23-2084 | 0.255858 | 3.522277 | 1.007917 | 1.032817 | 3.447688 | 0.327206 | 0.4793 | 0.718982 | 5.633769 | 0 | 0 | 0.838028 | 1.655784 | 4.112468 | 0.4815 | low |
| TCGA-13-0901 | 0.151402 | 1.971687 | 0.273998 | 0.334511 | 2.816963 | 0 | 0.262756 | 0.129527 | 1.199484 | 0.091352 | 0.215997 | 0.389037 | 1.345609 | 2.860592 | 0.125419 | high |
| TCGA-25-2404 | 0.399457 | 3.145527 | 0.944185 | 1.132268 | 2.895436 | 0.392843 | 0.218427 | 0.609032 | 4.538837 | 0.170749 | 0.154302 | 3.196387 | 2.370771 | 3.390207 | 0.919147 | low |
| TCGA-13-1489 | 0.25902 | 1.893262 | 0.397611 | 0.420924 | 2.252296 | 0.096246 | 0.042169 | 0.018832 | 3.601293 | 0.094375 | 0.35268 | 5.006712 | 2.035815 | 2.528124 | 0.440848 | low |
| TCGA-61-1721 | 1.524415 | 3.529554 | 0.659173 | 0.537672 | 3.940084 | 0.470696 | 0.088093 | 0.590517 | 1.220961 | 0.025169 | 0.935914 | 0.369218 | 2.17984 | 2.694647 | 0.417209 | low |
| TCGA-29-1763 | 0.340654 | 4.574117 | 0.632584 | 0.491323 | 2.82848 | 0.502388 | 0.389594 | 0.394597 | 5.305643 | 0.20797 | 0.514182 | 0.757899 | 1.922997 | 3.725376 | 0.520563 | low |
| TCGA-24-1464 | 0.747612 | 3.713037 | 0.660505 | 0.274652 | 3.424878 | 0.279447 | 0.11732 | 0.656676 | 2.768199 | 0.354534 | 0.553876 | 1.883948 | 3.192256 | 3.932255 | 0.96752 | low |
| TCGA-24-2271 | 0.816621 | 3.877774 | 1.910946 | 2.288025 | 3.445402 | 0.940636 | 0.851639 | 2.005759 | 3.273593 | 0.425097 | 0.074387 | 1.244271 | 3.593731 | 3.844242 | 1.598221 | low |
| TCGA-13-1404 | 0.195797 | 1.545705 | 0.497888 | 0.854428 | 2.439266 | 0.304866 | 0.157321 | 0.269833 | 1.800456 | 0.020502 | 0.098585 | 0.492223 | 2.655697 | 2.59645 | 0.635952 | low |
| TCGA-29-1762 | 0.311178 | 1.636823 | 0.015546 | 0.112502 | 2.499871 | 0.221536 | 0.217275 | 0.268267 | 0.357493 | 0.022618 | 0.413426 | 0.177514 | 0.383883 | 3.50637 | 0.006771 | low |
| TCGA-61-2113 | 0.587252 | 4.54089 | 2.058472 | 1.818679 | 3.305213 | 0.602242 | 1.276042 | 0.423522 | 5.101544 | 0.092146 | 0 | 2.065367 | 2.863411 | 4.566854 | 1.317541 | high |
| TCGA-20-0991 | 1.355396 | 5.024151 | 1.931074 | 3.241445 | 5.019107 | 0.122385 | 0.651468 | 0.76251 | 6.660921 | 0.045876 | 0.056464 | 1.832899 | 5.280119 | 3.55124 | 1.767627 | low |
| TCGA-25-2400 | 0.234865 | 2.253531 | 0.131568 | 0.440834 | 2.252296 | 0.341552 | 0.05293 | 0.641863 | 3.094799 | 1.189809 | 0.632759 | 0.411107 | 1.027775 | 4.101532 | 0.211089 | high |
| TCGA-25-1319 | 0.625231 | 2.961003 | 0.896599 | 0.579688 | 3.069165 | 0.426925 | 0.259084 | 0.3662 | 4.08669 | 0.929687 | 0.423909 | 0.920374 | 1.906074 | 3.80567 | 0.614789 | low |
| TCGA-24-1104 | 1.065408 | 2.427638 | 0.682508 | 0.811251 | 3.285267 | 0.74683 | 0.648755 | 1.834563 | 5.857982 | 0.215858 | 0.037852 | 0.755128 | 3.063883 | 3.893849 | 0.947868 | high |
| TCGA-23-1120 | 0.519571 | 4.831838 | 2.003728 | 2.072099 | 3.825383 | 0.290179 | 0.455124 | 0.813212 | 5.497909 | 0.12143 | 0.254415 | 0.759567 | 2.502903 | 3.746241 | 1.383692 | high |
| TCGA-04-1536 | 0.220042 | 2.061151 | 0.122987 | 0.122764 | 2.417436 | 0.206778 | 0.712367 | 0.334692 | 3.112835 | 0 | 0 | 4.166831 | 1.116702 | 3.286706 | 0.178089 | high |
| TCGA-61-1910 | 0.251961 | 2.400967 | 0.168221 | 0.38802 | 2.813119 | 1.030985 | 0.265043 | 0.119556 | 4.054412 | 0.042732 | 0.199816 | 0.099192 | 1.513973 | 5.099484 | 0.343147 | high |
| TCGA-13-1505 | 0.527828 | 3.268569 | 0.838636 | 1.246012 | 3.422381 | 0.442849 | 0.375971 | 0.685935 | 1.578033 | 0.539907 | 0.104015 | 0.515836 | 3.454561 | 4.791537 | 1.038475 | low |
| TCGA-25-1632 | 0.07213 | 0.946081 | 0.052302 | 0.096384 | 0.688331 | 0.288432 | 1.587236 | 0.054762 | 1.346034 | 0.199215 | 0.873003 | 0.173099 | 1.393672 | 2.729569 | 0.153443 | low |
| TCGA-24-0979 | 0.249388 | 2.083293 | 0.323989 | 0.238186 | 1.655524 | 0.309858 | 1.340772 | 0.434893 | 4.267071 | 0.132485 | 0.473895 | 0.295965 | 0.855749 | 3.967396 | 0.260447 | high |
| TCGA-61-2104 | 0.488545 | 4.079694 | 1.394222 | 1.255816 | 3.789773 | 0.679344 | 1.940953 | 0.558854 | 5.767865 | 0.729837 | 1.032684 | 1.060496 | 3.008497 | 4.94089 | 1.279957 | low |
| TCGA-20-0987 | 0.697105 | 4.754752 | 0.768076 | 2.1726 | 4.470444 | 0.376266 | 0.923157 | 2.880175 | 5.119833 | 0.065718 | 0 | 1.315898 | 3.832624 | 3.177342 | 0.806786 | low |
| TCGA-04-1347 | 0.022344 | 1.626016 | 0.182529 | 0.144268 | 1.180567 | 0.153538 | 0.136153 | 0.098512 | 1.112576 | 0.069259 | 1.31523 | 0.267534 | 0.890553 | 2.539601 | 0.159804 | low |
| TCGA-31-1944 | 0.531435 | 2.095634 | 1.32699 | 1.274195 | 2.647157 | 0.196164 | 0.484774 | 0.544283 | 2.988184 | 0 | 0.055749 | 0.715272 | 2.279706 | 3.978363 | 0.650514 | low |
| TCGA-29-1766 | 0.593427 | 3.094192 | 0.972191 | 0.662533 | 3.671781 | 0.329134 | 0.099901 | 0.365109 | 3.82281 | 1.05809 | 0.481411 | 0.464209 | 2.386018 | 3.661126 | 1.455369 | low |
| TCGA-24-1425 | 0.657075 | 2.222606 | 1.329579 | 1.552117 | 3.036663 | 0.80495 | 1.092379 | 1.609879 | 5.387718 | 0.055126 | 0.214608 | 0.965594 | 2.417283 | 3.657902 | 1.166527 | low |
| TCGA-61-1900 | 0.311592 | 1.955992 | 0.350345 | 0.641105 | 1.855172 | 0.288802 | 0.348971 | 0.203641 | 1.610429 | 0.030124 | 0.677626 | 0.23246 | 2.004423 | 4.301011 | 0.344975 | low |
| TCGA-04-1362 | 0.403728 | 1.766627 | 0.101203 | 0.326373 | 2.339853 | 0.642903 | 0.197789 | 0.739962 | 5.395122 | 0.212855 | 0.450597 | 0.434338 | 1.512936 | 5.3819 | 0.280456 | low |
| TCGA-24-1424 | 0.438354 | 3.314628 | 0.543267 | 1.047818 | 2.837748 | 0.316872 | 0.69223 | 0.245487 | 2.573663 | 0.161341 | 0.757883 | 0.569367 | 2.183271 | 4.236087 | 0.644942 | high |
| TCGA-24-1474 | 0.58862 | 2.289704 | 0.376764 | 0.709422 | 3.512167 | 0.275565 | 0.714773 | 0.41265 | 2.095872 | 0.123814 | 1.0967 | 0.241043 | 1.106051 | 2.04566 | 0.314912 | high |
| TCGA-24-1427 | 0.766966 | 2.49368 | 1.181656 | 1.400738 | 3.61872 | 0.455126 | 1.58386 | 0.256423 | 1.836707 | 0.048013 | 0.322882 | 0.8055 | 3.312803 | 2.943634 | 1.347602 | high |
| TCGA-10-0938 | 0 | 2.837118 | 0.040891 | 0.05084 | 0.68155 | 0.252765 | 0.270558 | 0.164152 | 1.609749 | 0.087999 | 0.208387 | 0.994718 | 1.446644 | 4.235409 | 0.137336 | high |
| TCGA-25-1312 | 0.232586 | 2.467347 | 0.249701 | 0.359415 | 2.682424 | 0.309338 | 0.566275 | 0.161967 | 1.86849 | 0.096127 | 0.347519 | 0.13471 | 1.543065 | 5.026142 | 0.336372 | low |
| TCGA-61-2102 | 0.245957 | 1.570197 | 0.047487 | 0.059009 | 3.824319 | 0.41612 | 0.668424 | 0.506709 | 2.749947 | 0.423999 | 2.244321 | 0.548131 | 1.213022 | 3.722341 | 0.04133 | high |
| TCGA-WR-A838 | 0.095405 | 2.057867 | 0.052308 | 0.096395 | 0.425492 | 0.528748 | 0.20229 | 0.389058 | 2.66917 | 0.281437 | 0.137226 | 0.045266 | 2.158045 | 4.062058 | 0.15346 | high |
| TCGA-24-2280 | 0.527446 | 2.530988 | 0.744504 | 0.842446 | 2.736613 | 0.732549 | 0.891563 | 0.22223 | 0.800051 | 0.139344 | 2.344896 | 0.662696 | 3.047268 | 3.516862 | 0.856675 | high |
| TCGA-61-1998 | 0.788172 | 2.360759 | 0.386514 | 0.785792 | 3.614945 | 0.184505 | 1.317231 | 0.585757 | 2.34204 | 0.035475 | 0.167375 | 0.372602 | 1.696566 | 3.385239 | 0.358005 | low |
| TCGA-29-1784 | 0.750914 | 3.292884 | 1.592255 | 2.154108 | 4.57115 | 0.22242 | 0.207449 | 0.757138 | 4.74301 | 0 | 0.0669 | 1.423726 | 3.73384 | 4.108873 | 1.274087 | high |
| TCGA-24-2038 | 0.282119 | 0.621469 | 0.06579 | 0.027726 | 1.817832 | 0.195386 | 0.11933 | 0.574294 | 1.05996 | 1.878402 | 0.356396 | 0.786594 | 0.884464 | 3.099314 | 0.139215 | high |
| TCGA-23-1107 | 0.118929 | 2.279762 | 0.12805 | 0.254595 | 1.999768 | 0.220157 | 0.265204 | 0.40514 | 4.385414 | 0 | 0 | 0.617444 | 1.964162 | 2.848643 | 0.34335 | low |
| TCGA-30-1718 | 0.345559 | 2.857898 | 1.453858 | 1.308685 | 4.057138 | 0.433487 | 0.137647 | 0.845197 | 5.124475 | 0.074912 | 0 | 0.854034 | 2.958397 | 2.474785 | 0.974374 | low |
| TCGA-36-1570 | 0.331543 | 2.292044 | 0.451921 | 0.288732 | 2.395912 | 0.325931 | 0.876316 | 0.180058 | 3.34877 | 0.07793 | 0.126207 | 0.205771 | 1.348627 | 3.900398 | 0.227444 | high |
| TCGA-24-1850 | 0.637425 | 2.842934 | 0.519431 | 1.390747 | 3.389616 | 0.485117 | 0.162984 | 0 | 4.383673 | 2.296946 | 0.378882 | 1.672283 | 3.258166 | 3.182631 | 0.924146 | high |
| TCGA-25-1326 | 1.056873 | 2.838672 | 1.103706 | 1.360654 | 3.867 | 0.516537 | 0.767795 | 0.610524 | 3.642684 | 0.518103 | 0.090445 | 1.163439 | 3.031005 | 4.517396 | 0.952766 | high |
| TCGA-31-1950 | 0.296908 | 4.05376 | 1.241713 | 1.086175 | 3.139462 | 0.399595 | 1.319815 | 0.383278 | 5.978154 | 0.212658 | 0.274149 | 0.889305 | 3.335835 | 5.491214 | 0.907949 | high |
| TCGA-61-1738 | 0.147426 | 3.393048 | 0.763234 | 0.545128 | 2.981991 | 0.286081 | 0.37507 | 0.572546 | 7.995271 | 0.405857 | 0 | 0.795518 | 1.868849 | 1.96453 | 0.489264 | low |
| TCGA-13-1496 | 0.480893 | 4.607078 | 1.45941 | 1.512623 | 4.087053 | 0.0575 | 0.779666 | 0.986154 | 5.689844 | 0.142575 | 0 | 1.635934 | 3.202701 | 3.477401 | 1.206493 | low |
| TCGA-10-0927 | 0.283525 | 3.147295 | 0.047553 | 0 | 1.612347 | 0.290666 | 0.191661 | 0.189624 | 0.816431 | 0.10207 | 1.018934 | 0.658604 | 1.326924 | 2.961015 | 0.041388 | high |
| TCGA-24-1844 | 0.244805 | 1.252853 | 0.043669 | 0.027396 | 1.294198 | 0.180215 | 0.736837 | 0 | 2.888712 | 0.093876 | 0.151525 | 0.037774 | 1.57961 | 4.338572 | 0.028594 | low |
| TCGA-24-2262 | 0.802732 | 2.945144 | 0.898113 | 2.19121 | 3.528294 | 0.561755 | 1.00727 | 0.857109 | 4.707136 | 0.148721 | 0 | 1.255936 | 3.352986 | 4.763184 | 1.641937 | low |
| TCGA-24-1103 | 0.197319 | 1.514705 | 0.297103 | 0.286248 | 1.502434 | 0.174895 | 0.469233 | 0.331213 | 6.541104 | 0.054478 | 0 | 0.165402 | 1.83746 | 3.976082 | 0.214634 | low |
| TCGA-61-1914 | 0.313923 | 2.002804 | 1.002834 | 1.121326 | 4.062602 | 0.100323 | 0.612508 | 0.133713 | 5.691016 | 0.094345 | 0 | 4.447987 | 2.181021 | 2.452362 | 0.535425 | low |
| TCGA-09-1659 | 0.228875 | 3.342618 | 0.209615 | 0.77168 | 3.145451 | 0.249496 | 0.80397 | 0.596566 | 4.33574 | 0.361881 | 0.025185 | 0.972205 | 1.842032 | 2.408325 | 0.312572 | low |
| TCGA-25-1318 | 0.150056 | 2.170317 | 0.520172 | 0.479144 | 2.609144 | 1.091099 | 0.705494 | 0.114655 | 2.587296 | 0.040936 | 0.050403 | 0.966382 | 1.244659 | 3.34746 | 0.531657 | high |
| TCGA-09-1670 | 0.494253 | 2.203196 | 0.237924 | 0.408102 | 3.511823 | 0.480285 | 1.416187 | 0.366321 | 3.647706 | 0.62585 | 0.103732 | 0.85567 | 1.981637 | 3.549345 | 0.486734 | high |
| TCGA-29-1769 | 0.458227 | 2.990925 | 0.878684 | 0.796059 | 3.727528 | 0.195136 | 0.443231 | 0.635415 | 3.342815 | 0.016249 | 0 | 1.176519 | 1.968467 | 3.641684 | 0.577583 | low |
| TCGA-25-1623 | 1.124692 | 4.848895 | 3.233251 | 3.303761 | 4.664034 | 0.351967 | 1.243615 | 1.292195 | 4.459262 | 0.063248 | 0.077739 | 1.654682 | 4.419857 | 3.913042 | 2.42668 | low |
| TCGA-24-1930 | 0.990907 | 4.723651 | 1.660155 | 1.723841 | 4.464519 | 0.457782 | 0.081307 | 0.544532 | 3.818608 | 0.554518 | 0.035114 | 1.482452 | 3.493187 | 3.457881 | 1.553736 | high |
| TCGA-61-1741 | 0.32207 | 3.831746 | 1.896269 | 1.993432 | 3.174001 | 0.501089 | 0.579945 | 0.334962 | 3.184466 | 0.259451 | 0.924999 | 0.718837 | 3.87616 | 4.652232 | 1.13176 | high |
| TCGA-59-2355 | 0.423717 | 2.924314 | 0.627696 | 0.848076 | 3.104821 | 0.173952 | 0.416451 | 1.208181 | 4.530218 | 0.470741 | 0.08103 | 2.875512 | 2.232408 | 2.79083 | 0.848497 | low |
| TCGA-24-2023 | 0.457416 | 2.412767 | 0.764264 | 0.696077 | 2.639393 | 0.170261 | 0.109504 | 0.883311 | 6.282124 | 0.068986 | 0 | 0.659735 | 1.990376 | 4.173077 | 0.662948 | high |
| TCGA-24-2024 | 0.497888 | 1.907732 | 0.156363 | 0.344575 | 2.177783 | 0.183396 | 0.675686 | 0.56545 | 3.195428 | 0.115674 | 0.270537 | 0.362308 | 2.867713 | 3.95607 | 0.313858 | high |
| TCGA-09-0364 | 0.084761 | 1.293959 | 0 | 0.076375 | 0.377653 | 0.334817 | 0.483282 | 0.064419 | 0.145391 | 1.045811 | 1.148562 | 0 | 1.375151 | 3.127073 | 0.05359 | high |
| TCGA-13-1512 | 0.279184 | 3.234826 | 0.680921 | 0.772531 | 3.726767 | 0.145804 | 0.684823 | 0.200033 | 4.498116 | 0 | 0.291303 | 1.113617 | 2.65335 | 3.207828 | 0.74667 | low |
| TCGA-13-1497 | 0.251948 | 2.959544 | 0.842154 | 0.742044 | 3.195195 | 0.185593 | 0.141977 | 0.301709 | 4.696098 | 0.016585 | 0.040635 | 0.577934 | 3.27123 | 2.948901 | 0.737966 | low |
| TCGA-61-2097 | 0.298995 | 2.365146 | 0.476003 | 1.135563 | 3.051692 | 0.306485 | 2.338079 | 0.456686 | 3.536882 | 0.208497 | 0.089533 | 0.634278 | 1.485328 | 4.680156 | 0.226189 | high |
| TCGA-13-0891 | 0 | 2.953419 | 0.338695 | 0.623147 | 1.538311 | 0.214411 | 0.160511 | 0.488867 | 3.64747 | 0.025089 | 0 | 0.413873 | 1.089596 | 3.438463 | 0.23583 | low |
| TCGA-20-1682 | 0.534807 | 2.736305 | 1.280905 | 1.311296 | 3.756104 | 0.331462 | 1.300002 | 0.491969 | 4.403867 | 0.074536 | 0 | 5.972747 | 2.975449 | 2.501562 | 1.178186 | low |
| TCGA-24-1430 | 0.107396 | 0.889509 | 0.093264 | 0.142934 | 0.979015 | 0.289725 | 0.284277 | 0.049619 | 0.825271 | 0.25677 | 0.994705 | 0.119652 | 1.770413 | 3.223482 | 0.061429 | high |
| TCGA-10-0936 | 0.449302 | 4.414435 | 0.922645 | 1.494721 | 2.328354 | 0.437094 | 2.193569 | 1.122427 | 5.472888 | 1.909329 | 0.038928 | 2.970874 | 2.937934 | 5.321627 | 0.797646 | high |
| TCGA-13-1408 | 0.263807 | 2.49154 | 1.005593 | 1.24214 | 2.45246 | 0.373987 | 0.409988 | 0.648728 | 3.739602 | 0.055906 | 0.108488 | 0.966539 | 2.823328 | 4.099727 | 0.899818 | high |
| TCGA-13-0906 | 0.357556 | 2.757967 | 0.862283 | 0.714956 | 2.472465 | 0.169315 | 0.9197 | 0.526876 | 4.66429 | 0.205864 | 0.116645 | 0.947082 | 2.213661 | 3.102374 | 0.973832 | low |
| TCGA-25-1633 | 1.679396 | 3.636626 | 2.613576 | 2.17805 | 4.245693 | 0.243628 | 0.352942 | 1.167865 | 2.902913 | 1.173106 | 0.106428 | 0.853574 | 3.240179 | 4.086791 | 2.385551 | low |
| TCGA-09-1666 | 0.643024 | 3.770374 | 1.356064 | 1.186378 | 3.435015 | 0.416608 | 1.000367 | 0.521959 | 6.00743 | 0.339621 | 0.044363 | 1.054668 | 2.647778 | 3.189056 | 1.289861 | high |
| TCGA-24-1418 | 0.291494 | 1.980639 | 0.802537 | 0.981761 | 2.84344 | 0.347802 | 0.285026 | 0.514917 | 2.651095 | 0.346353 | 0.34274 | 0.503411 | 2.232452 | 3.928831 | 0.808449 | low |
| TCGA-29-1761 | 0.826166 | 2.250897 | 1.243263 | 1.019051 | 4.248432 | 0.133191 | 0.442675 | 0.383308 | 3.846701 | 0.07443 | 0 | 0.826171 | 2.161216 | 2.772417 | 0.446322 | high |
| TCGA-24-1847 | 2.581558 | 4.728676 | 2.472959 | 1.913304 | 4.837269 | 0.496629 | 0.719931 | 1.529593 | 5.603792 | 0.299857 | 0 | 2.210108 | 3.9927 | 4.643076 | 1.655526 | low |
| TCGA-24-2033 | 0.110298 | 2.674714 | 0.147012 | 0.146749 | 1.922427 | 0.346556 | 0.498598 | 0.535722 | 0.373881 | 0.96673 | 0.054719 | 0.28954 | 2.021038 | 4.285104 | 0.188718 | high |
| TCGA-09-2056 | 0.573335 | 1.893708 | 0.438789 | 0.646934 | 3.759851 | 0.177128 | 0.116299 | 0.760122 | 3.441283 | 0.038178 | 0 | 6.50046 | 2.593496 | 2.330519 | 0.479111 | high |
| TCGA-09-2053 | 0.418593 | 4.216921 | 0.926839 | 0.670581 | 3.326189 | 0.549244 | 1.248917 | 0.789885 | 3.957991 | 0.272042 | 0.513248 | 1.101885 | 3.909497 | 4.817279 | 1.31597 | high |
| TCGA-29-1694 | 0.335045 | 2.19161 | 0.770553 | 1.089055 | 2.255621 | 0.271292 | 0.163138 | 0.87069 | 4.218194 | 0.127849 | 0.071593 | 1.285792 | 2.134233 | 3.788635 | 0.569823 | high |
| TCGA-24-1417 | 1.107766 | 4.964445 | 2.635584 | 2.479517 | 4.813036 | 0.685083 | 0.402609 | 2.709798 | 6.435758 | 0.038662 | 0.223752 | 1.671885 | 3.130145 | 3.569805 | 1.9098 | low |
| TCGA-29-1776 | 0.66662 | 2.075935 | 0.930794 | 1.147681 | 3.218857 | 0.144888 | 0.453635 | 0.361692 | 3.823613 | 0.092335 | 0 | 0.392816 | 2.344939 | 1.74658 | 0.614774 | high |
| TCGA-61-2008 | 1.1754 | 3.603548 | 1.708885 | 1.89593 | 3.414757 | 0.90227 | 1.174897 | 0.448353 | 3.652487 | 0.057885 | 0.57091 | 1.725572 | 3.592527 | 5.256935 | 1.481736 | low |
| TCGA-09-0367 | 0.177106 | 1.475474 | 0.290874 | 0.597787 | 2.750047 | 0.209915 | 1.022512 | 0.971589 | 5.677198 | 0.118675 | 0 | 0.667801 | 1.813206 | 2.412954 | 0.327997 | low |
| TCGA-29-1783 | 0.345156 | 2.975723 | 0.857892 | 1.351644 | 3.393179 | 0.42029 | 1.365859 | 0.278381 | 2.987147 | 0.139895 | 0.064527 | 0.728977 | 2.579335 | 4.34022 | 0.829616 | high |
| TCGA-13-1509 | 0.178911 | 1.802245 | 0.804027 | 0.763858 | 2.997054 | 0.311654 | 0.280562 | 0.243591 | 1.313794 | 0.400537 | 0.028238 | 1.252545 | 3.089366 | 2.151762 | 0.607874 | low |
| TCGA-13-1411 | 1.248047 | 3.810579 | 1.145419 | 1.176466 | 5.055175 | 0.202123 | 0.207925 | 0.845586 | 3.023711 | 0.061195 | 0 | 0.848116 | 3.531859 | 2.853063 | 1.563529 | low |
| TCGA-61-2110 | 0.267234 | 1.048453 | 0.161383 | 0.14467 | 2.195004 | 0.444025 | 0.602518 | 0.191249 | 1.730199 | 0.467014 | 0.043296 | 1.076771 | 2.145863 | 4.331347 | 0.179083 | high |
| TCGA-25-1329 | 0.316122 | 2.522088 | 0.353002 | 0.377071 | 2.757408 | 0.435638 | 0.151806 | 0.212205 | 5.447231 | 0.026273 | 0.125406 | 3.85897 | 2.387118 | 4.128112 | 0.484853 | high |
| TCGA-36-1569 | 1.362217 | 2.837224 | 1.786291 | 1.632177 | 4.552273 | 0.399434 | 0.301067 | 1.083006 | 3.763999 | 0.150251 | 0.289107 | 1.244998 | 4.229756 | 2.568693 | 1.709194 | high |
| TCGA-09-2051 | 0.603322 | 4.578726 | 1.498592 | 1.280478 | 3.807805 | 0.818037 | 1.176897 | 1.336598 | 6.033786 | 0.097091 | 0.090174 | 1.006815 | 2.237484 | 5.037174 | 0.854663 | low |
| TCGA-13-2060 | 0.564528 | 3.3788 | 1.728324 | 2.213901 | 4.183406 | 0.275676 | 0.153769 | 0.567404 | 5.474838 | 0.029923 | 0.072833 | 0.994457 | 3.597576 | 2.794242 | 2.033833 | low |
| TCGA-13-0924 | 0.927119 | 2.925676 | 1.744678 | 1.94282 | 4.209322 | 0.192032 | 0.25537 | 1.401964 | 4.380028 | 0.049127 | 0.118469 | 3.324923 | 3.866727 | 2.213969 | 1.044223 | low |
| TCGA-13-0726 | 0.257106 | 2.061943 | 0.184401 | 0.53453 | 2.061545 | 0.356938 | 0.628296 | 1.609893 | 2.593794 | 0 | 0.031865 | 0.25411 | 1.702836 | 2.789933 | 0.527435 | low |
| TCGA-24-2020 | 0.277208 | 1.420952 | 0.147546 | 0.093797 | 1.499222 | 0.73419 | 0.376868 | 0 | 0.533691 | 0.316611 | 0 | 0.033144 | 0.792653 | 3.257003 | 0.113477 | low |
| TCGA-57-1584 | 0.458694 | 1.931795 | 0.150901 | 0.242968 | 2.810001 | 0.287273 | 0.23855 | 0.622208 | 4.170521 | 0.03816 | 0.092509 | 0.46544 | 1.680911 | 2.795473 | 0.25281 | high |
| TCGA-30-1857 | 0.365458 | 1.750351 | 0.452084 | 0.885702 | 3.497174 | 0.283143 | 0.698845 | 1.089028 | 3.595782 | 0.283051 | 0.036332 | 1.41238 | 2.699615 | 3.527341 | 0.83237 | low |
| TCGA-24-1553 | 1.475476 | 4.267999 | 2.624138 | 2.305608 | 5.475014 | 0.193267 | 0.550911 | 1.664823 | 5.249019 | 0.210322 | 0.447349 | 2.258627 | 5.101483 | 3.398308 | 2.604474 | high |
| TCGA-13-1507 | 0.905689 | 3.657424 | 2.323517 | 2.212414 | 4.933178 | 0.179252 | 0.105107 | 2.018902 | 4.80586 | 0.11702 | 0 | 1.137644 | 3.620806 | 2.154689 | 1.255315 | low |
| TCGA-23-1122 | 0.223983 | 3.048077 | 0.431757 | 0.431053 | 2.778728 | 0.211657 | 1.083805 | 0.495155 | 3.703989 | 0.395588 | 0.487795 | 0.393102 | 1.746753 | 5.066583 | 0.368351 | low |
| TCGA-04-1365 | 0.817862 | 4.687678 | 1.564053 | 1.770987 | 4.220997 | 0.408991 | 0.264882 | 0.91641 | 4.395024 | 0.397384 | 0.029444 | 1.31138 | 3.30869 | 3.859142 | 1.776111 | high |
| TCGA-61-1724 | 0.402118 | 2.290326 | 0.588283 | 1.16002 | 2.60006 | 0.340233 | 0.190994 | 0.848385 | 3.375464 | 0 | 0.087544 | 1.213386 | 2.778621 | 5.031842 | 0.816736 | high |
| TCGA-24-1604 | 0.281038 | 3.521111 | 1.223896 | 0.916571 | 2.853698 | 0.481824 | 0.086184 | 0.416554 | 4.25643 | 0.172562 | 0.073574 | 0.263683 | 2.805706 | 3.602243 | 0.738398 | low |
| TCGA-61-1919 | 0.554304 | 2.531322 | 1.138663 | 1.227463 | 4.404917 | 0.135277 | 0.766702 | 0.234814 | 2.467739 | 0.043695 | 0.105646 | 0.848655 | 3.33014 | 2.152012 | 0.923903 | low |
| TCGA-23-1110 | 0.145362 | 2.008165 | 0 | 0 | 0.502104 | 0.106039 | 0.675828 | 0 | 0.839505 | 0 | 0.141636 | 0.758665 | 0.894675 | 2.62741 | 0.035429 | high |
| TCGA-57-1582 | 0.483084 | 1.212193 | 0.975715 | 0.510283 | 2.883118 | 0.131437 | 0.382475 | 0.292901 | 1.178331 | 0.07343 | 0 | 1.449257 | 2.145694 | 3.581657 | 0.521349 | high |
| TCGA-24-1845 | 0.173693 | 2.758923 | 0.387676 | 0.441592 | 2.816819 | 0.481839 | 0.425714 | 0.062896 | 6.500268 | 0.086809 | 0.054234 | 0.265304 | 1.46198 | 4.213644 | 0.261229 | high |
| TCGA-59-2348 | 0.302355 | 2.449437 | 1.098083 | 0.916232 | 3.004559 | 0.306635 | 0.219429 | 0.48917 | 3.446401 | 0.015502 | 0.038003 | 0.173619 | 2.944366 | 4.598857 | 0.793579 | low |
| TCGA-13-0884 | 0.977025 | 3.183235 | 1.134519 | 1.219317 | 3.678146 | 1.049619 | 0.249568 | 2.449685 | 4.579396 | 0.038778 | 0.047755 | 0.77356 | 3.081243 | 3.449435 | 1.205341 | low |
| TCGA-61-1736 | 0.589067 | 1.728132 | 0.422698 | 0.748101 | 2.910383 | 1.085722 | 0.637048 | 0.746596 | 5.611989 | 0.389926 | 0.126982 | 0.770033 | 1.893014 | 4.831637 | 0.390059 | high |
| TCGA-57-1583 | 0.087411 | 2.443077 | 0.344686 | 0.160723 | 1.49596 | 0.147769 | 0.582359 | 0.117487 | 3.185728 | 0.678306 | 0.034656 | 0.24656 | 1.211797 | 3.964545 | 0.129288 | high |
| TCGA-04-1514 | 0.087535 | 2.464025 | 0.071316 | 0.116762 | 0.641086 | 0.407389 | 0.829261 | 0.050192 | 2.329995 | 0.119327 | 0.512063 | 0.159182 | 1.076584 | 4.661096 | 0.102122 | low |
| TCGA-10-0937 | 0.151115 | 3.008252 | 0.674814 | 1.27128 | 3.154085 | 0.110295 | 0.04149 | 0.954176 | 3.432371 | 0.06142 | 0 | 1.021245 | 2.047026 | 2.300275 | 1.016478 | low |
| TCGA-29-2427 | 1.741976 | 4.287293 | 1.532417 | 1.101636 | 5.135733 | 0.960153 | 0.08888 | 2.832954 | 6.163611 | 0.19167 | 0 | 1.616532 | 3.791233 | 2.015794 | 1.278721 | low |
| TCGA-24-1846 | 0.350276 | 2.559128 | 1.588391 | 2.353036 | 3.538521 | 0.519123 | 0.551493 | 0.847892 | 3.773994 | 0.141535 | 0.106218 | 0.909606 | 2.368179 | 4.001755 | 1.439622 | high |
| TCGA-29-1690 | 1.095074 | 1.643627 | 1.700472 | 1.828662 | 3.924879 | 0.171868 | 1.150488 | 0.844938 | 3.96569 | 0.881074 | 0.14444 | 2.409686 | 2.932214 | 2.582797 | 1.136948 | high |
| TCGA-24-1423 | 0.453533 | 3.006515 | 1.159884 | 1.008031 | 2.338991 | 0.291884 | 0.144999 | 0.242364 | 3.906587 | 0.207365 | 0.34436 | 2.144964 | 2.294573 | 3.466031 | 0.70796 | low |
| TCGA-13-0900 | 0.134486 | 3.131568 | 0.971972 | 1.068482 | 3.399017 | 0.199621 | 0.156692 | 0.426602 | 5.249987 | 0.036543 | 0 | 0.404811 | 2.02059 | 2.721725 | 0.80192 | low |
| TCGA-29-1705 | 0.635704 | 1.43946 | 0.590782 | 0.467444 | 2.900544 | 0.230998 | 0.356235 | 0.33013 | 0.73052 | 0.209847 | 0.039322 | 0.686124 | 1.83199 | 3.562979 | 0.491819 | high |
| TCGA-29-2425 | 0.212534 | 2.31902 | 0.907864 | 0.816011 | 2.404269 | 0.139478 | 0.026642 | 0.110831 | 2.571501 | 0.28956 | 0.048686 | 1.037335 | 2.709027 | 3.280814 | 0.605154 | high |
| TCGA-23-1024 | 0.187293 | 2.349736 | 0.271817 | 0.442826 | 1.897806 | 0.106644 | 1.111548 | 0.311977 | 3.145292 | 0.314512 | 0 | 0.398546 | 1.71823 | 3.692438 | 0.485571 | high |
| TCGA-25-1320 | 0.292592 | 2.37182 | 0.632718 | 1.077933 | 2.516502 | 0.601835 | 0.12402 | 0.048144 | 2.774812 | 0.145696 | 0.861411 | 1.731235 | 2.450134 | 4.332683 | 0.731958 | high |
| TCGA-24-1552 | 0.200665 | 0.802445 | 0.363516 | 0.440974 | 1.467393 | 0.37655 | 1.10872 | 0.201819 | 1.092939 | 0.193669 | 0.04582 | 0.282676 | 1.14371 | 3.567726 | 0.921732 | high |
| TCGA-09-2048 | 0.091908 | 1.336328 | 0.20953 | 0.598504 | 2.151867 | 0.079689 | 0.04606 | 0.20027 | 0.54699 | 0.500303 | 0.106764 | 0.069108 | 1.378176 | 1.965955 | 1.15173 | low |
| TCGA-31-1946 | 0.748412 | 3.481641 | 1.670837 | 1.78051 | 3.641134 | 0.285521 | 0.688345 | 0.823463 | 4.394686 | 0.19023 | 0.538742 | 1.486412 | 2.514318 | 3.614371 | 1.243691 | low |
| TCGA-10-0933 | 0.075245 | 2.42079 | 0.176476 | 0.261132 | 1.177251 | 0.157768 | 0.481383 | 0.203525 | 0.807774 | 1.038671 | 0.073271 | 0.962386 | 0.183462 | 2.519474 | 0.053347 | high |
| TCGA-24-1416 | 0.303189 | 2.041107 | 0.030055 | 0.144121 | 2.276059 | 0.258584 | 0.286526 | 1.160518 | 3.865354 | 1.333313 | 0.339713 | 0.847664 | 1.610442 | 3.92303 | 0.219766 | low |
| TCGA-10-0931 | 0.254866 | 2.860847 | 0.308626 | 0.665297 | 2.418955 | 0.231154 | 0.048285 | 0.882545 | 4.460064 | 0.023337 | 0 | 0.345119 | 1.870558 | 4.366691 | 0.466707 | low |
| TCGA-36-1577 | 0.38665 | 1.567296 | 0.058661 | 0.024686 | 2.445665 | 0.255186 | 0.250332 | 0.041232 | 0.500939 | 0.08478 | 0.865903 | 1.210099 | 0.552947 | 3.754332 | 0.051082 | low |
| TCGA-25-1627 | 0.33255 | 1.683248 | 0.189044 | 0.258838 | 2.665489 | 0.113166 | 0.155951 | 0.489007 | 1.324623 | 0.036363 | 0 | 0.125183 | 1.971536 | 2.221123 | 0.464655 | high |
| TCGA-24-2281 | 0.664871 | 4.218795 | 1.652125 | 2.36232 | 3.923762 | 0.218841 | 0.903632 | 0.49053 | 3.707898 | 0.414728 | 0.044952 | 1.5413 | 3.377245 | 4.449336 | 1.836136 | low |
| TCGA-04-1341 | 0.054317 | 2.476204 | 0.155706 | 0.389822 | 1.560833 | 0.086932 | 0.438076 | 0.563374 | 2.353129 | 1.082849 | 0.334962 | 0.669291 | 2.893963 | 3.148711 | 0.313768 | high |
| TCGA-13-1410 | 0.354278 | 3.140523 | 1.38549 | 1.188264 | 3.318464 | 0.695091 | 0.630612 | 0.754821 | 4.819409 | 0.097291 | 0.035094 | 0.811494 | 2.584734 | 3.156364 | 0.815259 | low |
| TCGA-31-1956 | 1.640282 | 4.261713 | 2.381185 | 4.057915 | 5.347495 | 0.476056 | 0.236606 | 2.513081 | 4.883323 | 0.102719 | 0 | 1.989167 | 4.732742 | 3.170961 | 2.698856 | low |
| TCGA-61-2109 | 0.352242 | 2.72147 | 0.512709 | 0.370522 | 2.774715 | 0.526632 | 0.651324 | 0.740518 | 2.394309 | 0.320719 | 0 | 1.0739 | 2.227204 | 5.88346 | 0.800098 | low |
| TCGA-23-1023 | 1.091567 | 3.172581 | 0.312085 | 0.529169 | 3.054961 | 0.250046 | 2.213193 | 0.726854 | 7.673907 | 0.086999 | 0.036462 | 0.733108 | 1.915125 | 2.686541 | 0.486434 | low |
| TCGA-24-1549 | 0.880058 | 4.071951 | 1.92294 | 2.185062 | 3.135975 | 0.141015 | 0.277723 | 1.46418 | 5.235096 | 0.57255 | 0.637388 | 1.393132 | 2.892631 | 3.949919 | 1.519513 | high |
| TCGA-25-1328 | 0.635488 | 2.58161 | 0.626183 | 0.746797 | 2.268989 | 0.608712 | 0.846091 | 1.581764 | 2.337851 | 0.083315 | 0.31592 | 2.644906 | 2.778655 | 4.898189 | 0.679701 | high |
| TCGA-04-1350 | 0.098976 | 0.907915 | 0.106623 | 0.275651 | 1.171053 | 0.153677 | 0.131014 | 0 | 0.534012 | 0.181738 | 0 | 0.074468 | 1.384914 | 3.28865 | 0.205669 | low |
| TCGA-09-1669 | 0.47806 | 2.78477 | 1.846823 | 1.600781 | 3.537797 | 0.481008 | 0.383476 | 0.476472 | 3.518987 | 0.152387 | 0.043462 | 1.1576 | 3.657902 | 3.780584 | 1.496028 | low |
| TCGA-29-1696 | 0.278707 | 0.977449 | 0.40529 | 0.736183 | 2.651469 | 0.143444 | 0.753518 | 0.386271 | 4.714117 | 0.233269 | 0.2261 | 0.425702 | 1.270937 | 2.503089 | 0.368252 | high |
| TCGA-13-0724 | 0.230139 | 2.513263 | 0.097683 | 0.608252 | 2.364656 | 0.128719 | 0.175644 | 0.197644 | 2.884177 | 0 | 0.059457 | 0.569288 | 1.527862 | 4.687368 | 0.178648 | high |
| TCGA-24-1413 | 0.086823 | 2.618647 | 0.777932 | 0.898238 | 2.457502 | 0.42454 | 0.241347 | 0.458871 | 5.791301 | 2.351701 | 0.312013 | 0.548639 | 2.050372 | 3.781906 | 0.432037 | low |
| TCGA-29-1701 | 0.504226 | 2.723788 | 1.02693 | 1.117275 | 4.519269 | 0.329302 | 0.038847 | 0.935112 | 2.226169 | 0.21247 | 0.164302 | 1.306673 | 2.627702 | 2.781446 | 0.75741 | low |
| TCGA-09-2044 | 0.5147 | 3.967608 | 1.645038 | 1.321336 | 4.314468 | 0.137546 | 0.610261 | 0.557916 | 2.528812 | 0.051729 | 0.094436 | 1.993423 | 2.765276 | 2.51674 | 1.116662 | low |
| TCGA-36-1571 | 0.196636 | 2.075289 | 0.39556 | 0.215377 | 1.20001 | 0.168848 | 0.672571 | 0.345364 | 1.533925 | 0.087788 | 0.072683 | 0.136223 | 1.965315 | 3.994236 | 0.216475 | high |
| TCGA-24-1426 | 0.04383 | 0.846283 | 0.031696 | 0.096646 | 1.585406 | 0.217544 | 0.711515 | 0.328902 | 1.957069 | 0.587045 | 0.084111 | 0.343073 | 1.235266 | 3.636252 | 0.034378 | high |
| TCGA-24-1603 | 0.405633 | 1.242376 | 0.406693 | 0.507833 | 2.276505 | 0.212267 | 1.077976 | 0.211015 | 3.62495 | 0.039 | 0.154238 | 1.279283 | 1.537592 | 3.314077 | 0.322143 | low |
| TCGA-13-1407 | 0.056688 | 2.470594 | 0.015529 | 0.075885 | 0.965664 | 0.2302 | 0.040171 | 0.032357 | 0.405744 | 0.479845 | 0.475054 | 0.103982 | 0.981435 | 3.71211 | 0.225493 | low |
| TCGA-25-1635 | 0.742611 | 2.931736 | 1.244745 | 1.271185 | 4.05089 | 0.332357 | 0.06229 | 0.711909 | 2.85617 | 0.035151 | 0.085341 | 0.790695 | 3.090555 | 3.782759 | 1.154681 | low |
| TCGA-09-1661 | 0.074391 | 0.579863 | 0.053953 | 0.067008 | 2.570682 | 0.122784 | 0.061426 | 0.163236 | 0.62375 | 0.078031 | 0 | 0 | 1.280731 | 1.606486 | 0.046972 | low |
| TCGA-25-2392 | 0.667474 | 4.655306 | 1.022069 | 1.54614 | 4.860582 | 0.260048 | 0.71383 | 1.004506 | 1.954133 | 0.058235 | 0.071605 | 1.211953 | 3.091354 | 3.871059 | 0.963812 | high |
| TCGA-13-1506 | 0.285927 | 2.337101 | 0.83932 | 0.864366 | 2.172077 | 0.189261 | 1.069158 | 0.683918 | 5.362327 | 0.386204 | 0 | 1.094555 | 2.12291 | 2.68182 | 0.531593 | low |
| TCGA-23-1118 | 0.307724 | 2.919446 | 0.579049 | 1.323915 | 3.572918 | 0.306666 | 0.491488 | 0.499815 | 5.684377 | 0.138608 | 0.279245 | 0.690563 | 2.41568 | 4.407185 | 1.027142 | low |
| TCGA-24-1565 | 0.752637 | 3.078017 | 0.137906 | 0.044446 | 3.439074 | 0.701652 | 0.353967 | 1.693189 | 2.139767 | 3.391093 | 0.154397 | 0.899872 | 2.368248 | 3.438856 | 0.454432 | low |
| TCGA-23-1111 | 0.020144 | 1.45854 | 0.222885 | 0.295589 | 1.122941 | 0.571025 | 0.501902 | 0.045208 | 4.854326 | 0.07776 | 0.038949 | 0.143898 | 1.097352 | 5.283044 | 0.14473 | high |
| TCGA-24-1550 | 0.665427 | 3.3311 | 1.647529 | 1.451043 | 3.894759 | 0.29766 | 0.621586 | 0.489318 | 2.993647 | 0.084353 | 0.482031 | 0.83514 | 2.561864 | 2.403802 | 1.024176 | high |
| TCGA-24-2026 | 0.783452 | 3.524222 | 1.406859 | 1.888344 | 3.174871 | 0.457978 | 0.198327 | 1.008871 | 3.932447 | 0.198762 | 0.078811 | 0.937061 | 3.347337 | 1.751329 | 1.495727 | high |
| TCGA-13-0797 | 0.447327 | 3.173164 | 0.810552 | 1.159955 | 2.98694 | 0.49364 | 0.830737 | 1.291825 | 6.319767 | 0.621353 | 0 | 0.573464 | 3.26791 | 5.357342 | 0.851829 | high |
| TCGA-24-2261 | 1.29438 | 4.160838 | 2.005998 | 1.951705 | 4.445858 | 0.473947 | 1.457096 | 1.185683 | 3.288514 | 1.98004 | 0.031278 | 1.572455 | 4.302594 | 5.347094 | 2.129384 | high |
| TCGA-23-1116 | 0.664595 | 3.110825 | 1.392427 | 1.974107 | 4.100707 | 0.163858 | 0.26155 | 0.604446 | 4.151407 | 0.046731 | 0 | 1.310861 | 2.105258 | 3.718102 | 0.786882 | low |
| TCGA-31-1959 | 1.368274 | 3.852622 | 1.437432 | 1.467336 | 4.526043 | 0.49461 | 0.512567 | 1.22818 | 4.150395 | 0.055102 | 0 | 1.704089 | 3.942161 | 4.12538 | 1.332563 | low |
| TCGA-30-1866 | 0.723718 | 2.948374 | 0.768625 | 0.566988 | 3.549556 | 0.203124 | 0.072887 | 0.561427 | 3.978679 | 0.023685 | 0.166961 | 1.486803 | 2.650727 | 2.811897 | 0.862433 | high |
| TCGA-25-1322 | 0.276533 | 3.776503 | 0.469221 | 0.650268 | 2.796672 | 0.6041 | 0.578783 | 0.892137 | 4.345457 | 0.144192 | 0.176131 | 0.622157 | 1.49829 | 4.858351 | 0.369705 | low |
| TCGA-24-1842 | 1.202285 | 4.425144 | 1.812142 | 2.213264 | 4.217044 | 0.483695 | 0.791922 | 1.822949 | 4.232747 | 0 | 0 | 1.305245 | 3.767675 | 4.428857 | 2.256525 | high |
| TCGA-61-2088 | 0.034181 | 4.669828 | 0.107945 | 0.09034 | 1.003065 | 1.877234 | 0.172094 | 0 | 1.079487 | 0.130178 | 0.065794 | 0.123565 | 3.522731 | 7.433444 | 0.153752 | low |
| TCGA-24-1558 | 0.113173 | 2.236591 | 0.082381 | 0.102075 | 2.427466 | 0.435385 | 0.364548 | 0 | 0.641586 | 1.165271 | 0.074422 | 0.296634 | 1.392841 | 3.638645 | 0.071812 | high |
| TCGA-25-1323 | 0.085965 | 3.442632 | 0.092638 | 0.114689 | 1.945977 | 0.339182 | 0.387599 | 0.143062 | 2.102552 | 0.180145 | 0.123822 | 0.156395 | 1.317805 | 5.064313 | 0.147951 | high |
| TCGA-61-1737 | 0.372675 | 2.11059 | 0.635706 | 1.001214 | 3.609449 | 0.115009 | 0.124714 | 0.461694 | 5.151673 | 0.071039 | 0 | 2.181756 | 2.004378 | 3.461755 | 0.732239 | low |
| TCGA-09-2054 | 0.593704 | 2.803959 | 0.867804 | 0.79503 | 2.806873 | 0.504487 | 0.329086 | 0.2199 | 0.793092 | 0.040745 | 0.830671 | 0.492523 | 2.706284 | 3.405908 | 0.612377 | low |
| TCGA-13-0897 | 0.216121 | 2.966032 | 1.0702 | 1.544253 | 2.740546 | 0.128315 | 0.110635 | 1.017491 | 5.229934 | 0.071652 | 0.044676 | 1.458375 | 3.173545 | 0.886332 | 1.110878 | low |
| TCGA-59-2363 | 0.221437 | 1.680661 | 0.136914 | 0.168903 | 1.925539 | 0.563081 | 0.8365 | 0.05898 | 3.940782 | 0.177185 | 0.923595 | 0.663911 | 1.500856 | 3.582722 | 0.302687 | high |
| TCGA-61-2003 | 1.594024 | 3.354839 | 0.764271 | 0.553488 | 4.363579 | 0.398067 | 0.180599 | 0.550107 | 2.669396 | 0.017814 | 0.085959 | 0.667469 | 2.802478 | 3.804631 | 0.905343 | low |
| TCGA-25-2409 | 1.094581 | 2.621609 | 0.558636 | 1.074651 | 3.238851 | 0.974239 | 0.95591 | 1.401355 | 5.656554 | 0.356355 | 0 | 0.519767 | 2.936019 | 4.49111 | 0.834208 | high |
| TCGA-61-2111 | 0.932418 | 1.857137 | 0.899826 | 0.97983 | 3.425769 | 0.449171 | 0.166545 | 0.931569 | 2.894839 | 0.897579 | 0.142744 | 0.320942 | 3.264291 | 4.046906 | 1.019847 | low |
| TCGA-25-1315 | 0.083112 | 3.08856 | 0.106837 | 0.045396 | 0.972699 | 0.129432 | 0.625882 | 0.075464 | 1.478826 | 1.986047 | 0.352741 | 0.506133 | 2.828632 | 2.601212 | 0.031751 | low |
| TCGA-59-A5PD | 0.373432 | 1.883327 | 1.017574 | 1.019182 | 3.085739 | 0.488239 | 1.894099 | 0.276443 | 2.287608 | 0.213159 | 0.164844 | 0.834242 | 2.418954 | 2.968213 | 0.878709 | low |
| TCGA-13-0762 | 0.059243 | 3.907868 | 0.198062 | 0.277204 | 1.300531 | 0.25826 | 0.38562 | 0.130798 | 0.650074 | 0.270676 | 1.440008 | 0.159966 | 1.869599 | 5.245377 | 0.115785 | low |
| TCGA-09-1668 | 0.85615 | 3.922042 | 3.113946 | 3.425293 | 3.563484 | 0.330933 | 1.163804 | 0.965224 | 5.670932 | 0.228754 | 0.277701 | 1.29444 | 2.663437 | 3.590167 | 2.14651 | high |
| TCGA-23-1109 | 0.39146 | 2.004091 | 0.201191 | 0.842777 | 3.202553 | 0.201025 | 1.166426 | 0.667282 | 2.37927 | 0.057865 | 1.101599 | 0.690299 | 2.694668 | 4.382946 | 0.686364 | high |
| TCGA-20-1686 | 0.240791 | 1.849513 | 0.198053 | 0.113161 | 2.161535 | 0.284285 | 0.371035 | 0.141174 | 1.889602 | 1.116681 | 0 | 0.15434 | 1.945743 | 3.262783 | 0.635794 | high |
| TCGA-VG-A8LO | 0.174209 | 1.497647 | 0.255906 | 0.447023 | 0.844137 | 0.76705 | 0.68335 | 0 | 5.189781 | 0.301618 | 0.035594 | 1.312646 | 1.495907 | 4.44162 | 0.239523 | high |
| TCGA-23-1030 | 0.188362 | 2.488633 | 0.998588 | 0.973258 | 2.373964 | 0.087754 | 0.221348 | 0.306618 | 2.326832 | 0.270216 | 0.267326 | 0.634779 | 2.288575 | 4.308498 | 0.667908 | low |
| TCGA-24-2288 | 0.979008 | 3.984462 | 1.60941 | 2.128808 | 3.969917 | 0.520784 | 0.472714 | 0.793123 | 4.78866 | 0.140115 | 0.371831 | 1.338735 | 4.531427 | 4.839601 | 1.662736 | high |
| TCGA-57-1586 | 0.150181 | 2.7629 | 0.509161 | 0.251072 | 2.539564 | 1.372904 | 1.310155 | 0.360474 | 3.683684 | 1.332558 | 1.047936 | 0.581951 | 2.062007 | 5.224518 | 0.713313 | high |
| TCGA-13-1511 | 0.224446 | 2.132049 | 0.203535 | 0.272552 | 1.870362 | 0.608616 | 1.357011 | 0.39787 | 7.237054 | 0.107481 | 0 | 0.210334 | 1.450904 | 4.262035 | 0.227826 | high |
| TCGA-13-0766 | 0.542994 | 2.180911 | 0.292044 | 0.078593 | 3.082056 | 0.04249 | 0.082078 | 0.129681 | 3.949533 | 0.177471 | 0.112172 | 1.059818 | 1.123348 | 2.115163 | 0.369942 | low |
| TCGA-13-1498 | 0.629475 | 3.14313 | 1.125447 | 1.03335 | 3.707852 | 0.394887 | 0.244876 | 1.5022 | 4.949734 | 0.281476 | 0.352519 | 1.279847 | 2.613677 | 4.049287 | 1.361895 | high |
| TCGA-13-0800 | 0.026298 | 1.663091 | 0 | 0.103495 | 0.598071 | 0.196087 | 0.332531 | 0 | 1.254623 | 0.646281 | 0.58614 | 0.095738 | 1.031173 | 3.470864 | 0.427762 | low |
| TCGA-04-1519 | 0.137966 | 1.286019 | 0.076146 | 0.032157 | 0.581338 | 0.084292 | 0.705271 | 0.20346 | 1.059597 | 0.307014 | 0.441546 | 0.169622 | 1.568328 | 2.83238 | 0.098428 | low |
| TCGA-24-1431 | 0.326324 | 1.078801 | 0.330011 | 0.174111 | 2.533476 | 0.508213 | 0.716036 | 0.364349 | 2.474715 | 0.185806 | 0.043867 | 0.438293 | 1.836508 | 1.83043 | 0.264117 | high |
| TCGA-23-1119 | 0.512992 | 2.287213 | 0.469219 | 0.501418 | 3.649757 | 0.118145 | 0.442919 | 0.21551 | 3.59923 | 0.103995 | 0 | 0.572054 | 2.79917 | 3.260034 | 0.57492 | low |
| TCGA-10-0928 | 0.090889 | 0.950204 | 0.189637 | 0.121191 | 1.02879 | 0.108322 | 0.095888 | 0.135063 | 1.729792 | 0.118186 | 0.224952 | 0.359472 | 0.767839 | 3.053503 | 0.071536 | high |
| TCGA-25-1630 | 1.136422 | 1.819566 | 0.503399 | 0.746086 | 4.667086 | 0.20535 | 0.235156 | 0.765625 | 3.823495 | 0.185263 | 0.053273 | 0.783162 | 2.202594 | 3.028538 | 0.336756 | high |
| TCGA-13-0768 | 1.524502 | 5.116035 | 2.686176 | 3.506352 | 5.0183 | 0.266816 | 0.85397 | 2.016403 | 8.113533 | 1.139908 | 0.216145 | 4.09389 | 5.213801 | 4.877831 | 2.834166 | high |
| TCGA-24-1422 | 0.867934 | 2.741405 | 0.854394 | 0.895182 | 3.53698 | 1.180359 | 0.843533 | 1.729474 | 3.311006 | 0.198254 | 0.492393 | 1.003767 | 2.460104 | 5.034114 | 0.749408 | low |
| TCGA-25-1634 | 0.022425 | 0.943078 | 0 | 0 | 0.988572 | 0.109551 | 0.394571 | 0.050276 | 0.395784 | 0.199213 | 0.512799 | 0 | 1.141534 | 2.855366 | 0.052052 | high |
| TCGA-29-1695 | 0.716914 | 2.757751 | 0.609095 | 0.567451 | 4.610483 | 0.421804 | 0.97264 | 0.216495 | 3.385264 | 0.047958 | 0 | 0.512776 | 2.695482 | 3.111918 | 0.717279 | high |
| TCGA-30-1714 | 0.069913 | 0.354474 | 0.215242 | 0.047479 | 2.214011 | 0.169968 | 0.097567 | 0 | 1.574987 | 0.185095 | 0.068075 | 0.245124 | 0.487936 | 3.017312 | 0.173888 | high |
| TCGA-30-1891 | 0.272561 | 1.831764 | 0.478279 | 0.275224 | 3.575084 | 0.104077 | 0.437308 | 0.436246 | 2.177056 | 0 | 0.139047 | 1.155777 | 2.512267 | 1.792482 | 0.370297 | low |
| TCGA-13-1405 | 0.5859 | 2.578153 | 0.71216 | 0.570183 | 3.472447 | 0.619207 | 0.124996 | 0.882319 | 5.420601 | 0 | 0.321925 | 0.564727 | 2.460183 | 3.625663 | 0.919709 | low |
| TCGA-30-1860 | 1.604211 | 4.496474 | 2.690885 | 2.262146 | 5.273729 | 0.505288 | 0.107031 | 2.04764 | 5.357033 | 0.054175 | 0 | 1.911917 | 4.203389 | 2.463746 | 1.598337 | low |
| TCGA-36-1581 | 0.743492 | 5.32811 | 3.102 | 3.478476 | 4.290556 | 0.77181 | 0.369547 | 0.942737 | 5.021988 | 0.077683 | 0.113714 | 1.70166 | 5.220643 | 4.259259 | 2.625726 | high |
| TCGA-13-0893 | 0.840969 | 2.847389 | 0.732192 | 1.088605 | 3.150854 | 1.201431 | 0.511978 | 3.275911 | 5.706898 | 0.016867 | 0.195695 | 0.80877 | 2.133576 | 2.889882 | 1.242152 | low |
| TCGA-23-2077 | 0.23421 | 3.297149 | 0.803801 | 0.776202 | 3.837132 | 0.131077 | 0.121634 | 0.262454 | 2.443537 | 0.245037 | 1.639422 | 0.821292 | 2.327096 | 3.999568 | 0.427054 | low |
| TCGA-24-1923 | 0.444263 | 4.085069 | 0.342324 | 0.571419 | 2.635062 | 0.256515 | 0.067143 | 0.071796 | 1.656431 | 0.547547 | 0.941972 | 0.462877 | 1.326002 | 3.550375 | 0.264073 | low |
| TCGA-24-2036 | 0.106913 | 1.704011 | 0.047168 | 0.195508 | 1.16033 | 0.327199 | 1.157205 | 0 | 2.402209 | 0.149343 | 1.436492 | 3.070733 | 2.146966 | 4.390146 | 0.041053 | high |
| TCGA-13-0804 | 0.512364 | 2.713374 | 0.328684 | 0.364357 | 3.701532 | 0.184397 | 0.632976 | 1.901093 | 3.602241 | 0.437786 | 0.244163 | 0.613631 | 2.921378 | 3.863894 | 0.657887 | high |
| TCGA-13-1485 | 0.106015 | 2.109297 | 0.454844 | 0.803234 | 1.38603 | 0.578933 | 0.852686 | 0.060955 | 2.550914 | 0.718687 | 0.152248 | 5.782305 | 1.625646 | 4.776693 | 0.158647 | low |
| TCGA-23-1114 | 0.093246 | 3.19802 | 0.080924 | 0.216562 | 1.067194 | 0.584563 | 0.363962 | 0 | 0.664444 | 0.21136 | 0.27251 | 0.035487 | 0.899823 | 3.909919 | 0.358236 | high |
| TCGA-24-1469 | 0.382705 | 3.453423 | 1.219672 | 1.28266 | 2.90843 | 0.523185 | 0.475721 | 1.11712 | 2.941169 | 0.429721 | 0.915964 | 1.203514 | 2.660933 | 3.478397 | 1.243615 | low |
| TCGA-09-1673 | 0.650307 | 4.227365 | 0.269092 | 0.246173 | 2.676117 | 0.482664 | 0.306965 | 0.665425 | 3.402002 | 0.724657 | 1.385241 | 0.132972 | 1.530211 | 3.290396 | 0.246322 | low |
| TCGA-13-0887 | 0.066392 | 2.758652 | 0.492574 | 0.270548 | 2.022071 | 0.236199 | 0.922872 | 0.40037 | 5.072404 | 0.039591 | 0 | 1.038371 | 1.504328 | 3.626411 | 0.241524 | low |
| TCGA-25-2391 | 0.493293 | 2.240323 | 0.809677 | 0.598297 | 3.171482 | 0.238494 | 0.830472 | 0.532619 | 4.67467 | 0.19537 | 0.620535 | 0.451912 | 1.841812 | 3.02231 | 0.548543 | high |
| TCGA-24-1435 | 0.405729 | 2.63388 | 0.974286 | 1.020411 | 3.458077 | 0.261356 | 1.602378 | 0.527197 | 5.3602 | 0.221913 | 0.130675 | 1.837522 | 2.325974 | 3.132163 | 0.959354 | low |
| TCGA-13-1499 | 0.958391 | 3.585148 | 2.016007 | 2.097154 | 5.427582 | 0.51784 | 0.72524 | 1.776653 | 3.765644 | 0.306125 | 0.544863 | 1.764868 | 4.92515 | 3.612404 | 2.370855 | low |
| TCGA-24-2290 | 0.455263 | 3.271193 | 0.942438 | 1.185897 | 3.554507 | 0.18075 | 1.504083 | 0.11636 | 4.633857 | 0.081957 | 0 | 0.758671 | 1.890051 | 3.990421 | 0.597793 | high |
| TCGA-61-1995 | 1.057242 | 2.363053 | 1.624768 | 1.450659 | 3.768998 | 0.756261 | 2.529079 | 2.001694 | 2.958487 | 0.090404 | 0.07486 | 1.050826 | 2.85561 | 5.373266 | 1.05089 | high |
| TCGA-25-2398 | 0.54671 | 1.533707 | 0.697863 | 0.878334 | 3.36003 | 0.33936 | 0.556513 | 0.073346 | 3.112621 | 0 | 1.361339 | 0.860989 | 1.943904 | 3.720194 | 0.643304 | high |
| TCGA-57-1585 | 0.832323 | 1.9339 | 1.261108 | 1.122674 | 3.694352 | 0.371127 | 0.42733 | 0.707505 | 2.286452 | 0.052008 | 0.125244 | 1.075889 | 3.314199 | 2.996936 | 1.042719 | high |
| TCGA-61-2009 | 0.634576 | 1.96183 | 1.269352 | 1.275846 | 3.479438 | 0.283108 | 0.522498 | 0.203646 | 3.015162 | 0.037558 | 0.046257 | 0.953562 | 3.126718 | 3.632547 | 0.572283 | low |
| TCGA-13-1403 | 0.145633 | 1.159725 | 0.046533 | 0.193003 | 1.157961 | 0.245603 | 0.849447 | 0.566491 | 4.403729 | 0.728904 | 0.161079 | 1.09213 | 0.797339 | 3.807452 | 0.118236 | high |
| TCGA-13-1510 | 0.234915 | 2.281386 | 0.977242 | 1.250241 | 2.487325 | 0.21857 | 0.958542 | 0.937384 | 3.014796 | 0.14984 | 0.259015 | 1.443379 | 2.412882 | 3.049008 | 1.063604 | low |
| TCGA-04-1356 | 0.373124 | 2.022033 | 0.399238 | 0.463435 | 2.649357 | 0.138973 | 0.423997 | 0.172839 | 2.301025 | 0.301581 | 0 | 0.632667 | 2.877893 | 4.445921 | 0.62964 | low |
| TCGA-24-0966 | 0.253836 | 1.733793 | 0.337087 | 0.787349 | 2.156444 | 0.115091 | 0.54679 | 0.231682 | 4.072133 | 0.540621 | 0.48476 | 0.985932 | 2.537908 | 2.53776 | 0.331898 | low |
| TCGA-36-1576 | 1.203307 | 2.652702 | 1.744832 | 1.628637 | 4.771705 | 0.314912 | 0.279456 | 0.608976 | 3.526282 | 0.189747 | 0 | 1.157886 | 4.183881 | 3.255371 | 1.609133 | low |
| TCGA-24-1105 | 0.43882 | 2.955024 | 0.371434 | 0.883961 | 2.692715 | 0.36273 | 0.248354 | 0.559157 | 3.235483 | 0.080865 | 0.112894 | 0.303325 | 2.739375 | 4.755644 | 0.779072 | low |
| TCGA-23-1021 | 0.028803 | 1.810391 | 0.061519 | 0.076356 | 2.034602 | 0.061466 | 0.681526 | 0.672179 | 1.96905 | 0.212687 | 0 | 0.379456 | 1.1276 | 2.636954 | 0.079637 | high |
| TCGA-24-1467 | 0.379481 | 2.520675 | 0.608792 | 0.881374 | 2.148405 | 0.396572 | 0.034863 | 0.143773 | 2.050643 | 0.646302 | 0 | 0.517639 | 3.134986 | 4.735285 | 0.770303 | high |
| TCGA-61-1911 | 1.425212 | 4.497955 | 2.252601 | 2.070649 | 4.848627 | 0.581207 | 0.130227 | 1.740512 | 4.680009 | 0 | 0 | 1.550341 | 3.881553 | 4.116145 | 1.698682 | low |
| TCGA-24-1551 | 0.931089 | 3.209219 | 1.090897 | 1.211586 | 3.854606 | 0.486917 | 2.276589 | 1.298988 | 6.022567 | 0.019681 | 0.094737 | 0.982239 | 2.446715 | 4.327139 | 1.100998 | low |
| TCGA-13-0883 | 0.667022 | 2.19278 | 1.135239 | 1.436232 | 3.527911 | 0.525229 | 0.089109 | 0.386348 | 2.778787 | 3.09301 | 0.233879 | 2.558408 | 2.456592 | 3.715142 | 0.835681 | low |
| TCGA-24-2298 | 0.201576 | 3.128535 | 0.744065 | 0.945773 | 1.975752 | 0.32393 | 0.049934 | 0.359405 | 4.822956 | 0.173719 | 0.034666 | 0.551962 | 2.247138 | 5.134807 | 0.434756 | low |
| TCGA-24-1560 | 0.06176 | 1.663193 | 0.527012 | 0.613993 | 1.51474 | 0.3118 | 0.154444 | 0.299878 | 2.948353 | 0.338152 | 0.328109 | 0.700743 | 1.98336 | 3.733392 | 0.409524 | low |
| TCGA-5X-AA5U | 0.113919 | 1.064024 | 0.098958 | 0.092798 | 1.817135 | 1.349815 | 2.014074 | 0.103536 | 1.198952 | 0.17561 | 0.291449 | 0.126888 | 0.925251 | 3.577766 | 0.137769 | high |
| TCGA-23-1028 | 0.280128 | 1.2341 | 0.744288 | 0.701096 | 2.44811 | 0.269725 | 0.113415 | 0.265613 | 5.941859 | 0.078977 | 0.096953 | 0.97513 | 1.864555 | 2.076059 | 0.53003 | low |
| TCGA-09-1665 | 1.214324 | 3.087232 | 0.683572 | 0.918126 | 3.639688 | 0.151631 | 0.138522 | 0.630395 | 4.171805 | 0.037031 | 0 | 0.84818 | 2.260023 | 4.584136 | 0.590493 | low |
| TCGA-04-1361 | 0.473416 | 2.632476 | 0.590621 | 0.621814 | 3.323572 | 0.219554 | 1.018759 | 1.351432 | 4.606378 | 0.238742 | 0.263139 | 0.694025 | 2.168355 | 4.19714 | 0.82503 | low |
| TCGA-13-0916 | 0.531478 | 4.604412 | 1.214754 | 1.331069 | 3.222423 | 0.377103 | 0.337184 | 0.478283 | 4.991026 | 0.314768 | 1.150212 | 1.584557 | 4.029215 | 3.723555 | 1.078529 | high |
| TCGA-29-A5NZ | 0.219438 | 2.710242 | 1.453638 | 1.771104 | 2.074221 | 0.986234 | 1.877239 | 0.052681 | 2.187452 | 0.346812 | 1.412801 | 0.351318 | 2.898727 | 4.434046 | 1.023289 | high |
| TCGA-29-1774 | 0.26153 | 0.967596 | 0 | 0.054172 | 1.515123 | 0.243484 | 0.056596 | 0.174476 | 1.627381 | 0.510897 | 0.151229 | 0.145219 | 0.364743 | 2.396437 | 0.037924 | low |
| TCGA-23-1809 | 0.352384 | 2.266554 | 0.717977 | 0.703064 | 2.296813 | 0.236463 | 1.852415 | 0.480869 | 1.40366 | 0.376539 | 0.699078 | 0.436185 | 1.697058 | 4.430078 | 0.416489 | high |
| TCGA-24-2289 | 0.81422 | 3.487428 | 1.45941 | 2.007011 | 3.812981 | 0.49488 | 0.774317 | 0.348376 | 2.151464 | 0.7 | 0.093676 | 1.151456 | 2.794279 | 4.736118 | 1.156267 | high |
| TCGA-29-1768 | 0.836685 | 2.823073 | 1.006869 | 0.916408 | 4.634449 | 0.043436 | 0 | 1.523784 | 5.184983 | 0 | 0 | 1.155755 | 3.048564 | 1.231172 | 1.352927 | low |
| TCGA-61-1733 | 0.26653 | 2.734004 | 0.336865 | 0.703931 | 2.566195 | 0.159358 | 0.040672 | 0.243359 | 1.695964 | 0.173597 | 0.259967 | 1.19319 | 2.181375 | 3.139536 | 0.394054 | low |
| TCGA-61-2092 | 0.077375 | 0.407321 | 0.056132 | 0 | 1.423717 | 0.127624 | 0.361399 | 0.169579 | 5.643234 | 4.413524 | 0 | 0.095585 | 2.057653 | 3.373096 | 0.048874 | low |
| TCGA-29-1777 | 0.549654 | 1.653996 | 0.948787 | 0.375839 | 3.89476 | 0.289749 | 0.076498 | 0.485361 | 4.648856 | 0.097042 | 0 | 0.866812 | 2.778535 | 2.208856 | 0.621628 | low |
| TCGA-30-1892 | 0.44026 | 1.277039 | 0.072303 | 0.469598 | 2.878239 | 0.349203 | 0.160056 | 0.51688 | 2.328442 | 0.053082 | 0 | 1.539885 | 1.526049 | 2.252302 | 0.237 | low |
| TCGA-09-1662 | 1.077569 | 1.778008 | 0.716352 | 0.50239 | 3.88814 | 0.610835 | 1.707764 | 1.278252 | 2.849703 | 0.012952 | 0.123165 | 0.805074 | 2.459481 | 3.264058 | 0.703339 | low |
| TCGA-61-2098 | 0.062888 | 1.160741 | 0.089726 | 0.163612 | 0.989111 | 0.695766 | 0.735052 | 0.047709 | 0.3777 | 1.424712 | 0.760175 | 0.039414 | 1.375695 | 5.208538 | 0.12507 | high |
| TCGA-13-1409 | 0.042358 | 1.560654 | 0 | 0 | 0.434037 | 0.292885 | 0.812081 | 0.094173 | 1.314953 | 0.204836 | 0.989714 | 0.502575 | 1.3616 | 3.789702 | 0.049546 | low |
| TCGA-23-1026 | 0.5151 | 2.729783 | 1.143454 | 1.424657 | 3.529752 | 0.215455 | 0.290962 | 1.804002 | 5.384196 | 0.021047 | 0.149162 | 1.215419 | 2.729552 | 4.011472 | 1.2204 | low |
| TCGA-25-1628 | 1.449099 | 4.205238 | 2.393345 | 1.983719 | 4.21817 | 0.526093 | 1.720904 | 0.591251 | 6.287139 | 0.098286 | 0.881201 | 1.153166 | 3.912157 | 4.417017 | 1.936114 | low |
| TCGA-24-1419 | 0.218184 | 1.548716 | 0.01944 | 0.048027 | 0.865491 | 0.112797 | 0.20217 | 0.040447 | 0.107048 | 2.128773 | 0 | 0 | 0.518647 | 2.573983 | 0.025274 | high |
| TCGA-13-0888 | 0 | 1.928833 | 0 | 0.160757 | 0.204958 | 0.171026 | 0.113924 | 0.092213 | 0.494361 | 0.499156 | 0.648393 | 0.076384 | 1.085729 | 3.998956 | 0 | high |
| TCGA-36-1580 | 0.351347 | 2.94777 | 1.524301 | 1.130379 | 3.493499 | 0.13645 | 0.061726 | 0.491362 | 2.952394 | 0.134542 | 0.830386 | 1.804913 | 3.565539 | 2.398594 | 1.13939 | high |
| TCGA-61-1918 | 0.9507 | 2.537561 | 1.021456 | 1.358612 | 3.616993 | 0.753639 | 0.130842 | 0.954985 | 3.639187 | 0.238313 | 0.312731 | 2.0055 | 2.038494 | 3.663263 | 0.645183 | low |
| TCGA-13-1492 | 0.170722 | 2.972108 | 0.267278 | 0.789068 | 1.890504 | 0.464247 | 0.554926 | 0.079809 | 2.698643 | 0.042204 | 0.034847 | 0 | 1.293296 | 5.108874 | 0.646772 | high |
| TCGA-13-0885 | 0.895116 | 3.753192 | 1.79488 | 1.872798 | 4.418498 | 0.410369 | 0.409965 | 1.165024 | 5.670685 | 0.040989 | 0.146399 | 0.563249 | 3.312199 | 2.607748 | 1.35705 | low |
| TCGA-04-1655 | 0.042383 | 2.411473 | 0.154079 | 0.028695 | 0.461775 | 0.228478 | 0.081019 | 0.13913 | 0.091259 | 0.578376 | 1.293113 | 2.097718 | 4.702638 | 3.520881 | 0.029949 | high |
| TCGA-13-0720 | 0.028905 | 1.692653 | 0.232508 | 0.21864 | 1.846389 | 0.368715 | 0.454935 | 0.297571 | 7.132799 | 2.010349 | 0.109389 | 0.461951 | 1.967689 | 4.722296 | 0.239221 | low |
| TCGA-61-1907 | 0.753068 | 3.511942 | 2.029021 | 2.176261 | 5.248545 | 0.214032 | 0.009384 | 1.201624 | 3.929091 | 0 | 0 | 1.501235 | 3.935477 | 3.880049 | 1.323727 | low |
| TCGA-24-1562 | 0.538386 | 1.762287 | 0.652935 | 0.834932 | 3.477547 | 0.301544 | 1.603939 | 0.610375 | 3.799044 | 0.192227 | 0.070809 | 0.882505 | 2.397411 | 3.936034 | 0.731854 | high |
| TCGA-13-0725 | 0.268166 | 2.580794 | 0.697622 | 1.018695 | 4.000549 | 0.163818 | 0.667924 | 0.462965 | 3.653536 | 0.121372 | 0.051221 | 0.570314 | 2.42355 | 3.888739 | 0.512801 | low |
| TCGA-29-1707 | 0.429115 | 1.934375 | 0.838698 | 1.617018 | 4.425642 | 0.279322 | 0.472893 | 0.741738 | 8.806412 | 1.801172 | 0 | 0.326295 | 1.43543 | 4.642641 | 0.467798 | high |
| TCGA-24-1563 | 0.783537 | 3.271649 | 0.897935 | 0.681933 | 3.628131 | 0.37246 | 0.058258 | 0.807612 | 5.160229 | 0.06498 | 0 | 3.454431 | 2.00081 | 3.719094 | 0.605248 | low |
| TCGA-24-0970 | 0.153442 | 1.241144 | 0.165062 | 0.304803 | 2.174559 | 0.498417 | 1.274287 | 0.117274 | 2.624593 | 0.109125 | 0 | 0.246133 | 1.533498 | 4.426471 | 0.226025 | high |
| TCGA-04-1364 | 0.045017 | 0.545261 | 0.048565 | 0 | 0.500673 | 0.732316 | 0.819541 | 0.364028 | 0.239392 | 0.120859 | 0.693183 | 0.306182 | 0.329856 | 3.586511 | 0.455931 | high |
| TCGA-20-1687 | 0.589543 | 1.415425 | 0.466403 | 0.422367 | 3.107737 | 0.164857 | 0.926728 | 0.720321 | 4.582075 | 0.018995 | 0 | 0.493558 | 2.140546 | 3.048876 | 0.496095 | high |
| TCGA-25-1877 | 0.520771 | 2.101633 | 0.989928 | 1.318412 | 5.095643 | 0.149869 | 0 | 1.249635 | 4.051142 | 0.072263 | 1.010965 | 0.685293 | 2.491716 | 3.012401 | 0.584551 | high |
| TCGA-36-1568 | 0.626848 | 2.874035 | 0.961267 | 1.027819 | 3.143304 | 0.270993 | 0.868217 | 1.223195 | 2.947572 | 0.017077 | 0.12202 | 0.816505 | 2.839252 | 3.575747 | 0.736815 | high |
| TCGA-29-2428 | 0.721577 | 3.566771 | 1.830494 | 2.101852 | 4.364909 | 0.539222 | 0.343658 | 0.518154 | 4.763134 | 0.02153 | 0.24579 | 2.45553 | 4.426163 | 4.546428 | 1.7653 | low |
| TCGA-30-1862 | 0.639999 | 2.51039 | 0.993328 | 1.089931 | 3.333866 | 0.641091 | 0.694358 | 0.624528 | 2.933303 | 0.087908 | 0 | 0.802173 | 3.962159 | 5.48107 | 0.973328 | high |
| TCGA-04-1338 | 0.513733 | 3.0965 | 1.522941 | 1.593373 | 3.342678 | 0.184947 | 0.972845 | 0.147429 | 2.662148 | 0.129075 | 0.065225 | 0.881273 | 2.729787 | 1.875206 | 1.362678 | low |
| TCGA-59-2352 | 0.607962 | 4.312802 | 1.395285 | 1.270595 | 2.937163 | 0.346525 | 0.863754 | 0.650325 | 4.754315 | 0.548318 | 1.279773 | 0.7352 | 4.296316 | 3.408109 | 1.078039 | high |
| TCGA-29-1785 | 1.188772 | 4.856218 | 1.95975 | 1.873821 | 4.163753 | 0.492117 | 0.240596 | 1.235332 | 4.948393 | 0.160676 | 0.090425 | 1.81157 | 3.441739 | 2.910616 | 1.904481 | high |
| TCGA-24-1567 | 0.214544 | 1.57626 | 0.080971 | 1.883211 | 1.290301 | 0.089616 | 0.5565 | 0.111916 | 1.699359 | 0.020105 | 0 | 0.047152 | 0.753485 | 3.270811 | 0.082013 | low |
| TCGA-13-0765 | 0.151258 | 1.1061 | 0.243288 | 0.876953 | 2.772313 | 0.118553 | 0.302923 | 0.253981 | 3.671332 | 0.231606 | 0 | 2.055026 | 1.834751 | 3.943999 | 0.395145 | low |
| TCGA-24-1616 | 0.049798 | 0.689644 | 0.105504 | 0.088285 | 1.408676 | 0.127829 | 0.194518 | 0.277412 | 3.996754 | 2.110932 | 0.032501 | 0.310512 | 0.671759 | 2.522632 | 0.171584 | high |
| TCGA-09-1667 | 1.338293 | 4.004175 | 2.299722 | 2.416271 | 4.126073 | 0.551652 | 1.168629 | 1.850179 | 6.460168 | 0.243845 | 0.316125 | 1.65364 | 3.388689 | 4.182027 | 2.028107 | low |
| TCGA-59-2350 | 0.117609 | 2.744423 | 0.699418 | 0.830532 | 2.928131 | 0.488835 | 0.223493 | 0.157521 | 4.30718 | 0.111411 | 0.092369 | 0.529573 | 1.310368 | 2.885893 | 0.381589 | low |
